# Halogen Bonding Tetraphenylethene Anion Receptors: Anion‐Induced Emissive Aggregates and Photoswitchable Recognition

**DOI:** 10.1002/anie.202107748

**Published:** 2021-07-24

**Authors:** Andrew Docker, Xiaobo Shang, Daohe Yuan, Heike Kuhn, Zongyao Zhang, Jason J. Davis, Paul D. Beer, Matthew J. Langton

**Affiliations:** ^1^ Department of Chemistry University of Oxford Chemistry Research Laboratory Mansfield Road Oxford OX1 3TA UK

**Keywords:** aggregation-induced emission, anion recognition, fluorescence, halogen bonding, photoswitchable receptors

## Abstract

A series of tetraphenylethene (TPE) derivatives functionalized with highly potent electron‐deficient perfluoroaryl iodo‐triazole halogen bond (XB) donors for anion recognition are reported. ^1^H NMR titration experiments, fluorescence spectroscopy, dynamic light scattering measurements, TEM imaging and X‐ray crystal structure analysis reveal that the tetra‐substituted halogen bonding receptor forms luminescent nanoscale aggregates, the formation of which is driven by XB‐mediated anion coordination. This anion‐coordination‐induced aggregation effect serves as a powerful sensory mechanism, capable of luminescence chloride sensing at parts per billion concentration. Furthermore, the doubly substituted geometric isomers act as unprecedented photoswitchable XB donor anion receptors, where the composition of the photostationary state can be modulated by the presence of a coordinating halide anion.

## Graphical abstract

A series of halogen bonding (XB) tetraphenylethene (TPE) based anion receptors are reported. The receptors exhibit a remarkable anion‐induced turn‐on luminescence response, in which halogen bonding anion interactions drive the formation of emissive nanoscale aggregates. Disubstituted XB TPE derivatives function as photoswitchable anion receptors, in which modulation of photostationary states is intimately related to anion affinity.

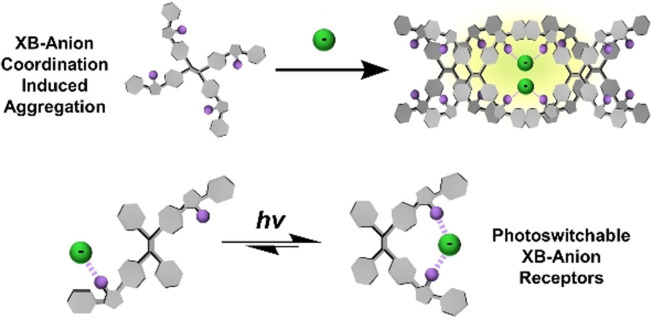

## Introduction

Organic luminescent materials frequently exhibit significant concentration dependent photophysical properties. Traditional chromophores, such as extended planar aromatic systems, are typically strongly fluorescent under dilute conditions, but suffer considerable attenuation in emissive properties with increasing concentration. This phenomenon is known as aggregation‐caused quenching (ACQ),[^1^, ^2^, ^3^] which is attributable, in most cases, to excimer or exciplex formation. Whilst undesirable, ACQ was widely accepted as an immutable characteristic of fluorescent material design. However, Tang's seminal report of aggregation‐induced emission (AIE) in a tetraphenylsilole derivative challenged this precept.^[4]^ Extensive experimental and theoretical studies revealed that the origin of this atypical fluorescent response upon aggregation was, amongst other factors, attributable to the restriction of intramolecular rotation which acts to suppress non‐radiative decay pathways of the excited state. Indeed, the discovery of this behaviour stimulated intense interest in the search for other AIE molecules, and recent years have seen an ever‐increasing library of AIE luminogenic (AIEgens) materials developed.^[5]^ In this context, tetraphenylethene (TPE) derivatives have risen to prominence as synthetically accessible, modular, multivalent scaffolds for AIEgens in the construction of bio‐imaging probes,[^6^, ^7^] optoelectronic devices^[8]^ and responsive materials.[^9^, ^10^, ^11^, ^12^] In addition to their role as highly sensitive luminescent reporter groups, TPE derivatives are also of interest by virtue of their potential to serve as photoswitchable platforms with large degrees of spatial control.^[13]^ TPE derivatives have also received significant interest in the development of supramolecular sensors which couple a luminescent response with molecular recognition events.[^14^, ^15^, ^16^, ^17^, ^18^] However, the considered employment of TPE‐derivatives within supramolecular host systems remains scarce,[^19^, ^20^, ^21^, ^22^] with the overwhelming majority of reported examples reliant on irreversible chemical modification of the AIEgen core to sense the guest species.^[23]^

Within the rapidly expanding field of supramolecular anion host‐guest chemistry, halogen bonding and chalcogen bonding intermolecular interactions have emerged as a valuable addition to the supramolecular toolbox.[^24^, ^25^, ^26^, ^27^] These sigma hole interactions frequently confer enhanced anion affinity and unique selectivity profiles relative to hydrogen bonding (HB) interactions within anion receptors,[^28^, ^29^, ^30^, ^31^, ^32^, ^33^, ^34^, ^35^, ^36^, ^37^] sensors[^38^, ^39^, ^40^] and transmembrane anion transporters.[^41^, ^42^, ^43^, ^44^]

Herein, we report a series of multidentate TPE‐based anion receptors containing highly potent perfluoroaryl iodo‐triazole XB donor motifs. Detailed ^1^H NMR anion titration experiments reveal the significant influence of XB donor geometry and multivalency on anion coordination mode and binding affinity. Photophysical experiments, in combination with dynamic light scattering (DLS) measurements, TEM imaging and X‐ray structure determination, reveal the formation of XB‐mediated anion‐coordination‐induced aggregates, accompanied by a significant turn‐on luminescence response which serves as a powerful transduction mechanism for chloride sensing. The bidentate iodo‐triazole TPE derivatives may be switched between strong and weak chloride binding states by photoisomerisation, representing—to the best of our knowledge—the first example of a photoswitchable XB anion receptor. Analysis of the photoswitching process reveals a strong correlation between halide anion affinity and the position of the photostationary state.

## Results and Discussion

### Synthesis of mono‐ and bi‐dentate anion receptors

**Scheme 1:**
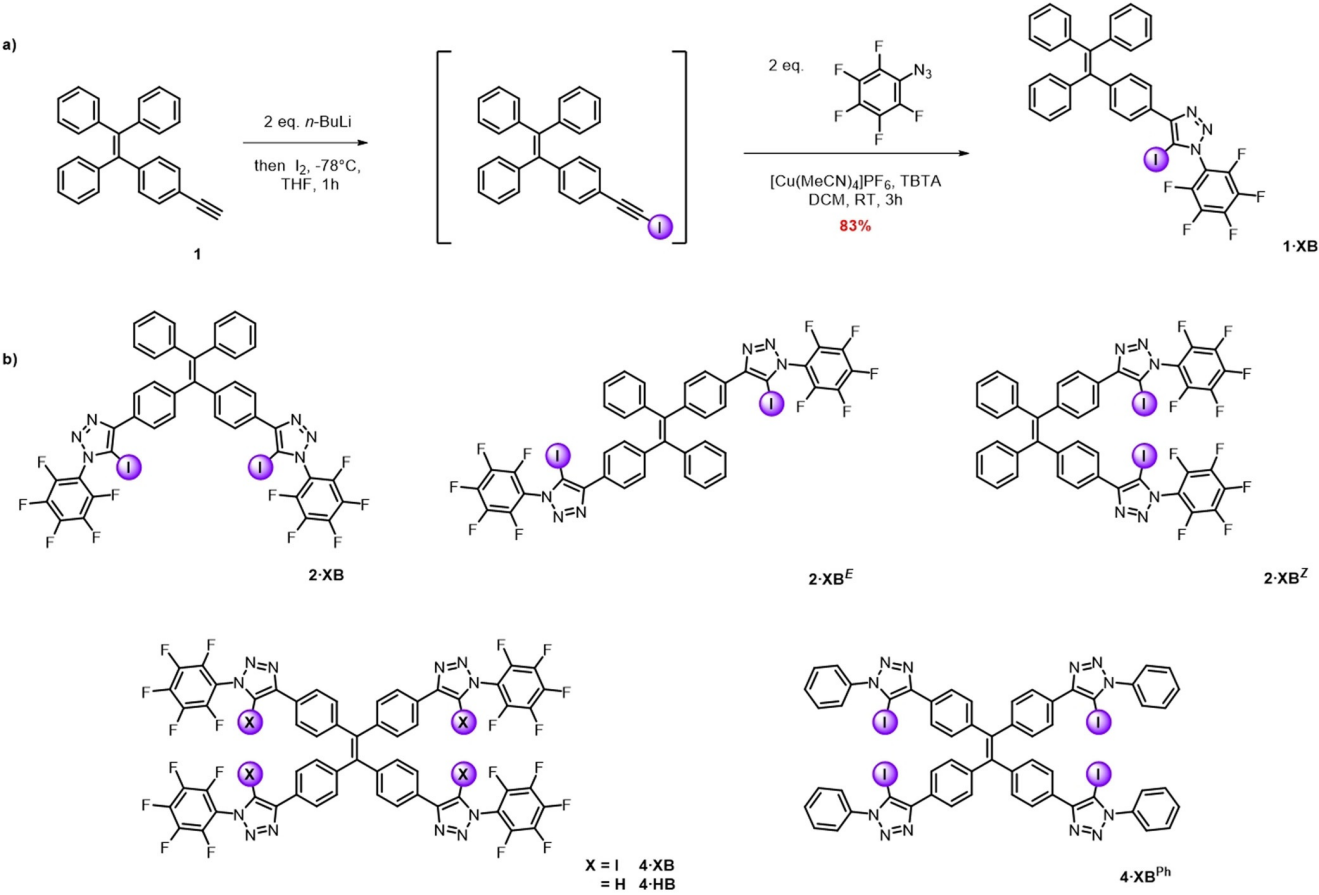
a) Representative synthesis of **1⋅XB**, via lithiation of proto‐alkyne **1**, subsequent iodination and CuAAC reaction with perfluorophenyl azide. b) Chemical structures of halogen bond donor TPE derivatives **2⋅XB**, **2⋅XB** ^***E***^, **2⋅XB^Z^**, **4⋅XB** and **4⋅XB^Ph^**, and the CH‐hydrogen bond donor **4⋅HB**.

To determine the effect of varying the geometrical arrangement of XB donor motifs around the TPE core on the multi‐dentate anion‐recognition behaviour, synthetic efforts were first directed towards preparing the mono‐ and di‐functionalised anion receptors **1⋅XB**, **2⋅XB**, **2⋅XB** ^***E***^ and **2⋅XB** ^***Z***^ (Scheme 1).

Typically, the synthesis of the target receptors was accomplished by treatment of the proto‐alkyne precursors with *n*‐BuLi in anhydrous THF solution at −78 °C, followed by reaction of the lithiated alkynes with iodine to afford the respective iodo‐alkynes (Scheme 1 a). The target receptors were subsequently synthesized via a copper(I)‐catalysed azide alkyne cycloaddition (CuAAC) reaction between the appropriate iodo‐alkyne derivative and perfluorophenyl azide, in the presence of Cu(MeCN)_4_PF_6_ and the Cu^I^ stabilising ligand tris(benzyltriazolylmethyl) amine (TBTA), which afforded **1⋅XB** and **2⋅XB** in yields of 83 % and 57 % respectively. In the case of **2⋅XB** ^***E***^ and **2⋅XB** ^***Z***^, a mixture of both isomers was obtained with a combined yield of 58 %, because the parent bis‐iodoalkyne was used as an inseparable mixture of the geometric isomers. Pleasingly, however, the introduction of the aryl‐iodotriazole motifs enabled resolution of the stereoisomers by silica gel column chromatography, facilitating the isolation of **2⋅XB** ^***E***^ and **2⋅XB** ^***Z***^ in 33 % and 25 % yield, respectively. Full synthetic procedures and characterisation data are available in the Supporting Information.

### Anion‐recognition experiments

With the series of mono‐ and di‐functionalised TPE receptors in hand, we examined their chloride anion‐recognition behaviour by ^1^H NMR anion titration experiments. Aliquots of chloride, as the tetrabutylammonium salt, were added to solutions of the respective receptor in [D_8_]THF, monitoring the binding induced chemical shift perturbations. In the case of **1⋅XB**, **2⋅XB** and **2⋅XB** ^***E***^, chloride binding induced a downfield shift of the aryl signal directly adjacent to the iodo‐triazole moiety, consistent with XB mediated anion complexation.

**Figure 1:**
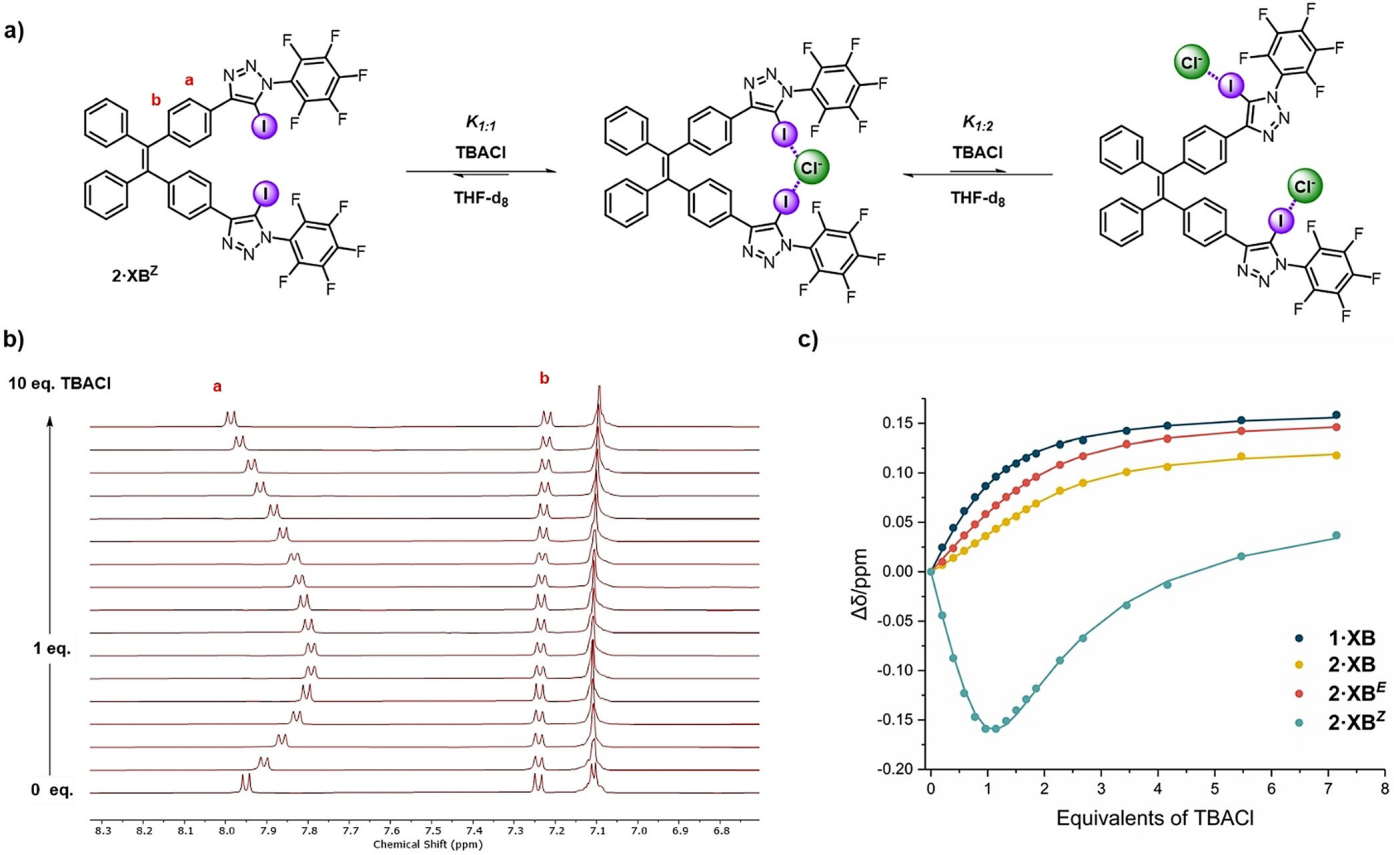
a) Postulated anion‐binding equilibrium for **2⋅XB** ^***Z***^. b) ^1^H NMR TBACl titration of **2⋅XB** ^***Z***^ ([D_8_]THF, 500 MHz, 298 K). c) Chloride binding isotherms for **1⋅XB**, **2⋅XB**, **2⋅XB** ^***E***^ and **2⋅XB** ^***Z***^, where circles represent experimental data and solid lines represent fitted data.

Analysis of the titration isotherm for **1⋅XB** using the Bindfit programme[^45^, ^46^] generated a 1:1 stoichiometric association constant, *K*_1:1_(**1⋅XB**)=2380 M^−1^, representing the binding affinity of chloride to one iodotriazole XB donor. The integration of additional XB donors to the TPE core enables higher host‐guest complex stoichiometries to be achieved, which in the case of **2⋅XB**, **2⋅XB** ^***Z***^ and **2⋅XB** ^***E***^ corresponds to a 1:2 host‐anion stoichiometry. Depending on the geometric arrangement of the two XB donors, anion complexation occurs with either no cooperativity (binding of the first anion does not affect binding of the second) or negative cooperativity (binding of the first anion inhibits binding of the second). In the former case, the 1:1 and 1:2 host‐guest binding constants, *K*_1:1_ and *K*_1:2_ respectively, are related by the expression *K*_1:1_=4 *K*_1:2_, accounting for statistical factors. Analysis of the chloride binding isotherms for the di‐iodotriazole derivatives **2⋅XB**, **2⋅XB** ^***Z***^ and **2⋅XB** ^***E***^ (Figure 1 c) revealed that the two XB donors in **2⋅XB** and **2⋅XB** ^***E***^ bind the chloride anions in a non‐cooperative manner, with an interaction parameter *α*=1, where *α*=4 *K*_1:2_/*K*_1:1_ (Table 1).

**Table 1:** Chloride association constants for mono‐ and disubstituted XB receptors in [D_8_]THF.

*K*_a_(Cl^−^)^[a]^ [M^−1^]	**1⋅XB** ^[b]^	**2⋅XB** ^[c,d]^	**2⋅XB** ^***E***[c,d]^	**2⋅XB** ^***Z***[c]^
*K*_1:1_	2380	5650^[f]^	4970	23200
*K*_1:2_	n/a	1410^[f]^	1240	960
*α* ^[e]^	n/a	1	1	0.17

[a] Chloride added as its tetrabutylammonium salt, errors <10 %. [b] Fitted to a 1:1 host‐guest binding isotherm. [c] Fitted to a 1:2 host‐guest binding isotherm. [d] Binding model assumes *K*_1:1_=4 *K*_2:1_. [e] Interaction parameter *α*=4 *K*_1:2/_ *K*_1:1_, with *α*=1 indicating no cooperativity, and *α*<1 indicating negative cooperativity. [f] Perturbation of the equilibrium due to anion‐induced aggregation effects not considered in the data fitting. For full details of the binding models and raw data see the SI, Table S1–3.

The chloride binding isotherm of **2⋅XB** ^***Z***^ exhibits a more complex profile, in which addition of up to 1 equivalent of chloride induced an upfield shift in proton *a* (Figure 1 b), after which an inflection point is observed and subsequent addition of chloride resulted in a downfield shift. This behaviour is consistent with the initial formation of a 1:1 complex, and the formation of a considerably weaker 1:2 complex at higher concentrations of chloride. The reversal of chemical shift perturbation is interpreted to be a consequence of conformational rearrangement upon changing from 1:1 to 1:2 stoichiometry to maximise the inter‐anion separation (Figure 1 a).

Analysis of the 1:1 stoichiometric binding constants for the three di‐iodotriazole geometric isomers **2⋅XB**, **2⋅XB** ^***E***^ and **2⋅XB** ^***Z***^ provided evidence for the presence or absence of bidentate anion binding with chelate cooperativity for each isomer. The chloride binding constant obtained for the reference mono‐dentate compound **1⋅XB**, *K*_1:1_(**1⋅XB**), quantifies the binding of the anion to a single iodotriazole binding site. The 1:1 binding constant for the di‐iodotriazole derivatives **2⋅XB**, **2⋅XB** ^***E***^ and **2⋅XB** ^***Z***^ is given by 2 *K*_1:1_(**1⋅XB**) in the absence of chelate cooperativity and when statistical factors associated with binding of one anion to a ditopic host are considered. Inspection of the values of *K*_1:1_ for the di‐iodotriazole derivatives reveals that, in the case of **2⋅XB** and **2⋅XB** ^***E***^, the two iodotriazole donors are too far apart to allow for chelation of the anion in a bidentate fashion: *K*_1:1_(**2⋅XB**) and *K*_1:1_(**2⋅XB** ^***E***^)≈2 *K*_1:1_(**1⋅XB**). In contrast, in **2⋅XB^Z^** the two iodotriazole derivatives are closer in space, which allows for effective bidentate cooperative binding of the first chloride anion, such that *K*_1:1_(**2⋅XB^Z^**)≈10*K*_1:1_(**1⋅XB**). The pronounced negative cooperativity in binding of the second equivalent of anion to **2⋅XB** ^***Z***^, reflected in the interaction parameter *α*=0.17, presumably therefore arises due to the combination of mutual anion‐anion electrostatic repulsion and the requirement to break the bidentate XB‐chloride interactions upon formation of the ternary complex.

Subsequent ^1^H NMR anion titration experiments of **2⋅XB** ^***Z***^ with the heavier halides revealed that bromide and iodide produced similar binding isotherms with somewhat attenuated association constants, consistent with anion basicity trends (Table 2).

**Table 2:** Halide association constants for **2⋅XB** ^***Z***^ in [D_8_]THF.

*K*_a_^[a]^ [M^−1^]	**Cl^−^** ^[b]^	**Br^−^** ^[b]^	**I^−^** ^[b]^
*K*_1:1_^[e]^	23200	8080	1050
*K*_1:2_	960	1330	40

[a] Anions added as their tetrabutylammonium salts, errors <10 %. [b] Fitted to a full 1:2 host‐guest binding model. For full details of the binding models and raw data see the ESI.

### Synthesis and anion recognition of tetradentate receptors

Attention then turned towards the synthesis of the tetra‐substituted receptors **4⋅XB**, **4⋅XB^Ph^** and **4⋅HB**, which were prepared from the corresponding tetra‐iodo/proto alkyne TPE derivatives using analogous procedures to those used for **1⋅XB** (see the Supporting Information for further details).

**Figure 2:**
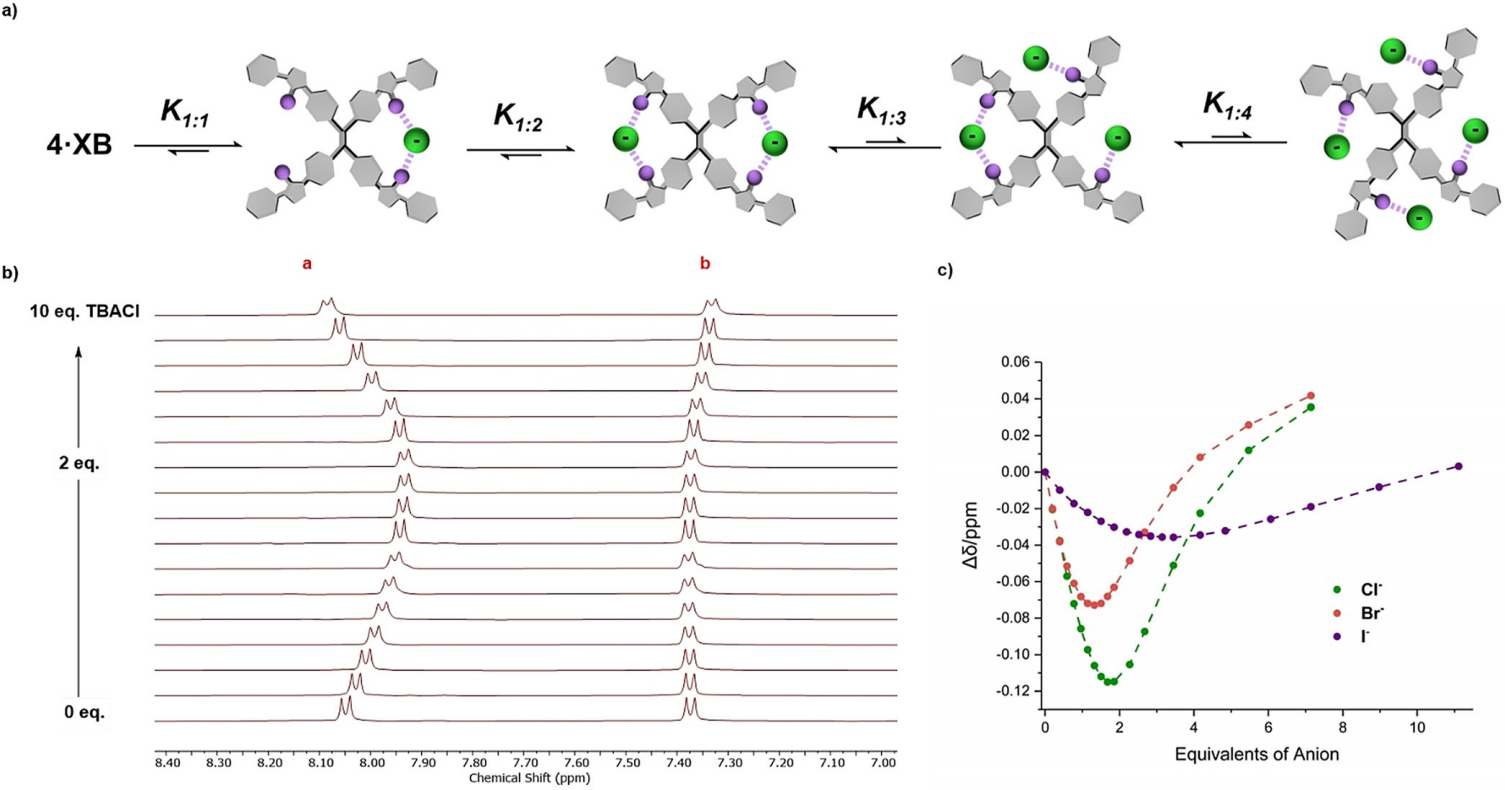
a) Postulated anion‐binding equilibrium for **4⋅XB**. b) ^1^H NMR TBACl titration of **4⋅XB** ([D_8_]THF, 500 MHz, 298 K). c) Halide binding isotherms for **4⋅XB**, where circles represent experimental data and dashed lines are a visual aid.

^1^H NMR chloride titration experiments were conducted on **4⋅XB**, **4⋅XB^Ph^** and **4⋅HB**, to elucidate the role and potency of XB interactions on the anion‐recognition process. Chloride binding titration experiments with **4⋅XB** revealed a chemical shift perturbation profile with an inflection point similar to that observed with **2⋅XB** ^***Z***^, in which up to two equivalents of TBACl induced an almost linear upfield perturbation of the aryl proton *a*, indicative of near quantitative formation of a 1:2 receptor:anion complex. Addition of further equivalents caused a progressive downfield shift, indicating the formation of higher receptor:anion stoichiometries, presumably up to 1:4 (Figure 2 b).^1^ Similar chemical shift perturbations were observed for **4⋅XB^Ph^**, whilst binding to the hydrogen bonding analogue **4⋅HB** was weak (see Supplementary information for further details). Furthermore, the preference for the lighter halides observed for **2⋅XB** ^***Z***^ appears to also translate to **4⋅XB**, upon visual analysis of the bromide and iodide binding isotherms (Figure 2 c).^2^ n1

### Solid‐state structure determination

**Figure 3:**
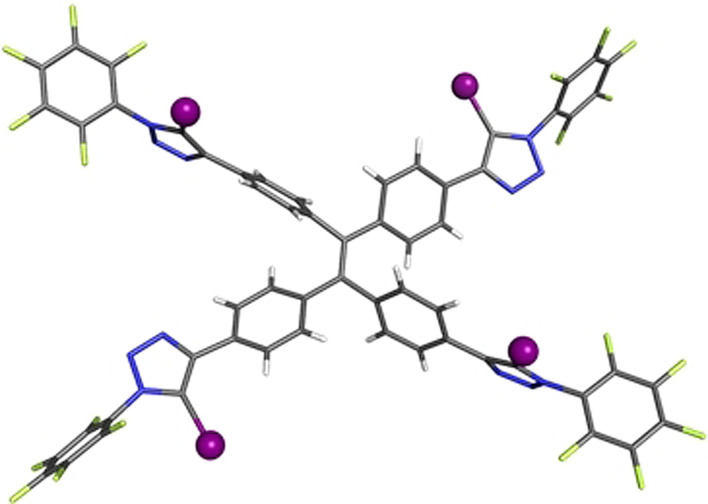
Solid state structure of **4⋅XB**. Grey=carbon, blue=nitrogen, white=hydrogen, light green=fluorine, purple=iodine.

**Figure 4:**
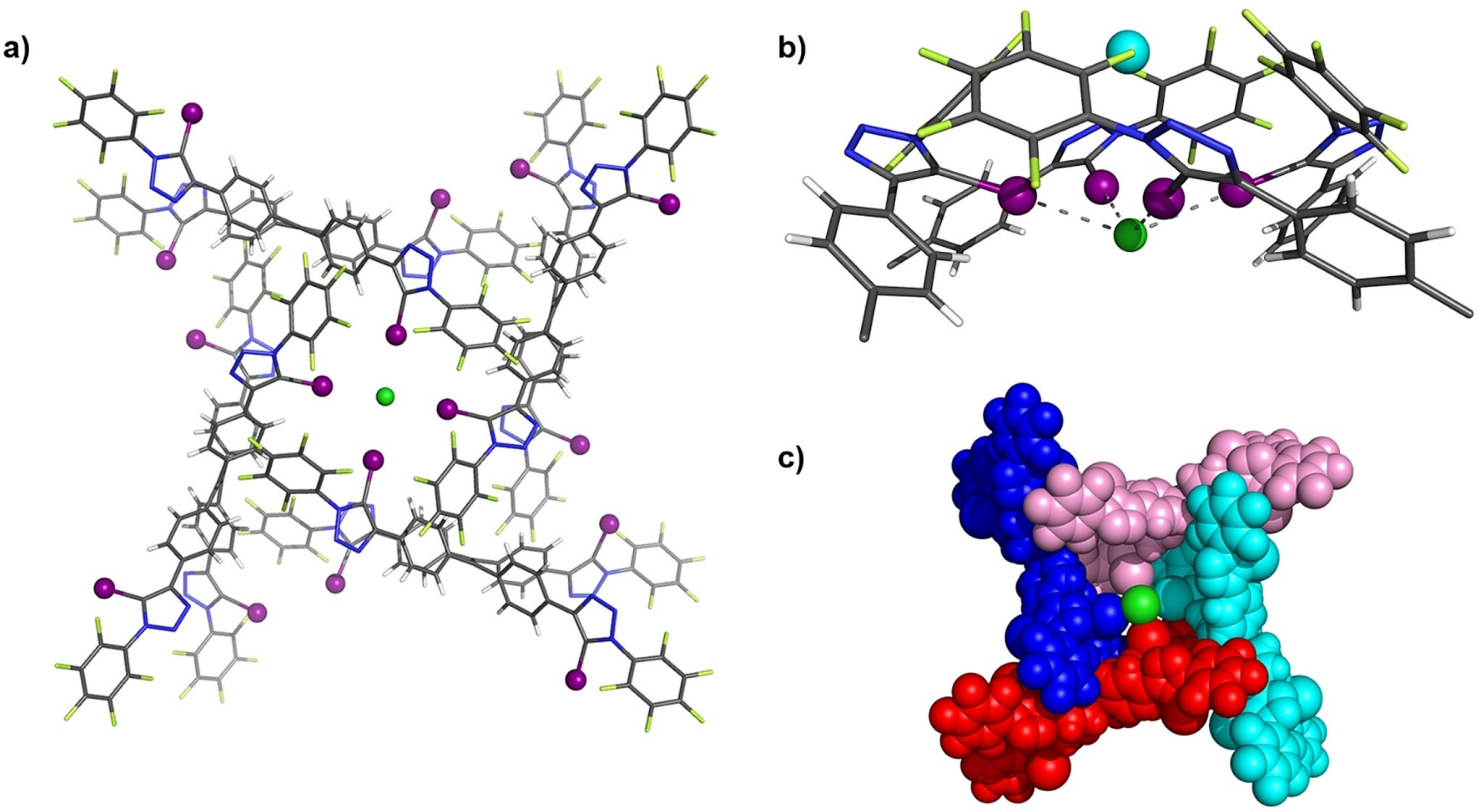
a) Solid state structure of **4⋅XB**⋅NaCl, presented as a wire frame structure. b) Truncated view of chloride coordination in **4⋅XB**⋅NaCl. c) Space‐filling representation of **4⋅XB**⋅NaCl. Grey=carbon, blue=nitrogen, white=hydrogen, light green=fluorine, purple=iodine, cyan=sodium, green=chlorine.

Further insight into the anion‐binding mode of the halogen bonding TPE systems was obtained by solid‐state characterisation of **4⋅XB** (Figure 3) and in the presence of a source of chloride anion (Figure 4).

It is noteworthy that repeated attempts to crystallize **4⋅XB** with a non‐coordinating organic countercation (e.g. tetraethylammonium, tetrabutylammonium or benzyltrimethylammonium) persistently yielded the NaCl complex of **4⋅XB** (Figure 4 a). The propensity of **4⋅XB** to crystalize with an adventitious sodium cation may be rationalized by inspection of the solid‐state structure in which the Na^+^ forms a proximal ion‐pair with a chloride anion simultaneously exhibiting four C−I⋅⋅⋅Cl^−^ XB interactions with four distinct **4⋅XB** molecules (Figure 4 b). Close examination of the structure reveals that the four‐fold coordination of chloride, in concert with other interactions (Figure S86), enforces close proximity of the TPE aromatic cores, as clearly visualised in the space filling representation (Figure 4 c). To establish the relative roles of the Na^+^ and Cl^−^ in the formation of these aggregated structures observed in the solid state, efforts were then directed towards obtaining structural information in the presence of other halides. Crystals suitable for X‐ray diffraction were obtained by slow diffusion of pentane into a 9:1 CHCl_3_:acetone (v/v) solution of **4⋅XB** in the presence of excess NaI. Single‐crystal X‐ray analysis revealed the NaI⋅**4⋅XB** complex displays an analogous four‐fold anion coordination via C−I⋅⋅⋅I^−^ XB interactions in an approximately square planar geometry around the iodide, however the sodium cation in this case resides in a vacancy between the central aromatic units constituting the tetraphenylethene core (Figure S82). This is presumably due to the reduced electrostatic interaction between the larger iodide anion and the sodium cation. The structural similarity of the obtained sodium halide complexes reveals the crucial role of potent XB‐anion interactions in the assembly of the aggregates. Encouraged by these findings, attention then turned to investigating whether these anion‐induced aggregates persist in solution phase.

### Anion‐coordination‐induced aggregation and anion sensing

**Figure 5:**
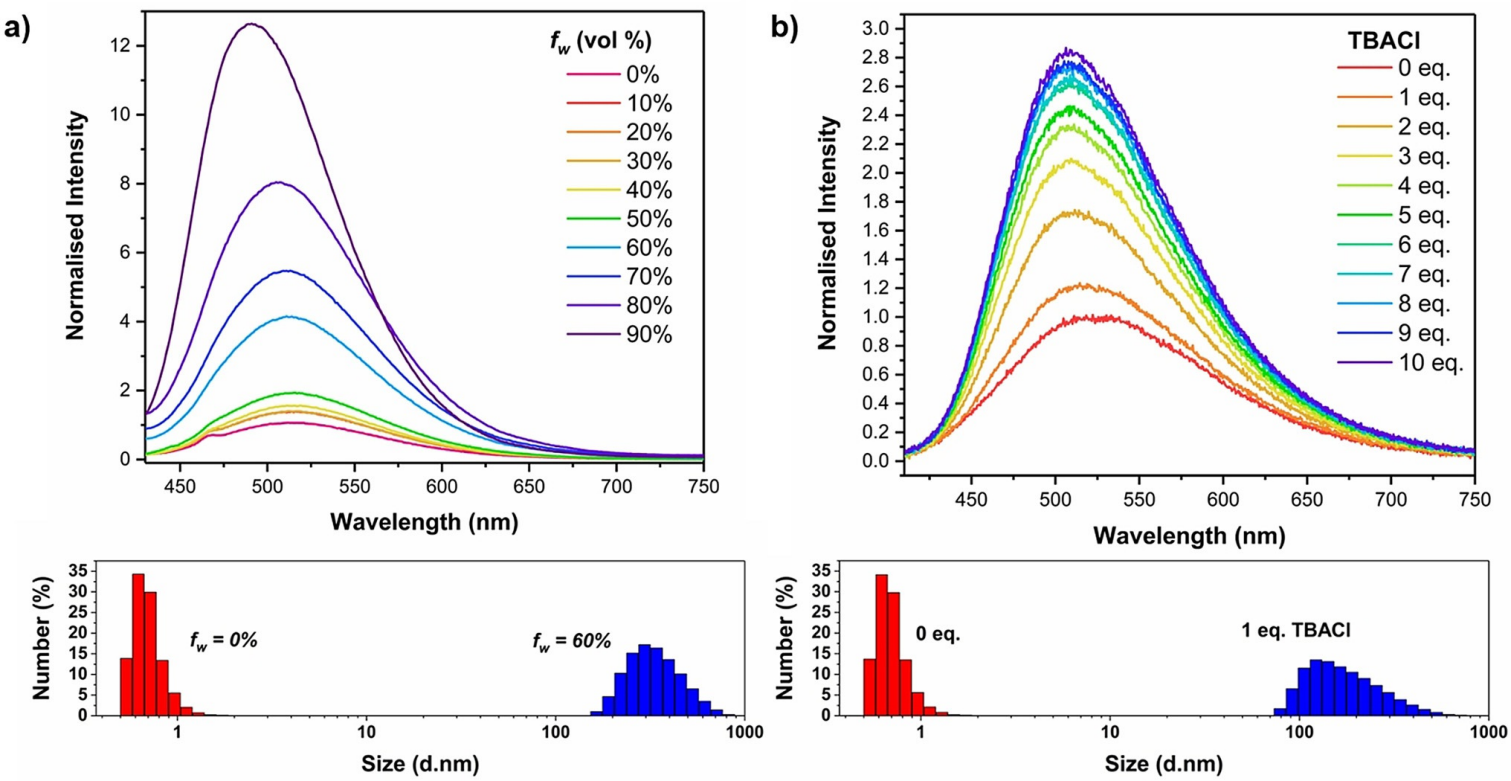
Fluorescence spectra (top) (THF, 10^−5^ M, *λ*_ex_=350 nm) and overlaid DLS measurements (bottom) of **4⋅XB** in the presence of (a) increasing water fraction (*f_w_*) and (b) Increasing equivalents of TBACl.

As anticipated from the well‐established emission behaviour of TPE derivatives, measurement of the fluorescence spectra in THF solution (10^−5^ M) revealed that **4⋅XB** is weakly emissive (*λ*_ex_=350 nm). A significant increase in emission intensity with increasing water fractions (*f*_w_) was observed at *f*_w_>60 %, indicative of the anticipated aggregation‐induced emission behaviour of TPE derivative **4⋅XB** (Figure 5 a, top), and the formation of aggregates confirmed by dynamic light scattering (DLS) measurements (Figure 5 a, bottom).

Addition of increasing equivalents of chloride to a THF solution of **4⋅XB** led to an increase in fluorescence intensity (*λ*_max_=520 nm), with a three‐fold increase observed after the addition of 10 equivalents (Figure 5 b, top), accompanied by a considerable hypsochromic shift (≈20 nm). Recent studies shedding light on the mechanism of AIE in TPE derivatives have demonstrated that the direction of *λ*_max_ perturbation can be closely correlated with the relative orientations of the central aromatic units to the alkene core. Increasing planarity between the constituent phenyl rings leads to increasing π‐conjugation and red‐shifting of the emission, whilst adoption of a perpendicular conformation decreases π‐conjugation and results in blue‐shifted emission.[^47^, ^48^] Close examination of the solid state structures for **4⋅XB** and its NaCl complex readily rationalise the observed changes in the emission profile, whereby the intermolecular coordination of the halide by halogen bond interactions not only induces the formation of closely intertwined complexes, restricting possible rotational degrees of freedom, but also enforces a more perpendicular geometry of the core aromatic scaffold (See Figure S85 and Table S5). Addition of the non‐coordinating anion hexafluorophosphate did not affect the emission spectrum, further demonstrating that XB‐mediated coordination to chloride is required to mediate aggregation (Figure S79). Dynamic light scattering (DLS) measurements of freshly prepared THF solutions (10^−5^ M) of **4⋅XB** indicate that the free receptor is non‐aggregated in the absence of chloride, but the addition of 1 equivalent of TBACl induces the formation of aggregates on the order of 100 nm in diameter (Figure 5 b, bottom). Further evidence for anion‐induced aggregate formation of **4⋅XB** was provided by transmission electron microscopy (TEM) which, consistent with results from DLS, revealed that the addition of 1 equiv. of TBACl to a 1 mM solution of **4⋅XB** in THF led to the formation of ≈100 nm sized particles, while no such species were observed for the free receptor in the absence of chloride (Figures S87 and S88).

**Figure 6:**
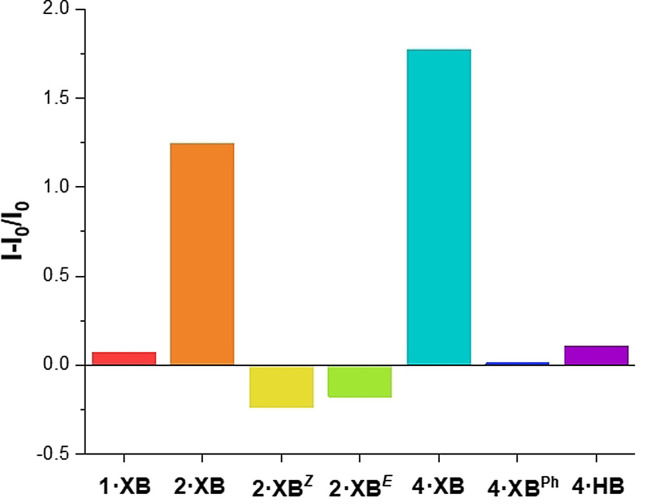
Relative fluorescence responses of the series of receptors in the presence of 10 equivalents of TBACl (THF, 10^−5^ M, *λ*_ex_=350 nm).

To further explore the role of spatial orientation and potency of the halogen bond donors in the formation of the anion‐mediated aggregates, the relative fluorescence response for all receptors was recorded in the presence of 10 equivalents of TBACl, and the data summarised in Figure 6. The largest emission intensity increases are observed for **4⋅XB** and **2⋅XB**, and the formation of aggregates in the presence of 1 equivalent of TBACl in both cases was confirmed by DLS measurements. In contrast, **1⋅XB**, **2⋅XB** ^***Z***^ and **2⋅XB** ^***E***^ exhibited minor perturbations in fluorescence intensity upon the addition of 10 equivalents of TBACl, and DLS analysis revealed no evidence of aggregated species. Together, these results confirm that the observed emission enhancement arises from XB‐mediated chloride induced aggregation. The absence of anion‐coordination‐induced aggregation with **2⋅XB** ^***Z***^, despite the receptor possessing enhanced chloride affinity relative to all other mono‐ and di‐substituted TPE derivatives, demonstrates that spatial orientation of the XB donors is crucial in the formation of the chloride induced XB aggregates. Under analogous conditions **4⋅XB^Ph^** and the hydrogen bonding analogue **4⋅HB** also demonstrated no significant emission intensity change, providing further strong evidence for the requirement for multiple potent XB‐anion interactions to drive aggregate formation.

**Figure 7:**
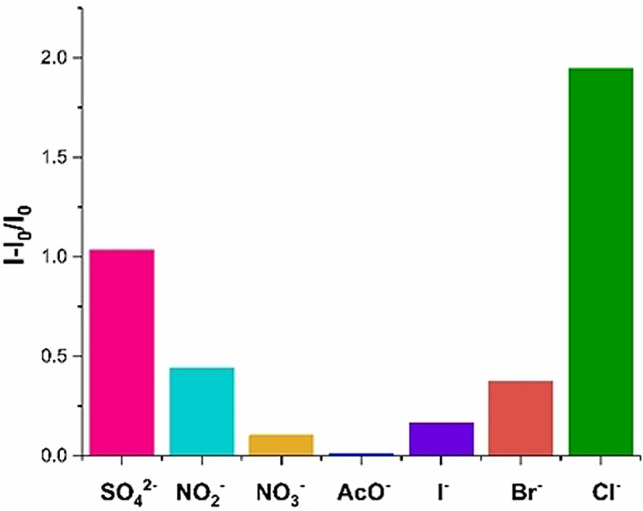
Relative fluorescence response for **4⋅XB** in the presence of 10 equivalents of a range of anions (THF, 10^−5^ M, *λ*_ex_=350 nm).

Analogous experiments were also undertaken to investigate how the nature of the anionic guest influences aggregate formation, in which the emission intensity of **4⋅XB** in THF was measured in the presence of 10 equivalents of a range of anions (Figure 7). In all cases except acetate, the addition of an anion induced a fluorescence intensity increase. In accordance with the anion basicity trend this increase followed the order of Cl^−^ > Br^−^ > I^−^. Notably, despite its di‐anionic nature, SO_4_^2−^ elicited a diminished enhancement in emission intensity in comparison to the strong chloride response. The considerable fluorescence response for Cl^−^ corresponds to a limit of detection (LOD) of 5.14 μΜ (183 ppb), constituting one of the most sensitive supramolecular anion sensors to‐date (Figure S75).[^23^, ^49^]

### Photo‐switchable anion recognition

In addition to the highly desirable luminescent properties of TPE, unsymmetrical doubly substituted TPE derivatives have the capability to function as molecular photo‐switches in which photoisomerisation switches between different spatial arrangements of functional groups. Photo‐switchable anion receptors in particular have attracted interest for applications including ion extraction and transport, enabling control over when and where the hosts bind or release their targets.^[50]^ However, the utilization of TPE as a photoswitch has been hindered by practical issues in their synthesis, due to a combination of poor stereoselectivity and challenging chromatographic separation of the closely related geometric isomers. Consequently, the vast majority of reports to date using these di‐functionalised TPE derivatives in material or polymer sciences have used mixtures of the isomers. Compounds **2⋅XB** ^***E***^ and **2⋅XB** ^***Z***^ are prime candidates for developing photo‐switchable anion receptors due to the contrast in chloride binding affinity of the two geometric isomers (*K*_1:1_ of 4970 and 23 200 M^−1^, respectively), and fortuitously, could be isolated by column chromatography.

**Figure 8:**
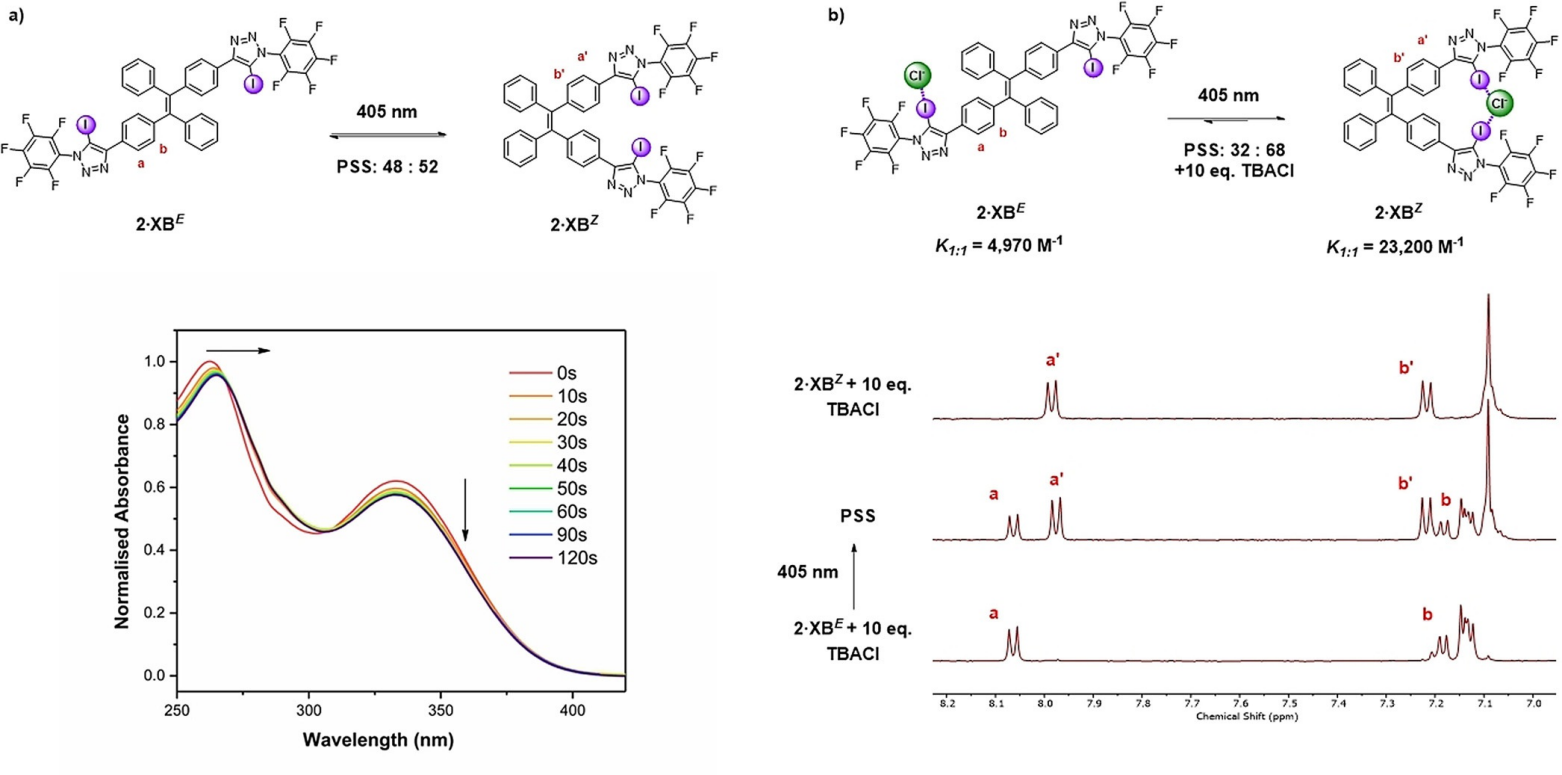
a) Photoisomerisation of **2⋅XB** ^***E***^. UV/Vis spectra of the photo‐stationary state generated upon irradiation of **2⋅XB** ^***E***^ with 405 nm light (THF, 10^−5^ M^−1^). b) Photoisomerisation of **2⋅XB** ^***E***^ in the presence of 10 equivalents of TBACl (^1^H NMR, [D_8_]THF, 500 MHz, 298 K).

Irradiation of a solution of a pure sample of **2⋅XB** ^***E***^ with 405 nm light triggered photo‐isomerisation, generating an approximately equal ratio of both isomers in the photo‐stationary state (52:48 **2⋅XB** ^***Z***^:**2⋅XB** ^***E***^), as determined by integration of the NMR signals of the mixture. The effect of 405 nm irradiation on the UV vis spectrum of a solution of **2⋅XB** ^***E***^ is shown in Figure 8 a, resulting in a bathochromic shift of the absorption at low wavelength (ca. 265 nm) and a decrease in the absorption at 335 nm, with isosbestic points at 270 nm and 310 nm confirming the unimolecular nature of the isomerisation process. The photo‐stationary state was reached within 120 s, as deduced by the absence of further changes in the absorption spectrum. No thermal isomerization of **2⋅XB** ^***Z***^ or **2⋅XB** ^***E***^ was observed under ambient conditions in THF solution over the course of the experiment when excluded from light. Analogous irradiation experiments starting with **2⋅XB** ^***Z***^ generated a similar, but reversed set of perturbations in the UV‐vis profile, namely an increase in absorption at 335 nm and a hypsochromic shift in the local *λ*_max_ absorption at approximately 265 nm.

Given the contrasting anion‐binding properties of the isomers, we anticipated that the photostationary state may be biased in favour of the *Z* isomer by coordination to an anion. To this end, analogous photoisomerisation experiments were conducted by irradiating the sample with 405 nm light in the presence of 10 equivalents of TBACl. In the presence of the halide, the PSS is biased in favour of the *Z* isomer, due to strong XB coordination to the chloride (68:32 **2⋅XB** ^***Z***^:**2⋅XB** ^***E***^) (Figure 8 b). Similar experiments were undertaken with 10 equivalents of bromide and iodide (Table 3), revealing that the bias of the PSS in favour of the *Z* isomer correlates with the anion‐binding affinity to **2⋅XB** ^***Z***^, namely Cl^−^>Br^−^>I^−^. In contrast, in the presence of non‐coordinating anion, PF_6_^−^, which demonstrated no binding to either receptor in ^1^H NMR titration experiments, no perturbation of the position of the PSS was detected. Together, this data provides evidence for photo‐switchable XB‐mediated anion recognition, in which strong binding to the **2⋅XB** ^***Z***^ and weaker binding to **2⋅XB** ^***E***^ enables switching of binding using light, as well as modulation of the obtained ratio of isomers in the photostationary state.

**Table 3:** PSS ratios determined from irradiation of 1 mM of **2⋅XB** ^***Z***^.

Anion^[a]^	PSS Ratio (**2⋅XB** ^***Z***^/**2⋅XB** ^***E***^)^[b]^
None	52:48
Cl^−^	68:32
Br^−^	62:38
I^−^	58:42
PF_6_^−^	53:47

[a] 10 equivalents of anion added as their tetrabutylammonium salts, 10^−3^ M, [D_8_]THF. [b] PSS ratios determined from ^1^H NMR integration.

## Conclusion

In conclusion, we report a series of halogen bonding TPE‐based anion receptors, in which the multivalency and relative orientation of the strongly potent perfluoroaryl XB donors is systematically investigated. ^1^H NMR anion titration experiments elucidate the varied binding modes and stoichiometries of the family of halogen bonding TPE derivatives. Fluorescence anion titration experiments demonstrate that the tetra‐substituted perfluorinated **4⋅XB** receptor, experiences a dramatic increase in emission intensity upon the addition of chloride, whilst the non‐fluorinated **4⋅XB^Ph^** or hydrogen bonding **4⋅HB** analogues elicit no photophysical response. Detailed analysis of the fluorescence spectra, dynamic light scattering measurements, TEM imaging and X‐ray crystal structure determination demonstrate that the origin of this sensory response is an unprecedented XB‐anion coordination‐induced aggregation mechanism. Furthermore, successful isolation of difunctionalised geometric isomers **2⋅XB** ^***Z***^ and **2⋅XB** ^***E***^ facilitated the investigation of these TPE derivatives as photoswitchable anion receptors, in which it was demonstrated that the position of the photostationary state could be significantly biased towards **2⋅XB** ^***Z***^ on the basis of enhanced halide anion affinity. These results not only provide further evidence for the advantages of XB interactions in the design of potent anion receptors, but also point to new opportunities in the development of XB anion‐responsive materials, switches and sensors.

## Conflict of interest

The authors declare no conflict of interest.

## Supporting information

As a service to our authors and readers, this journal provides supporting information supplied by the authors. Such materials are peer reviewed and may be re‐organized for online delivery, but are not copy‐edited or typeset. Technical support issues arising from supporting information (other than missing files) should be addressed to the authors.

Supporting Information

## Footnotes

n2 The chloride titration experiments were repeated at concentrations of **4⋅XB** spanning 0.25–1.0 mM. The general form of the binding isotherms were unchanged: stronger initial binding of the first two equivalents of anion, an inflection point at ≈2 equivalents, and weaker binding of the subsequent anions. This suggests that the formation of aggregates, which are resolved by fluorescence, DLS and TEM experiments but not by NMR, do not significantly affect the overall form of the titration, but do hinder a quantitative analysis of binding affinities.

n1 The binding data could not be reliably fitted to a 1:4 host:guest isotherm, presumably due to the presence of anion‐induced aggregates. However, visual inspection of the isotherm (noting the inflection point at 2 equiv. of anion) indicates that the binding of the first and second anion is strong (*K*≥10^4^ M^−1^, likely via the same bidentate binding mode seen in **2⋅XB^Z^**), with weaker binding of the third and fourth equivalent.
