# Supplementary material for: Halogen Bonding Tetraphenylethene Anion Receptors: Anion‐Induced Emissive Aggregates and Photoswitchable Recognition
Source: Angew Chem Int Ed Engl. 2021 Jul 24;60(35):19442–50. doi: 10.1002/anie.202107748 (PMC8456845; doi:10.1002/anie.202107748)
Supplement: Supplementary file 1 — Supporting Information [file ANIE-60-19442-s001.pdf]

Angewandte Chemie
Eine Zeitschrift der Gesellschaft Deutscher Chemiker

Supporting Information

Halogen Bonding Tetraphenylethene Anion Receptors: Anion-Induced Emissive Aggregates and Photoswitchable Recognition

Andrew Docker+, Xiaobo Shang+, Daohe Yuan, Heike Kuhn, Zongyao Zhang, Jason J. Davis, Paul D. Beer, and Matthew J. Langton**

anie_202107748_sm_miscellaneous_information.pdf

Contents

1. Materials and methods 2
2. Synthetic Procedures and Characterisation 3
3. ^{1}H NMR Titration Experiments 30
4. DLS experiments 49
5. Absorption and fluorescence experiments 59
6. Crystal Structure Determination 72
7. Transmission Electron Microscopy (TEM) Imaging 79
8. References 80

1. Materials and methods

All solvents and reagents were purchased from commercial suppliers and used as received unless otherwise stated. Dry solvents were obtained by purging with nitrogen and then passing through an MBraun MPSP-800 column. H_2O was de-ionized and micro filtered using a Milli-Q ® Millipore machine. Column chromatography was carried out on Merck® silica gel 60 under a positive pressure of nitrogen. Routine NMR spectra were recorded on either a Varian Mercury 300, a Bruker AVIII 400 or a Bruker AVIII 500 spectrometer with ^{1}H NMR titrations recorded on a Bruker AVIII 500 spectrometer. TBA salts were stored in a vacuum desiccator containing phosphorus pentoxide prior to use. Where mixtures of solvents were used, ratios are reported by volume. Chemical shifts are quoted in parts per million relative to the residual solvent peak. Mass spectra were recorded on a Bruker µTOF spectrometer. Triethylamine was distilled from and stored over potassium hydroxide. Tris[(1-benzyl-1H-1,2,3-triazol-4-yl)methyl]amine (TBTA). DLS analysis was performed on Malvern Zetasizer Nano with a 532 nm laser as the light source. Phenyl azide[1] and pentafluorophenyl azide[2] were prepared according to literature procedures. Fluorescence spectroscopic data were recorded using a Horiba Duetta fluorescence spectrophotometer, equipped with Peltier temperature controller and stirrer. UV-Vis spectra were recorded on a V-770 UV-Visible/NIR Spectrophotometer equipped with Peltier temperature controller and stirrer using quartz cuvettes of 1 cm path length. Experiments were conducted at 25°C unless otherwise stated. Photo-irradiation of liquid samples was carried out using a 1W Thorlabs high-power mounted LED (M405L4) using in-house custom built set-ups using optical components supplied by Thorlabs, as previously reported.[3] Samples were irradiated for sufficient time to reach the photo-stationary state, as confirmed by NMR or UV-vis experiments. Transmission Electron microscopy (TEM) images were obtained using a FEI Tecnai 12 Transmission Electron Microscope (120 kV), samples were a stained with 2% uranyl acetate.

2. Synthetic Procedures and Characterisation

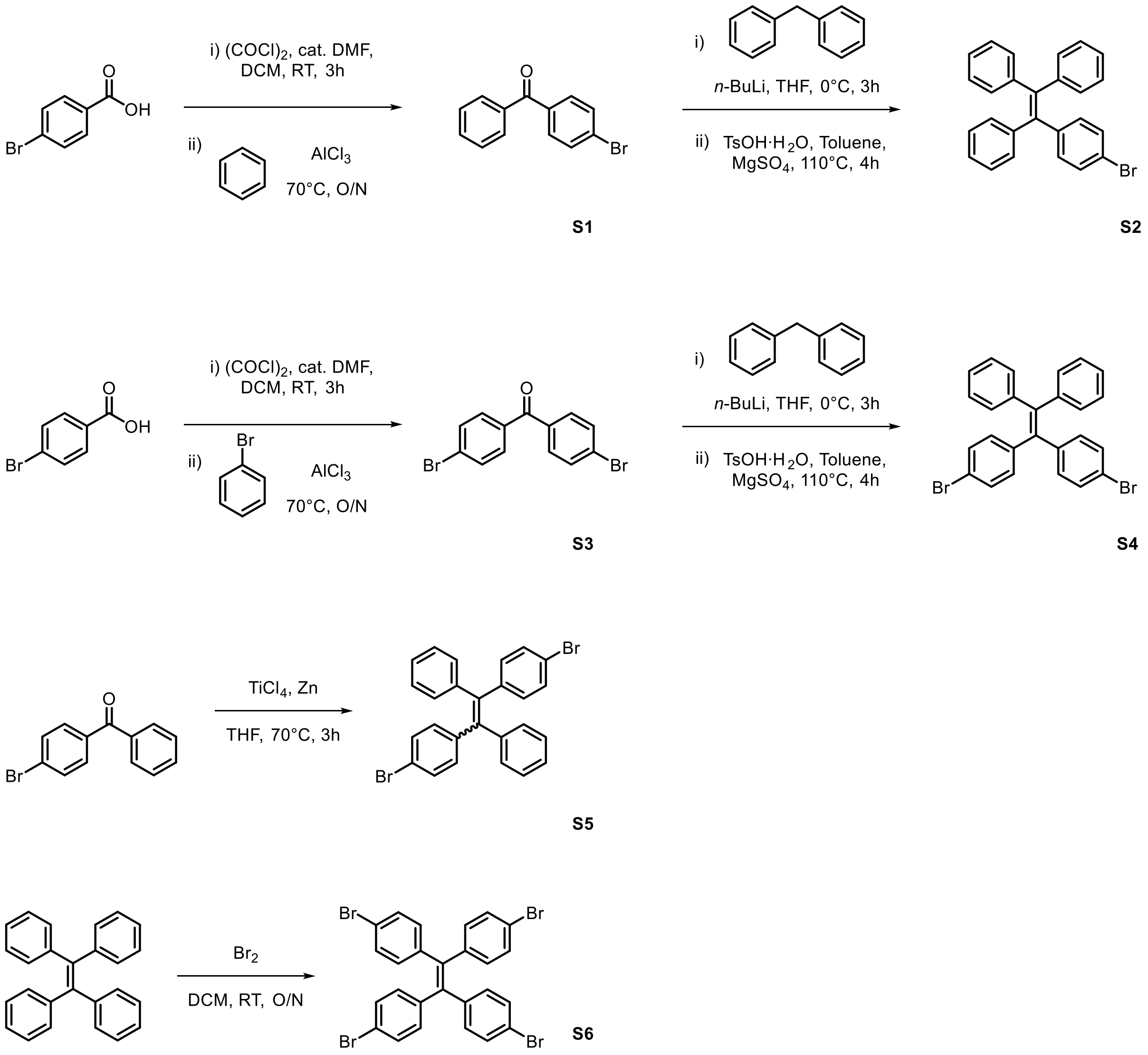

Scheme S1. Synthesis of ketone precursors and brominated tetraphenylethylene derivatives**.**

S1 and **S3** were synthesised according to General Procedure 1.[4]
S2 and **S4** were synthesised according to General Procedure 2.[5]
S5 was synthesised according to Procedure 3.[6]
S6 was synthesised according to Procedure 4.[7]

General Procedure 1

The appropriate benzoic acid (30 mmol) was suspended in anhydrous CH_2Cl_2 (50 ml) to which was added oxalyl chloride (25.7 ml, 300 mmol) and 3 drops of DMF, the reaction mixture was stirred under ambient conditions until homogenous. After which the mixture was concentrated to dryness, the resultant solid was dissolved in either benzene or bromobenzene (50 ml) to which was added $AlCl_3$ (4.00 g, 30 mmol), the reaction mixture was heated to 70°C for approximately 16 hours. After which time the reaction mixture was carefully quenched by the addition of H_2O (50 ml) and diluted with CH_2Cl_2 (300 ml) the organic phase was collected and washed with H_2O (3 × 50 ml). The organic phase was dried over $MgSO_4$ and the solvent removed in vacuo, the crude mixture was purified by silica gel column chromatography (CH_2Cl_2), affording a white solid.

General Procedure 2

To a THF solution (50 ml) of diphenylmethane (9.00 g, 53.5 mmol) at 0°C was added a 1.6 M solution of n-BuLi in hexanes (36.8 ml, 58.9 mmol) and left to stir at that temperature for 1 hour. After which a THF solution (50 ml) of the appropriate ketone (53.5 mmol) was added dropwise and left to stir at room temperature for 3 hours. After which time the reaction mixture was quenched by the addition of H_2O (1 ml) and concentrated *in vacuo* to an oily residue, which was resuspended in toluene (ca. 100 ml) and to this mixture was added $TsOH \cdot H_2O$ (ca. 1g) and $MgSO_4$ (ca. 1g). This mixture was refluxed for ca. 4 hours until determined complete by TLC (eluent CH_2Cl_2). After which time the reaction mixture was allowed to cool and diluted with CH_2Cl_2 (300 ml) washed with H_2O (2 × 100 ml) dried over $MgSO_4$ and concentrated to an oily residue *in vacuo*, the crude mixture was subjected to silica gel column chromatography (CH_2Cl_2; hexane, 1:1 v/v), affording a white solid.

Procedure 3

4-bromobenzophenone (2.61 g, 10.0 mmol), freshly activated Zn dust (1.31 g, 20.0 mmol) were suspended in anhydrous THF (50 ml) and cooled to 0°C. To this mixture $TiCl_4$ (1.12 ml, 10.0 mmol) was added dropwise, after which the mixture was heated to 70°C for 3 hours. Upon cooling the mixture was carefully quenched with H_2O (50 ml), diluted with CH_2Cl_2 (300 ml) and the organic phase washed with H_2O (2 × 100 ml), dried over $MgSO_4$ and concentrated to an oily residue *in vacuo*, the crude mixture was subjected to silica gel column chromatography (CH_2Cl_2; hexane, 1:1 v/v), affording a white solid consisting of approximately a 1:1 mixture of the *E* and *Z* isomers.

Procedure 4

Tetraphenylethylene (10 g, 30.1 mmol) was dissolved in CH_2Cl_2 (400 ml), to which was added Br_2 (10 ml, 0.389 mol) after stirred overnight under ambient conditions. The reaction was diluted with CH_2Cl_2 (400 ml) and washed with $Na_2S_2O_{3(aq)}$ solution until decolourised, after which the organic phase was copiously washed with water, dried over $MgSO_4$ and concentrated to a white solid which was subsequently recrystalised from boiling hexanes to afford the crystalline target product.

Synthesis of Alkyne-TPE precursors:

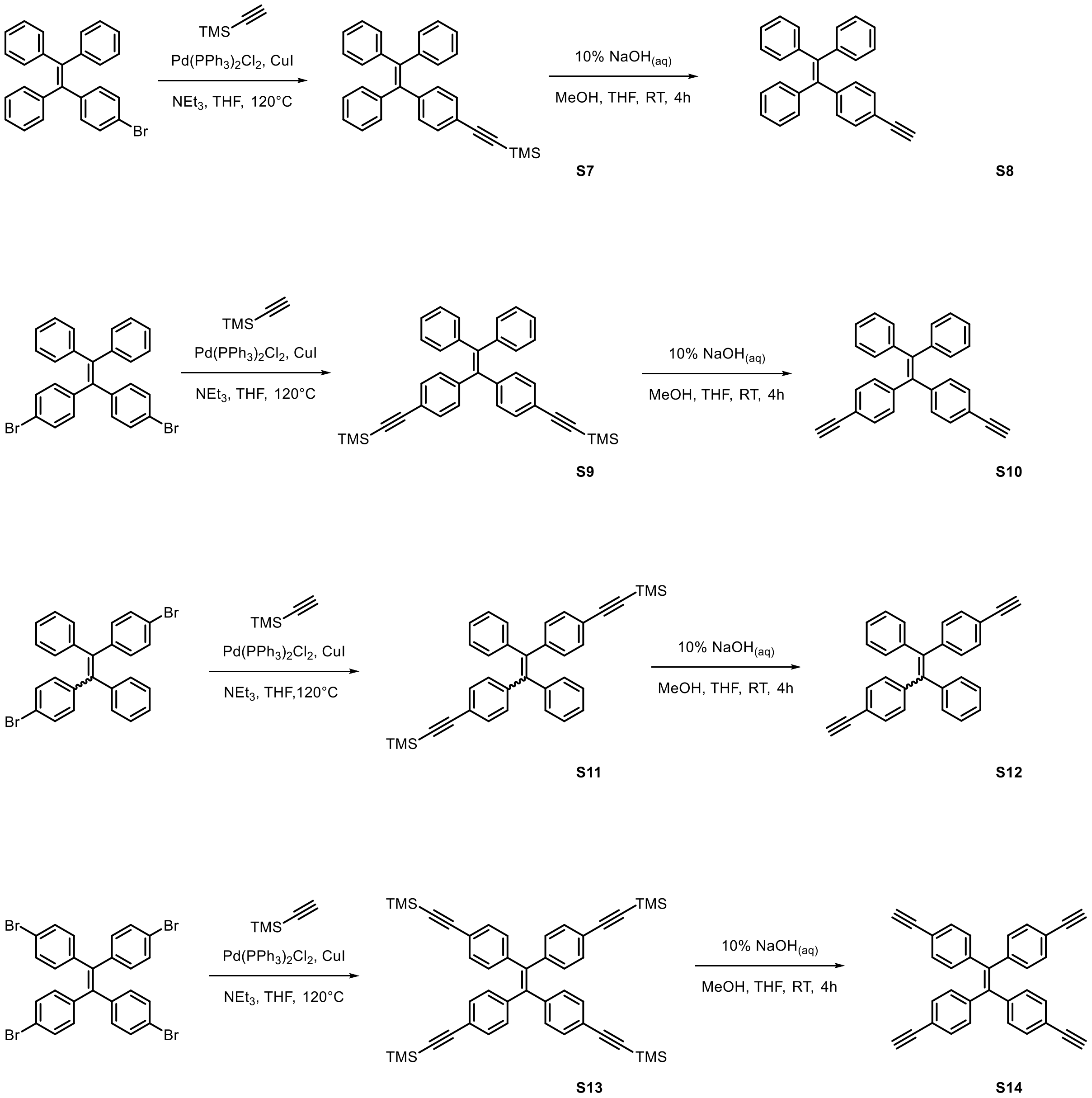

Scheme S2. Synthesis of alkyne functionalised tetraphenyletheylene derivatives. **S7, S9, S11** and **S13** were synthesised according to General Procedure 5. **S8, S10, S12** and **S14** were synthesised according to General Procedure 6.[8]

General Procedure 5
The appropriate bromo functionalised TPE (3.64 mmol), CuI (69 mg, 0.364 mmol) and $Pd(PPh_3)_2Cl_2$ (256 mg, 0.364 mmol) were added to a microwave vial, sealed and degassed under a constant stream of nitrogen for 15 minutes, after which the THF (8 ml) and NEt_3 (8 ml) and were subjected to stirring and sonication for 10 minutes. After which TMS acetylene (2.5 equivalents per aryl bromide) was added and the vial was heated to 120°C under microwave irradiation for 1 hour, at which time additional TMS acetylene (2.5 equivalents per aryl bromide) was added, the mixture sonicated and subjected to the same temperature for 1 hour. Once cooled, the microwave vial was opened diluted with CH_2Cl_2 (ca. 150 ml) and washed with EDTA/$NH_4OH_{(aq)}$ solution (20 ml) and water (50 ml), the collected organic phase was dried over $MgSO_4$ and concentrated to dryness and purified by 'flash' silica gel column chromatography (hexane) to afford the TMS protected alkynes as white solids.

General Procedure 6
The appropriate TMS protected alkyne precursor (2.00 mmol) was dissolved in the minimum amount of THF (ca. 15 ml) to which was added an equal volume of methanol to this mixture 10% $NaOH_{(aq)}$ (10 ml) was added and left to stir under ambient conditions until complete as determined by TLC (ca. 4 hours). The mixture was then diluted with CH_2Cl_2 (150 ml) and the organic phase washed with water (3 × 50 ml) and dried over $MgSO_4$, the solvent was removed *in vacuo* to afford the alkyne as a white solid.

General Procedure 7
The appropriate alkyne precursor (0.40 mmol) was dissolved in anhydrous THF (20 ml) and cooled to -78°C. To this solution a 1.6 M nBuLi solution (1.5 equivalents per alkyne) was added dropwise and left to stir for 30 minutes after at this temperature. After which a THF solution (10 ml) of I_2 (2 equivalents per alkyne) was added dropwise, the temperature was maintained at -78°C for 10 minutes, the cooling bath removed and allowed to warm to room temperature over the course of 30 minutes. The reaction mixture was then diluted with CH_2Cl_2 (150 ml), washed with $Na_2S_2O_{3(aq)}$ solution until decolourised and subsequently with water (2 × 50 ml), the collected organic phase was dried over $MgSO_4$ and solvent removed *in vacuo*. The crude residue was dry loaded onto silica gel and extracted with CH_2Cl_2:hexane (1:9 v/v) to afford iodoalkyne precursor as a yellow solid which was used immediately for the subsequent CuAAC reaction.

General Procedure 8
$[Cu(MeCN)_4]PF_6$ (0.1 equivalents per iodo/proto-alkyne) and TBTA (0.1 equivalents per iodo/proto-alkyne) were dissolved in the minimum amount anhydrous degassed CH_2Cl_2 (ca. 5 ml) and left to stir for 15 minutes. After which time the appropriate iodoalkyne was added to the solution as a solid, followed by the appropriate phenyl azide (1.1 equivalents per iodo/proto-alkyne). Once complete, as determined by TLC analysis, the reaction mixture was diluted with CH_2Cl_2 (ca. 150 ml) and washed with EDTA/$NH_4OH_{(aq)}$ solution (20 ml) and water (50 ml), the collected organic phase was dried over $MgSO_4$ and concentrated to dryness and purified by 'flash' silica gel column chromatography the target products as yellow solids.

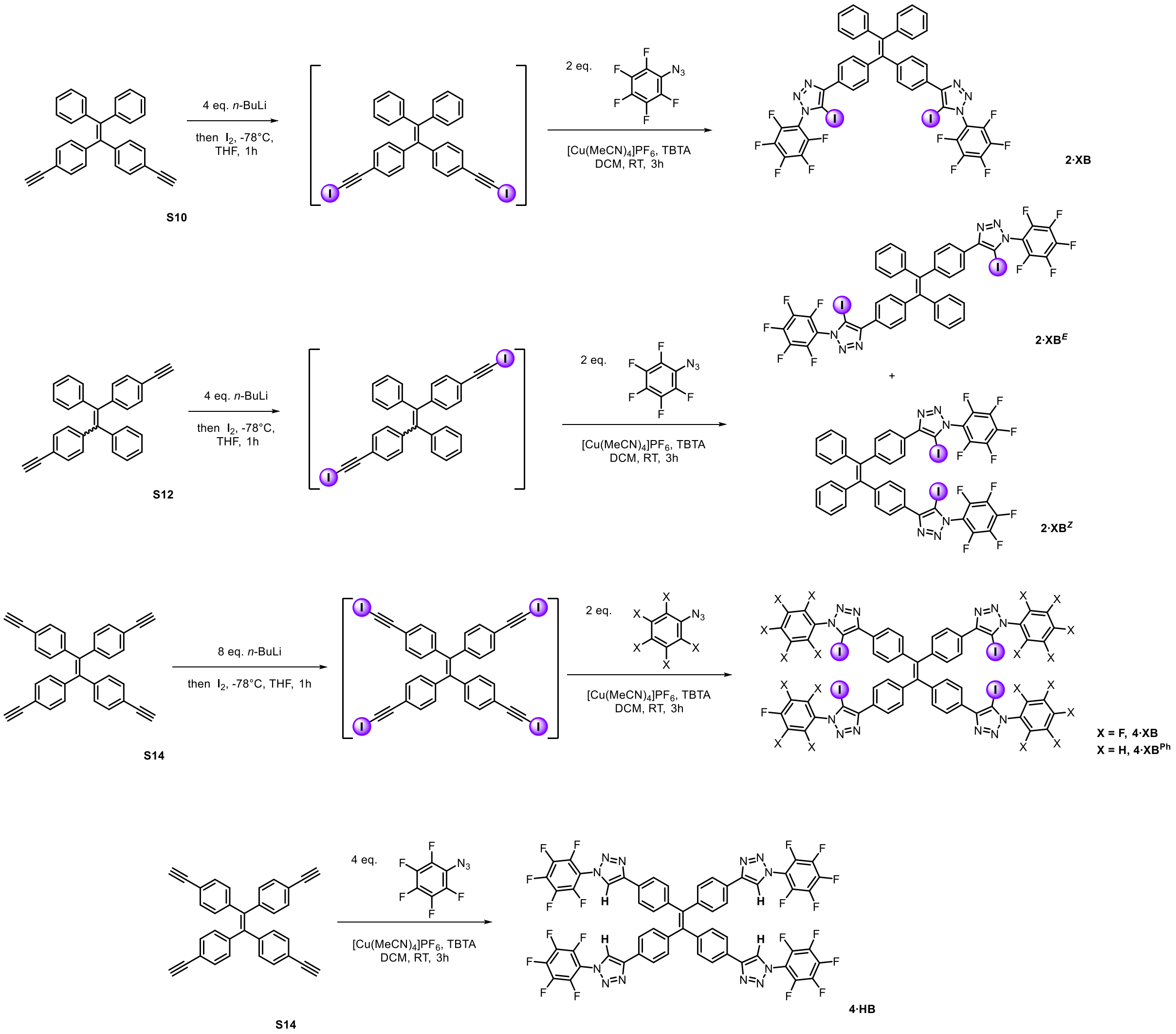

Scheme S3. Synthesis of XB and HB tetraphenyletheylene receptor systems.

1·XB, 2·XB, 2·XB*^Z^*, 2·XB*^E^*, **4·XB** and **4·XB^{Ph}** were synthesised according to General Procedures 7 and 8.

4·HB was synthesised according to General Procedure 8.

1·XB

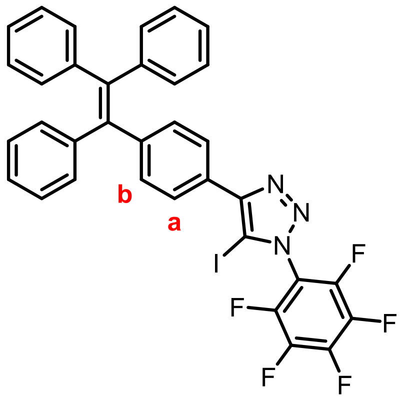

Isolated as yellow solid (83%).

^{1}H NMR (400 MHz, $CDCl_3$) δ 7.82 (d, J = 7.3 Hz, $2H_a$), 7.18 (d, J = 7.3 Hz, $2H_b$), 7.15 – 7.01 (m, 15H).

^{19}F NMR (377 MHz, $CDCl_3$) δ -142.16 – -142.56 (m), -147.45 (tt, J = 21.4, 3.4 Hz), -158.80 – -159.56 (m).

^{13}C NMR (126 MHz, $CDCl_3$) δ 150.30, 145.03, 143.77 (dm, J = 260 Hz), 143.66, 143.57, 143.56, 141.98, 140.32, 138.10 (dm, J = 252 Hz), 131.88, 131.55, 131.49, 131.46, 127.99, 127.92, 127.83, 127.03, 126.87, 126.76, 126.64, 112.55 (m), 79.75.

HRMS (ESI+ve) m/z: 692.0612 ($[M+H]^+$, $C_{34}H_{20}F_5IN_3$ requires 692.0617).

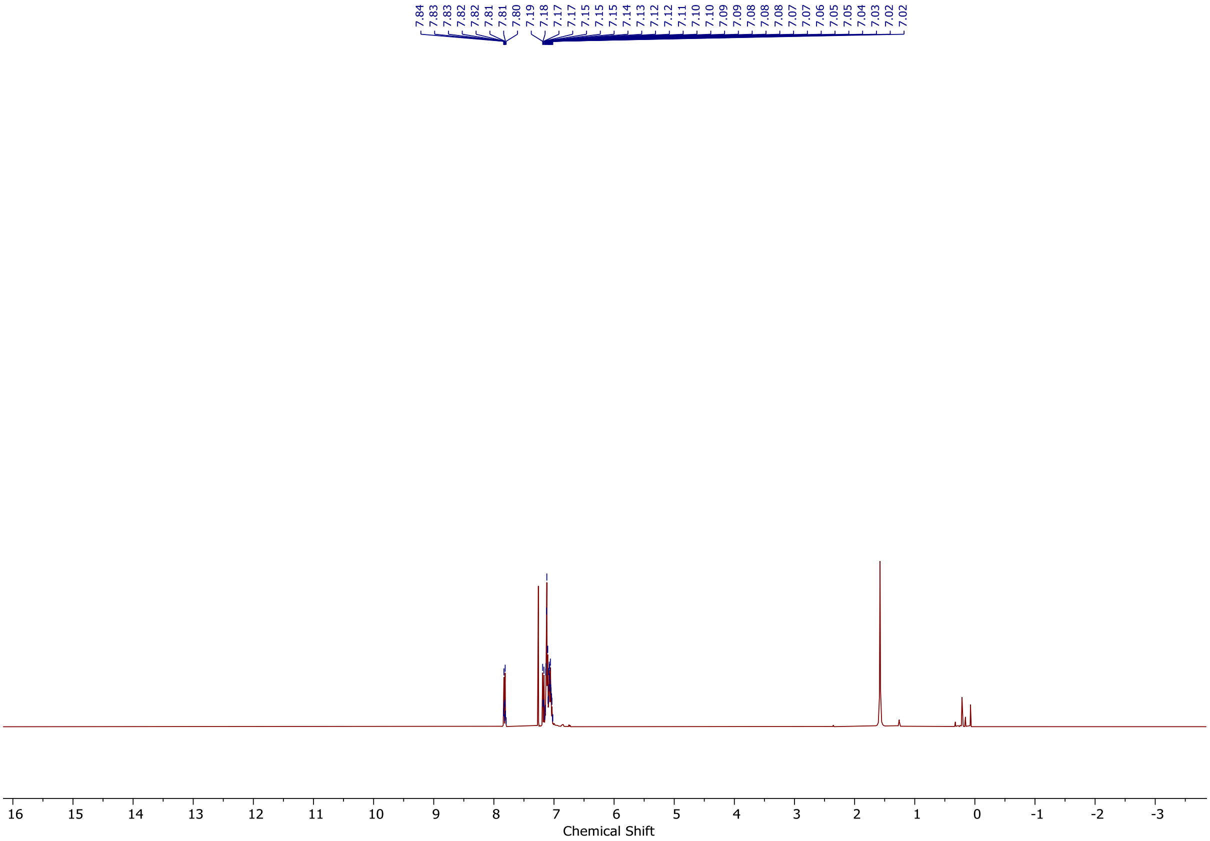

Figure S1. ^{1}H NMR Spectrum of **1·XB** ($CDCl_3$, 400 MHz, 298K).

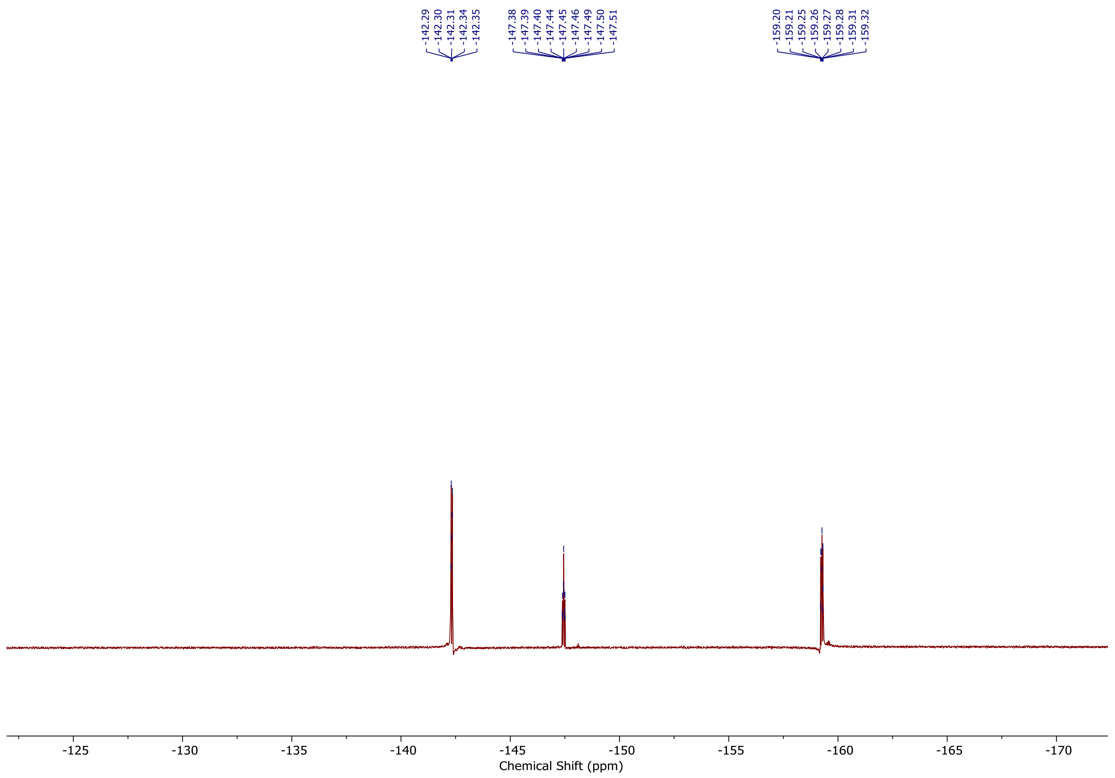

Figure S2. ^{19}F NMR Spectrum of **1·XB** ($CDCl_3$, 377 MHz, 298K).

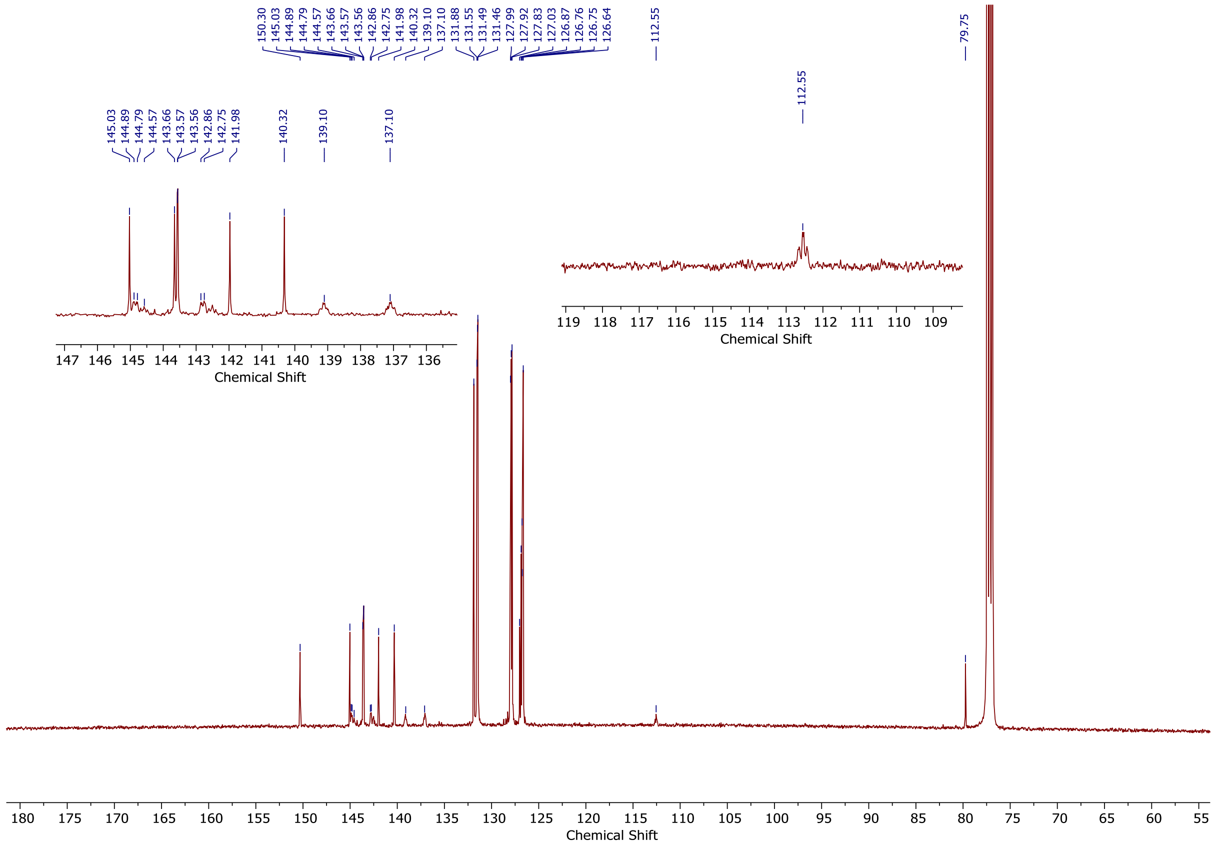

Figure S3. ^{13}C NMR Spectrum of **1·XB** ($CDCl_3$, 126 MHz, 298K).

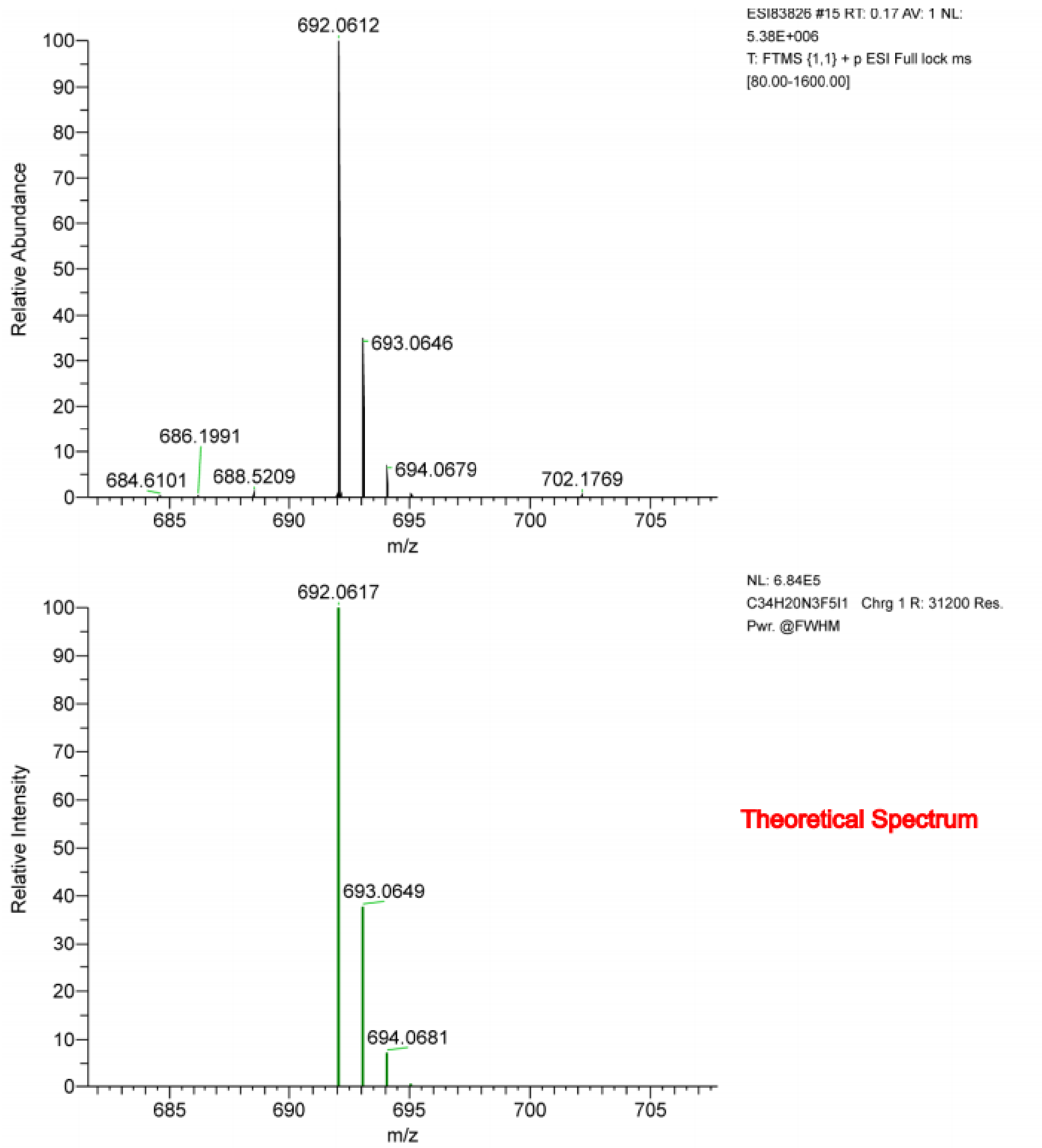

Figure S4. HRESI spectrum of **1·XB**.

2·XB

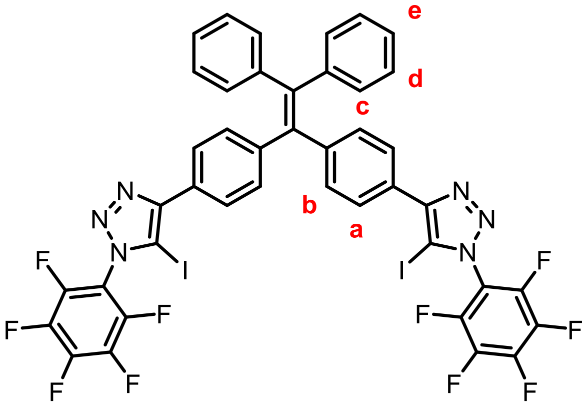

Isolated as yellow solid (67%).

¹H NMR (500 MHz, $CDCl_3$) δ 7.85 (d, J = 8.62 Hz, $4H_a$), 7.22 (d, J = 8.62 Hz, $4H_a$), 7.18 – 7.07 (m, $10H_{c\text{-}e}$).

¹⁹F NMR (470 MHz, $CDCl_3$) δ -141.85 – -142.66 (m), -147.40, -158.75 – -159.59 (m).

¹³C NMR (126 MHz, $CDCl_3$) δ 150.23, 144.69, 143.80 (dm, J = 265 Hz), 143.54 (dm, J = 265 Hz), 143.38, 142.84, 139.55, 138.11 (dm, J = 261 Hz), 131.96, 131.48, 128.04, 127.28, 127.05, 126.76, 112.53 (m), 79.85.

HRMS (ESI+ve) m/z: 1050.9592 ($[M+H]^+$, $C_{42}H_{19}F_{10}I_2N_6$ requires 1050.9595).

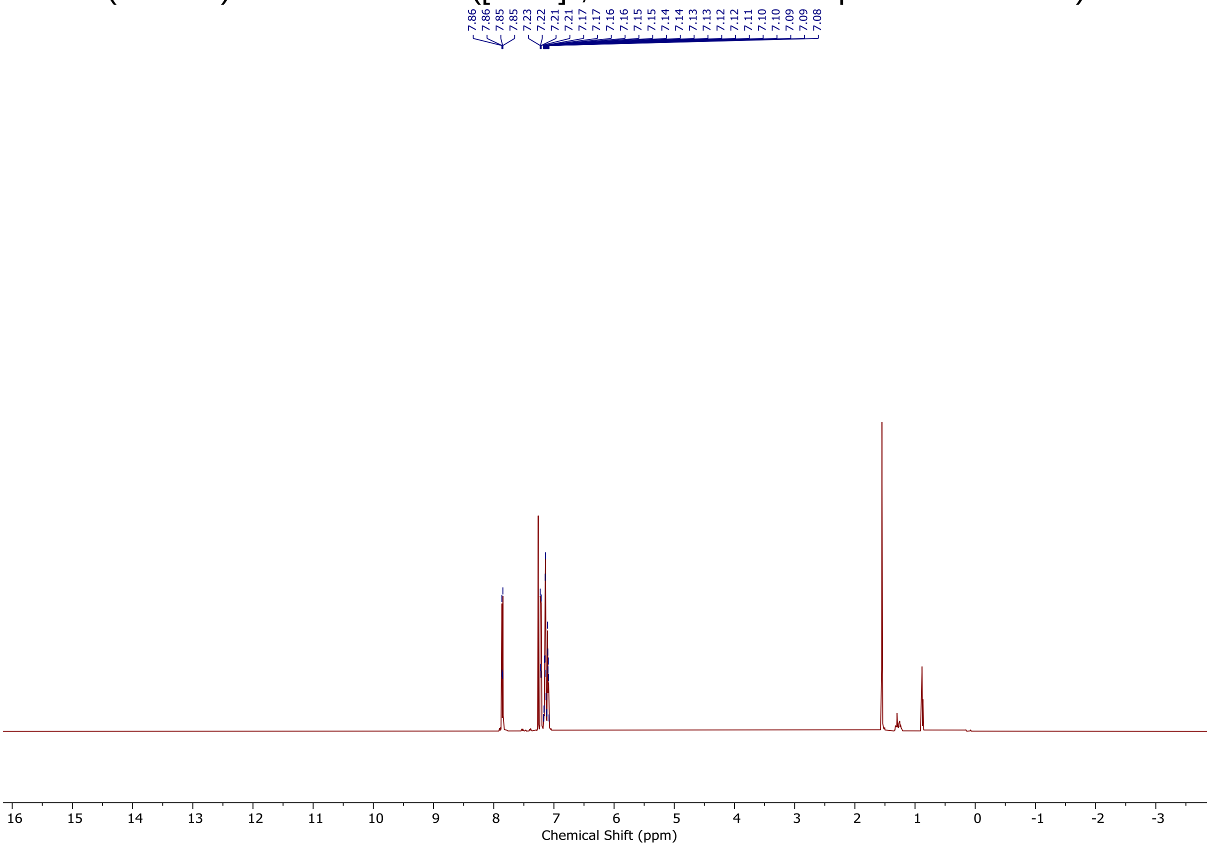

Figure S5. ¹H NMR spectrum of **2·XB** ($CDCl_3$, 400 MHz, 298K).

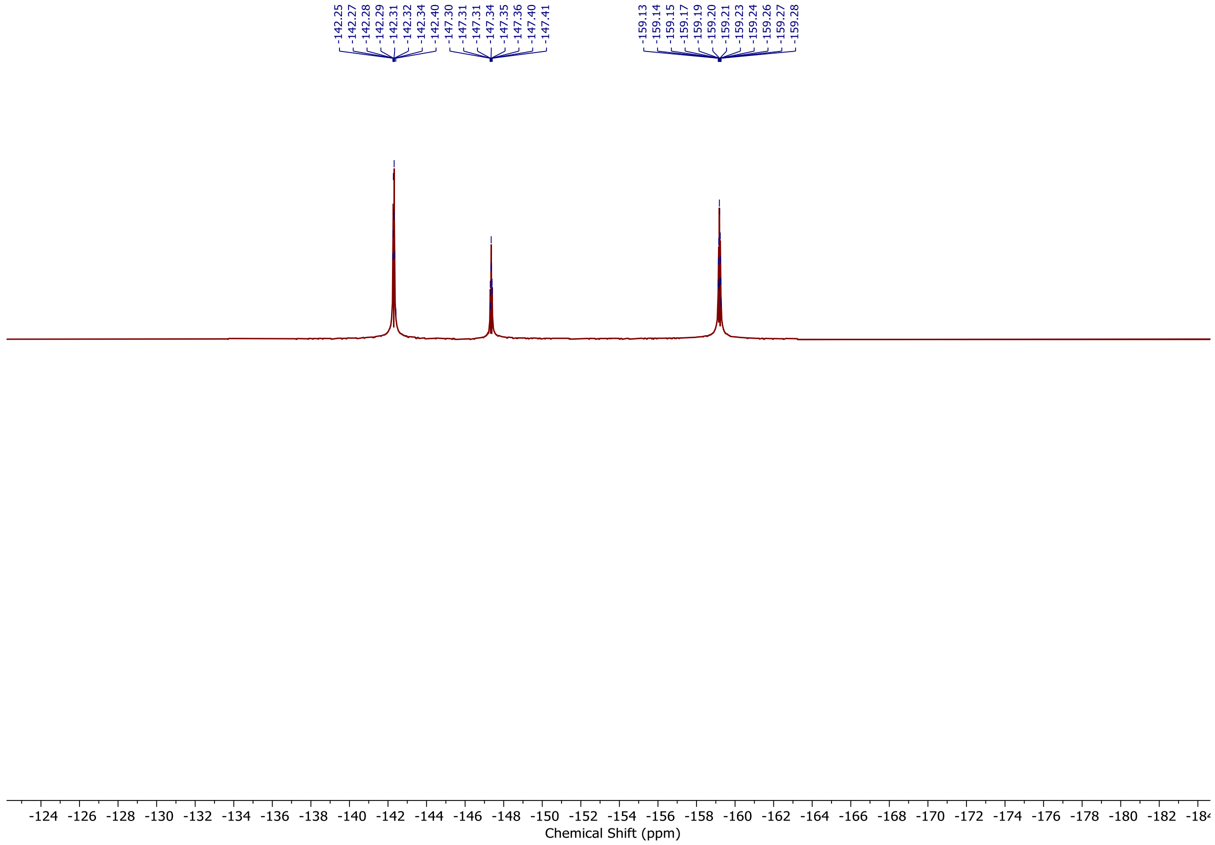

Figure S6. ^{19}F NMR spectrum of **2·XB** ($CDCl_3$, 377 MHz, 298K).

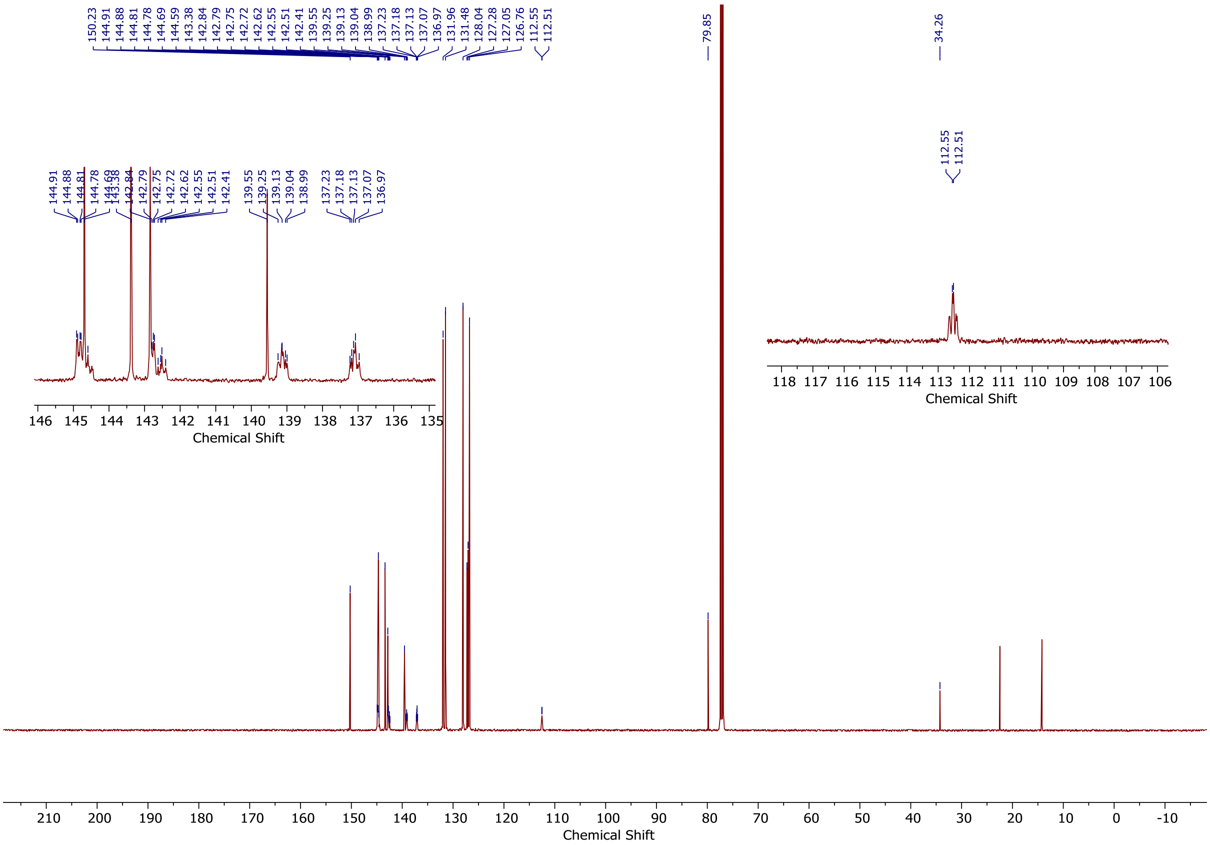

Figure S7. ^{13}C NMR spectrum of **2·XB** ($CDCl_3$, 126 MHz, 298K).

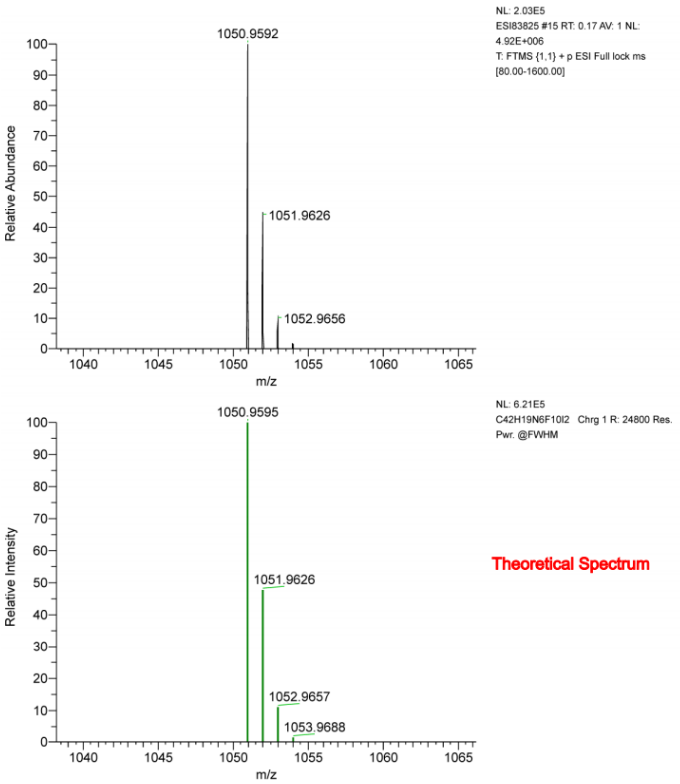

Figure S8. HRESI spectrum of **2·XB**.

4·XB

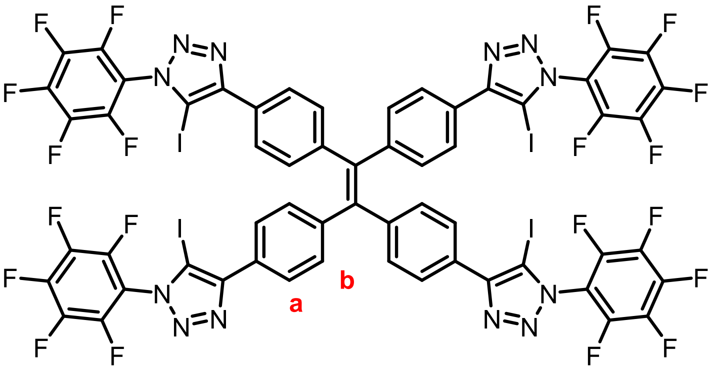

Isolated as yellow solid (65%).

^{1}H NMR (400 MHz, $CDCl_3$) δ 7.89 (d, J = 8.62 Hz, 8H_a), 7.29 (d, J = 8.62 Hz, 8H_b).

^{19}F NMR (377 MHz, $CDCl_3$) δ -141.96 – -142.63 (m), -147.09 – -147.69 (m), -158.90 – -159.51 (m).

^{13}C NMR (126 MHz, $CDCl_3$) δ 150.29, 144.11, 143.81 (dm, J = 263 Hz), 143.56 (dm, J = 257 Hz) 141.37, 138.09 (dm, J = 259 Hz), 132.00, 127.86, 127.17, 112.51 (m), 80.42.

HRMS (ESI+ve) m/z: 1768.7538 ($[M+H]^+$, $C_{58}H_{17}F_{20}I_4N_{12}$ requires 1768.7553).

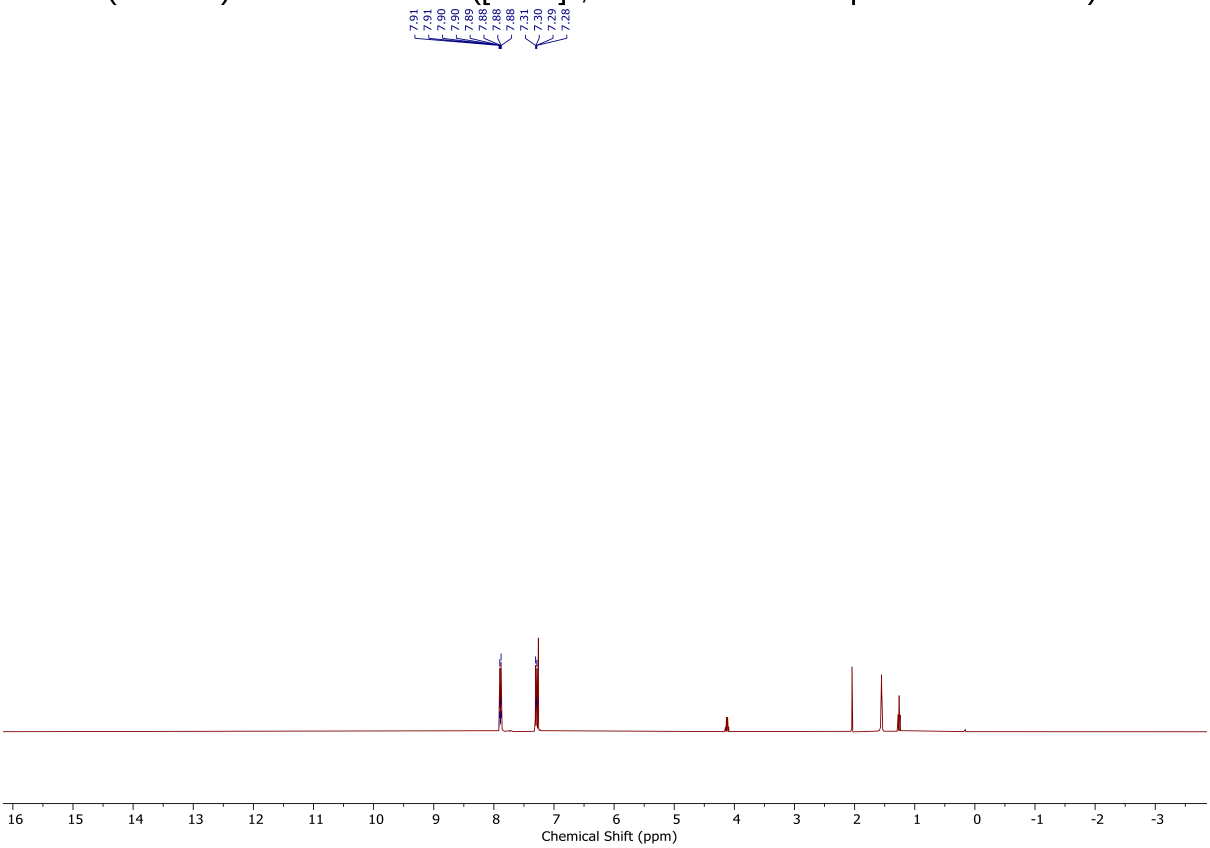

Figure S9. ^{1}H NMR spectrum of **4·XB** ($CDCl_3$, 400 MHz, 298K)..

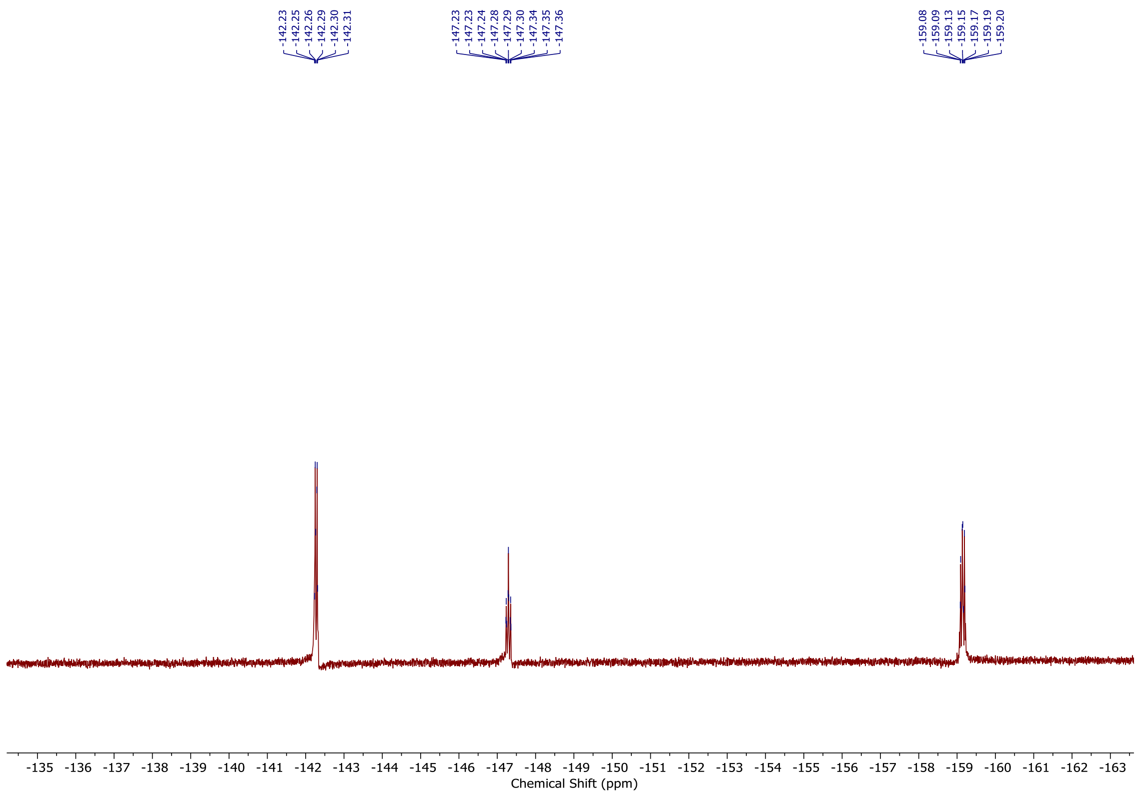

Figure S10. ^{19}F NMR spectrum of **4·XB** ($CDCl_3$, 377 MHz, 298K).

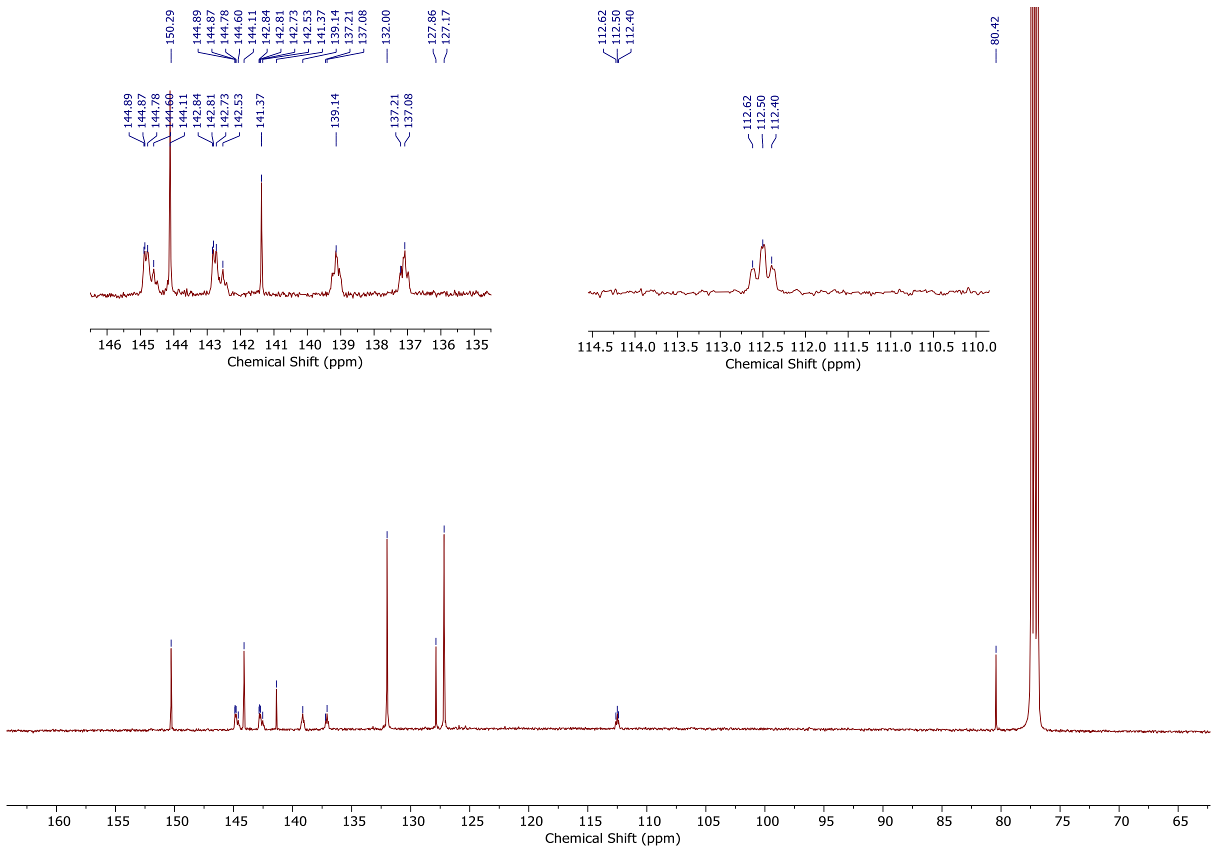

Figure S11. ^{13}C NMR spectrum of **4·XB** ($CDCl_3$, 126 MHz, 298K).

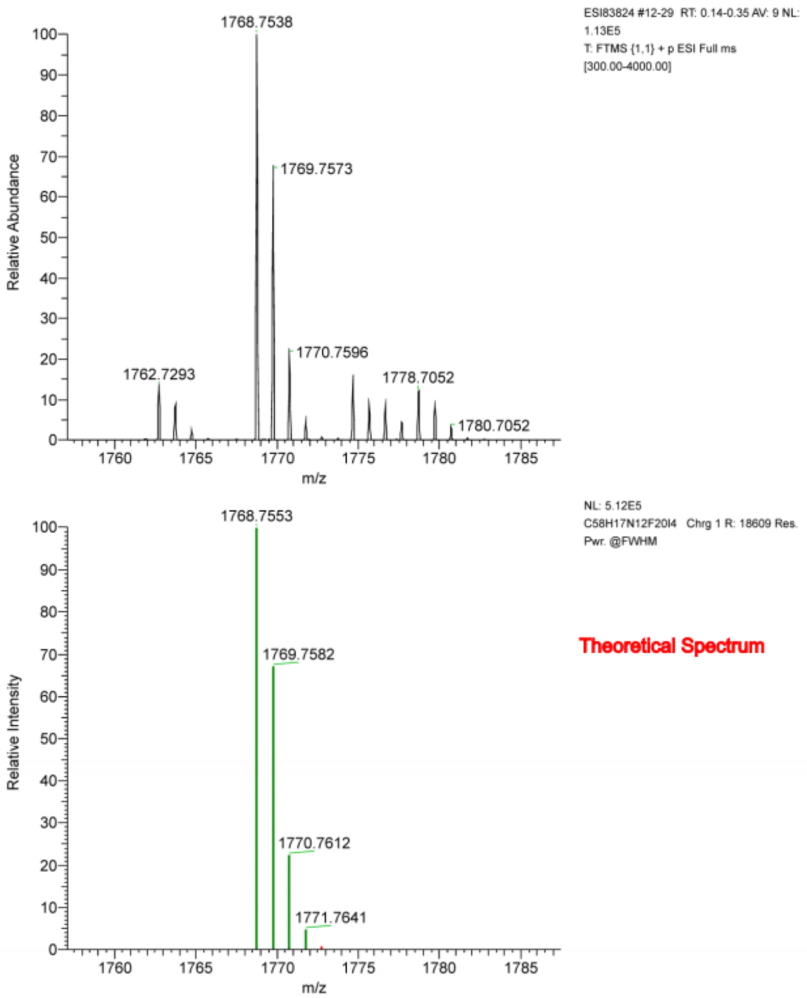

Figure S12. HRESI spectrum of **4·XB**.

4·HB

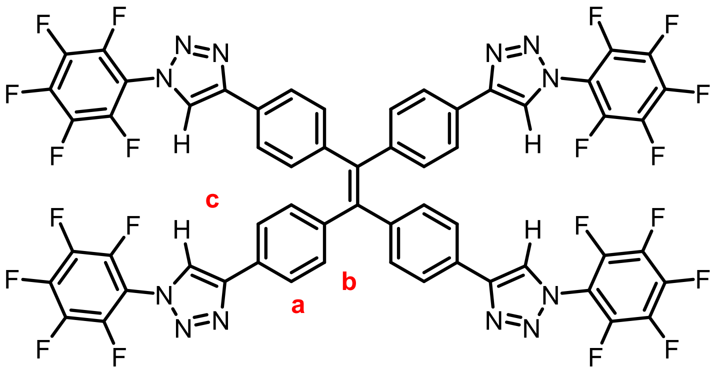

Isolated as yellow solid (32%).

^{1}H NMR (500 MHz, DMSO) δ 9.08 (s, $4H_c$), 7.84 – 7.78 (m, $8H_a$), 7.27 – 7.17 (m, $8H_b$).

^{19}F NMR (377 MHz, DMSO) δ -146.70 – -147.66 (m), -150.90 – -151.67 (m), -160.50 – -161.21 (m).

^{13}C NMR (126 MHz, DMSO) δ 146.69, 143.25, 141.93 (dm, J = 247 Hz), 140.63, 140.47, 137.67 (dm, J = 252 Hz), 131.68, 128.67, 127.81, 125.29, 124.91, 124.42, 112.47.

HRMS (ESI+ve) m/z: 1265.1687 ($[M+H]^+$, $C_{58}H_{21}F_{20}N_{12}$ requires 1265.1673).

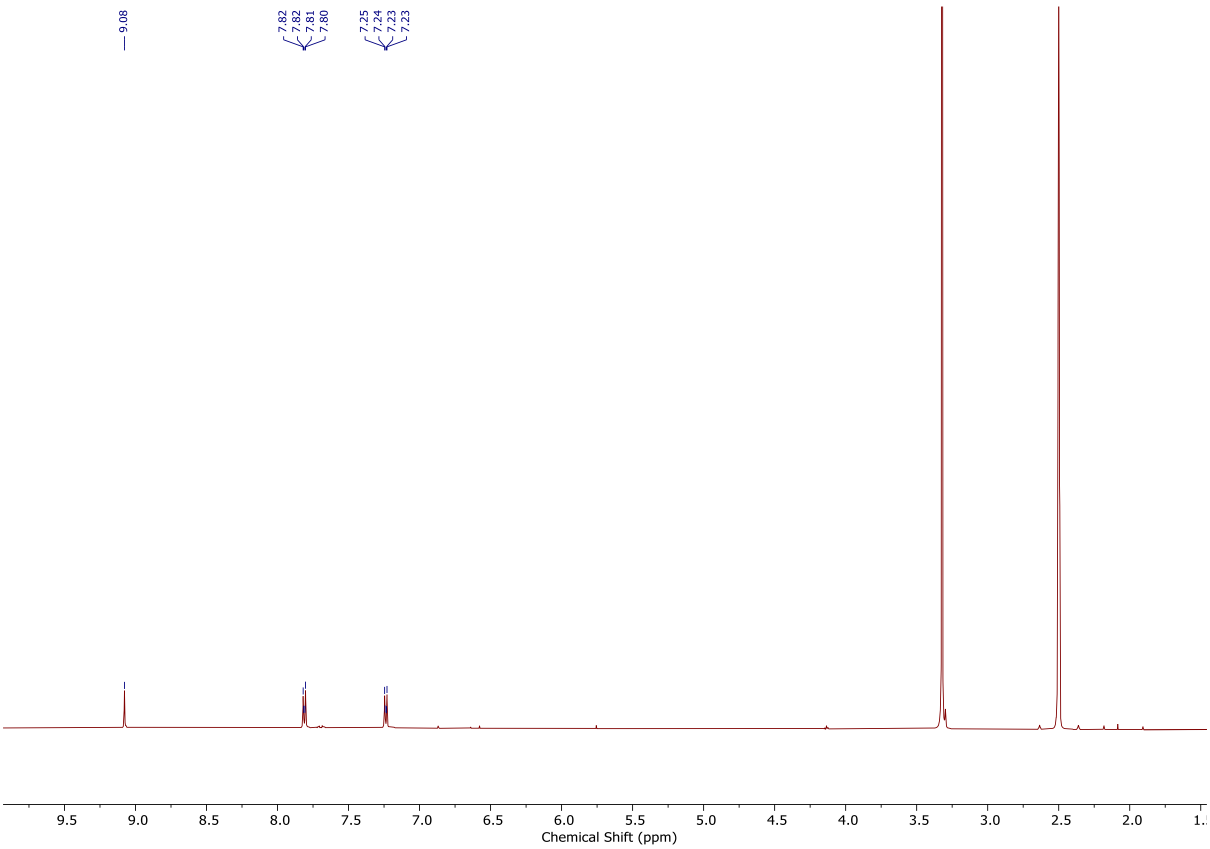

Figure S13. ^{1}H NMR spectrum of **4·HB** (DMSO, 500 MHz, 298K).

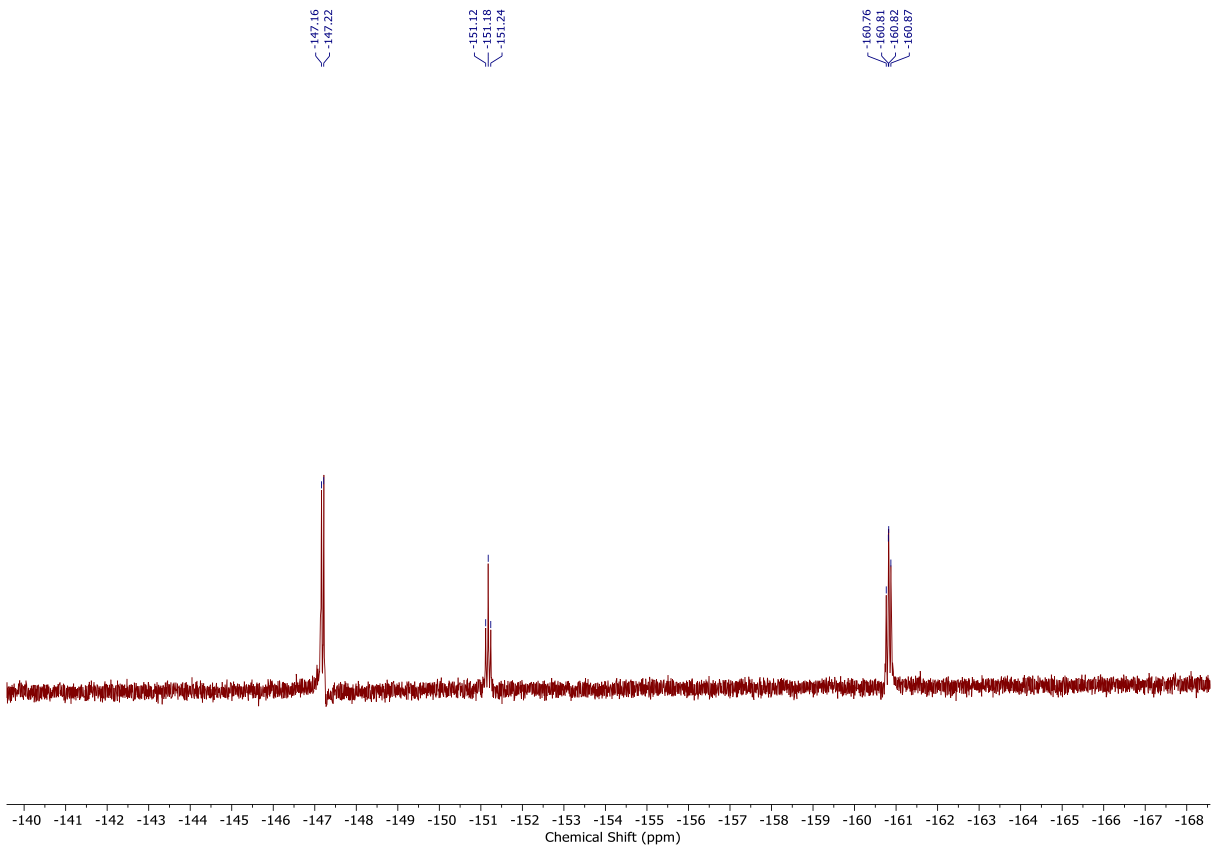

Figure S14. ^{19}F NMR spectrum of **4·HB** (DMSO, 377 MHz, 298K).

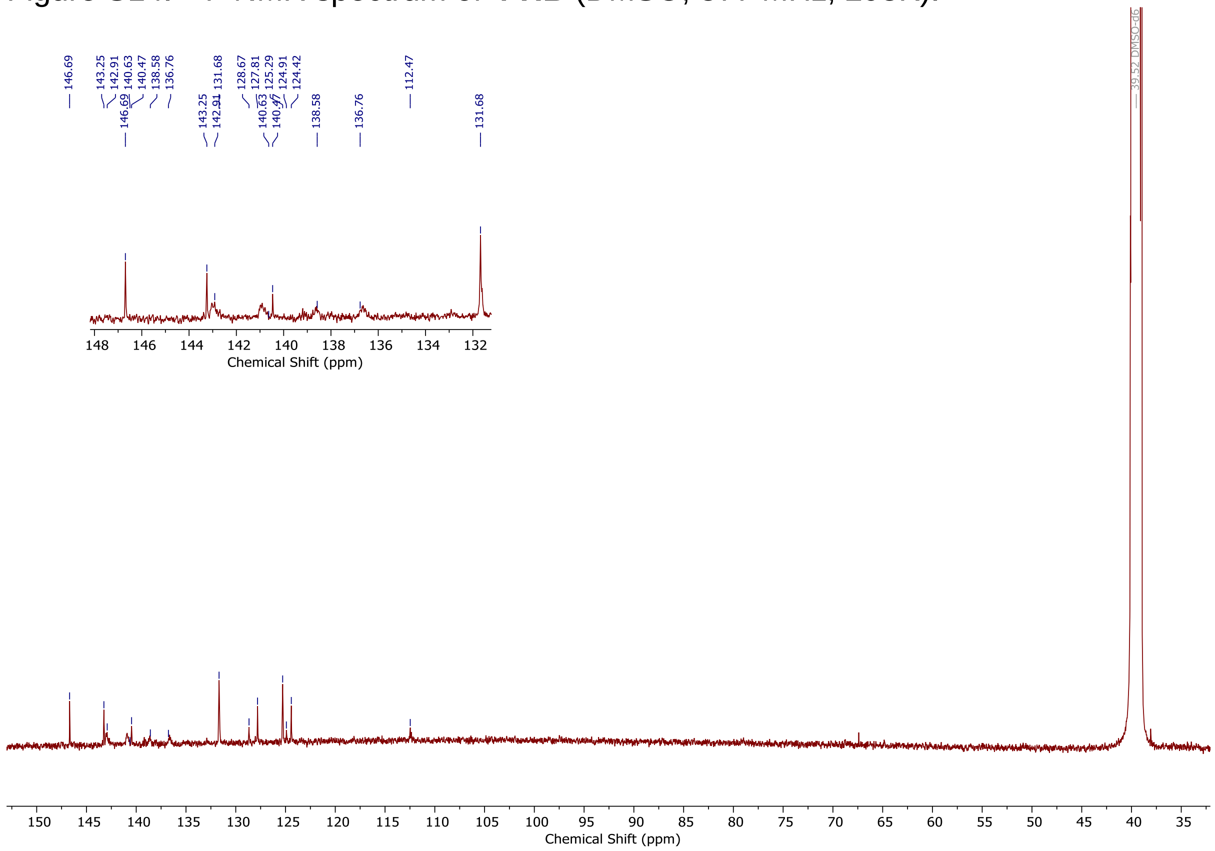

Figure S15. ^{13}C NMR spectrum of **4·HB** (DMSO, 126 MHz, 298K).

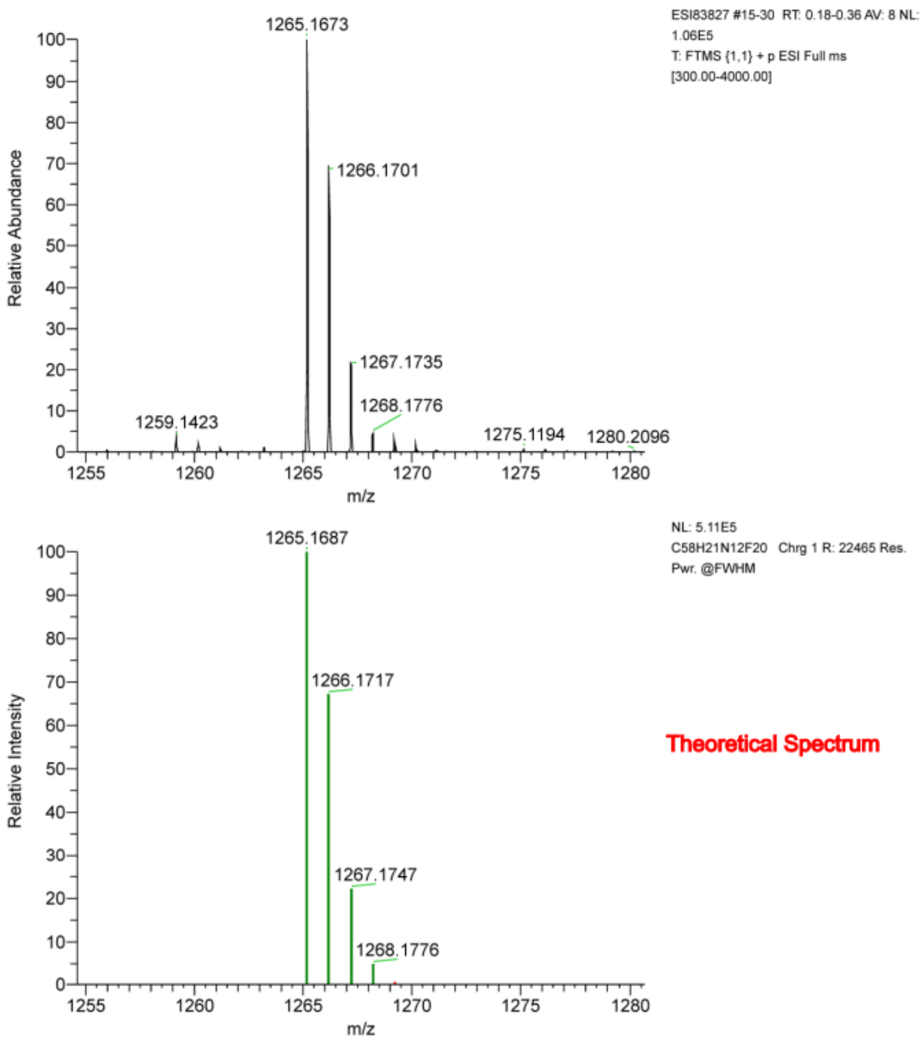

Figure S16. HRESI spectrum of **4·HB**.

2·XB*E*

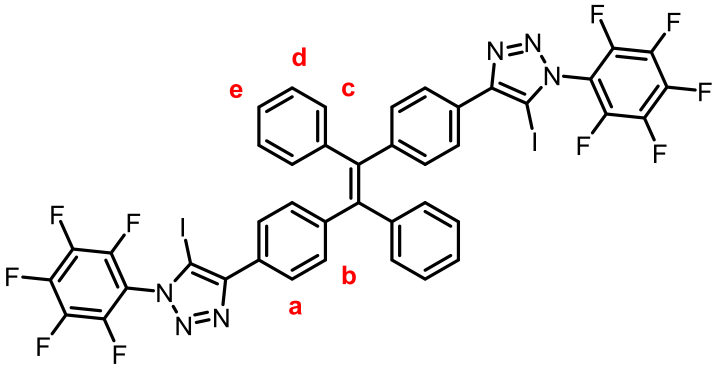

Isolated as yellow solid (34%).

^{1}H NMR (500 MHz, $CDCl_3$) δ 7.83 (d, *J* = 8.1 Hz, $4H_a$), 7.22 (d, *J* = 8.1 Hz, $4H_b$), 7.19 – 7.05 (m, $10H_{c,d,e}$).
^{19}F NMR (470 MHz, $CDCl_3$) δ -142.34, -147.37 (t, J = 22.3 Hz), -158.78 – -159.61 (m).
^{13}C NMR (126 MHz, $CDCl_3$) δ 150.42, 144.78, 143.62 (dm, *J* = 236 Hz), 143.23, 141.27, 139.13, 138.12 (dm, *J* = 262 Hz), 137.08, 131.92, 131.54, 127.99, 127.40, 127.00, 112.53, 80.31.
HRMS (ESI+ve) m/z: 1050.9586 ($[M+H]^+$, $C_{42}H_{19}F_{10}N_6$ requires 1050.9595).

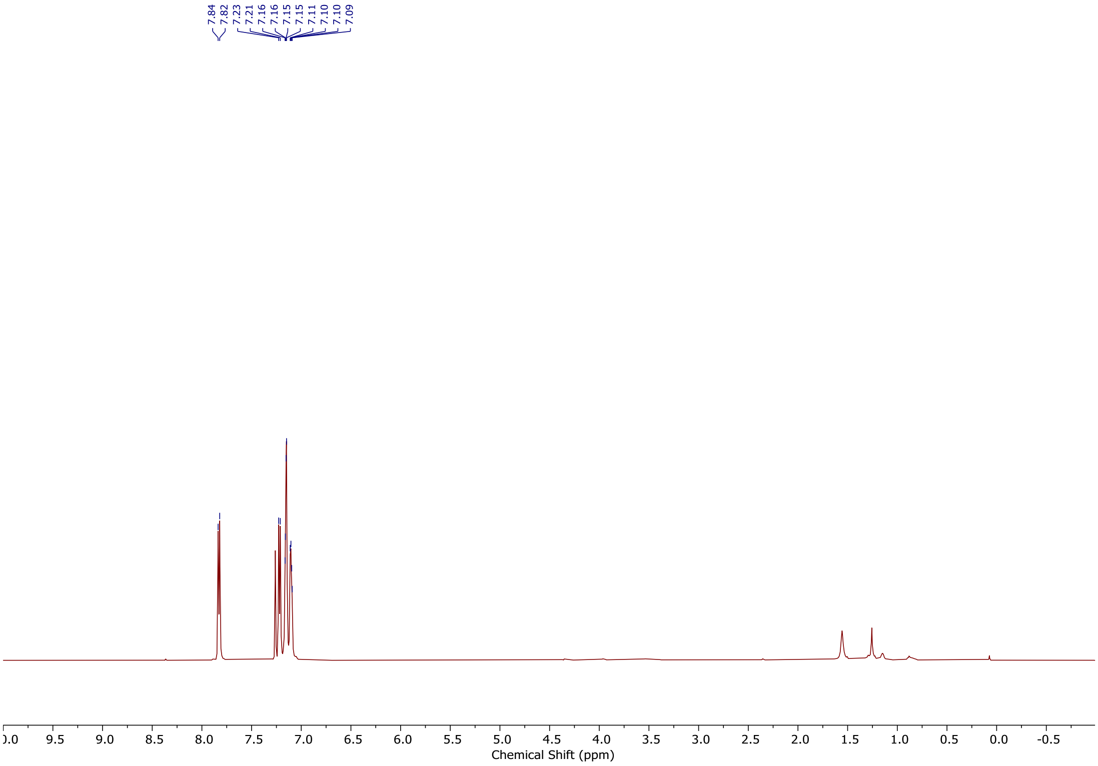

Figure S17. ^{1}H NMR spectrum of **2·XB*E*** ($CDCl_3$, 500 MHz, 298K).

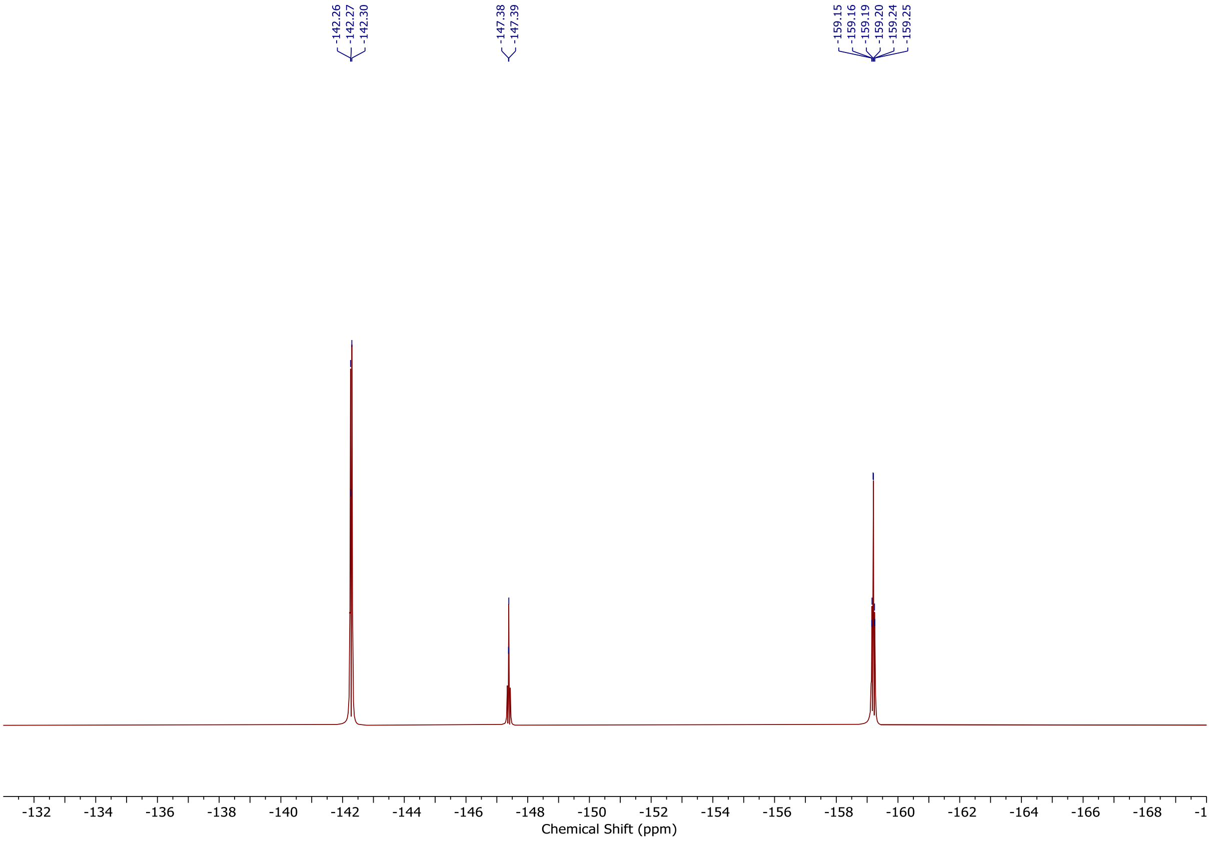

Figure S18. ^{19}F NMR spectrum of **2·XB*E*** ($CDCl_3$, 470 MHz, 298K).

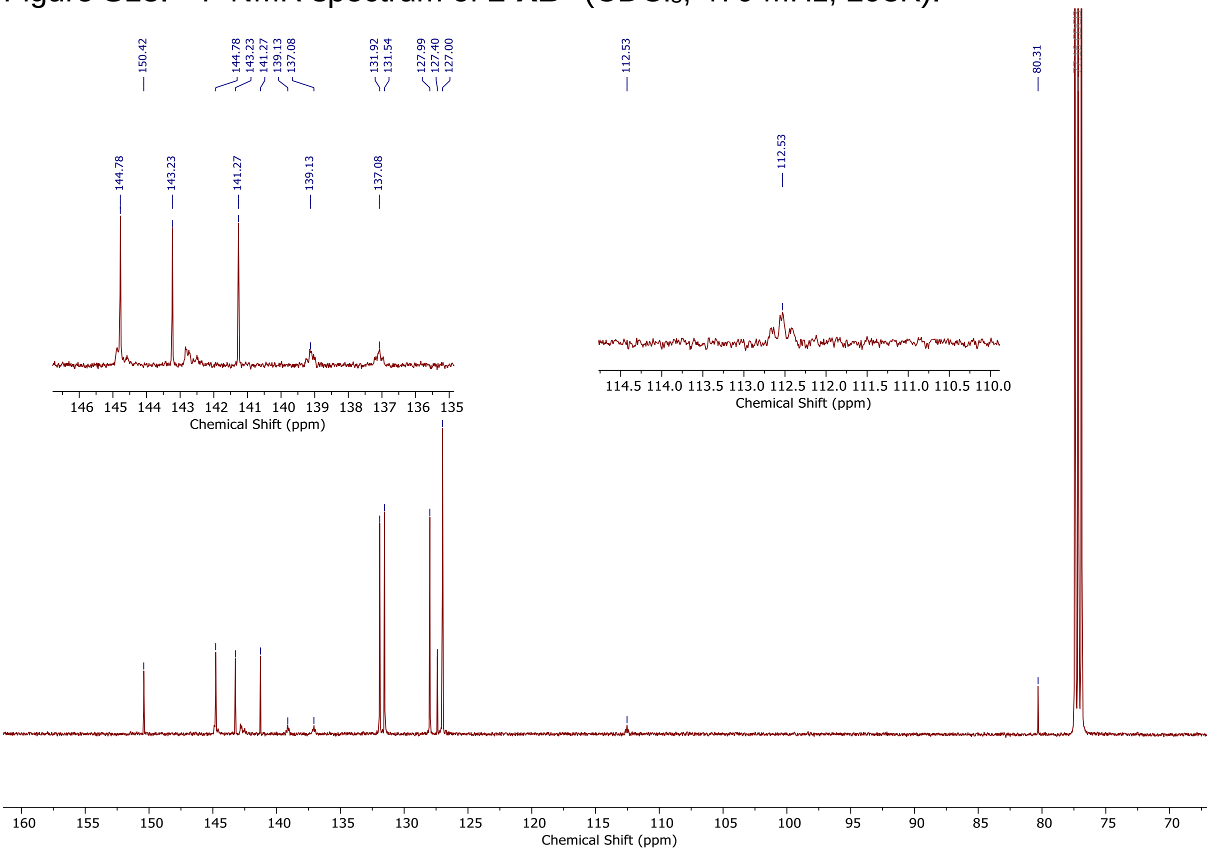

Figure S19. ^{13}C NMR spectrum of **2·XB*E*** ($CDCl_3$, 126 MHz, 298K).

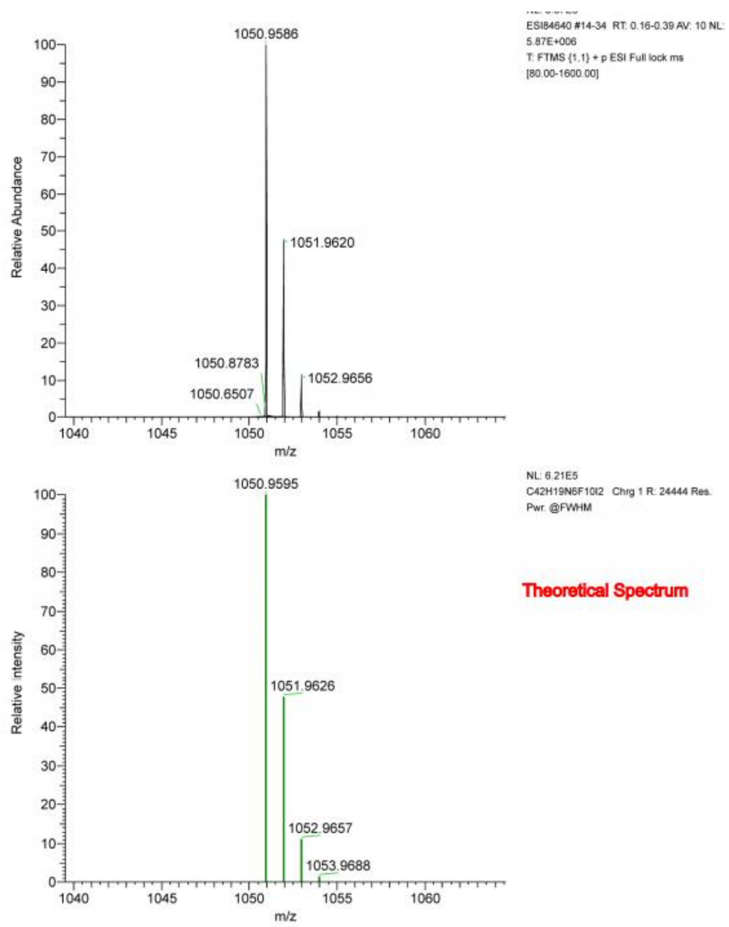

Figure S20. HRESI of **2·XB**^{E}.

2·XB^{Z}

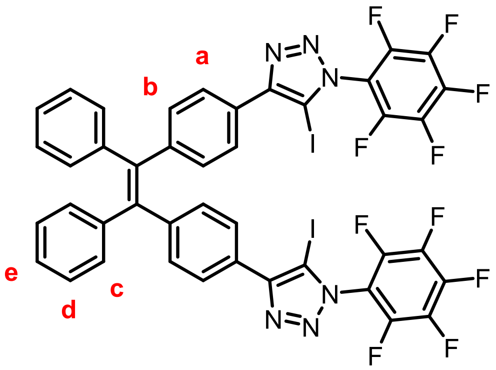

Isolated as yellow solid (38%).

^{1}H NMR (400 MHz, $CDCl_3$) δ 7.84 (d, J = 8.4 Hz, 4H_a), 7.22 – 7.05 (m, 14$H_{b,c,d,e}$).
^{19}F NMR (470 MHz, $CDCl_3$) δ -141.71 – -142.64 (m), -146.79 – -147.81 (m), -158.51 – -159.52 (m).
^{13}C NMR (126 MHz, $CDCl_3$) δ 150.25, 144.80, 143.72 (dm, J = 281 Hz) 143.28, 141.19, 138.13 (dm, J = 261 Hz), 135.92, 131.88, 131.58, 128.13, 127.24, 127.09, 126.68, 125.67, 79.79.
HRMS (ESI+ve) m/z: 1050.9581 ($[M+H]^+$, $C_{42}H_{19}F_{10}N_6$ requires 1050.9595).

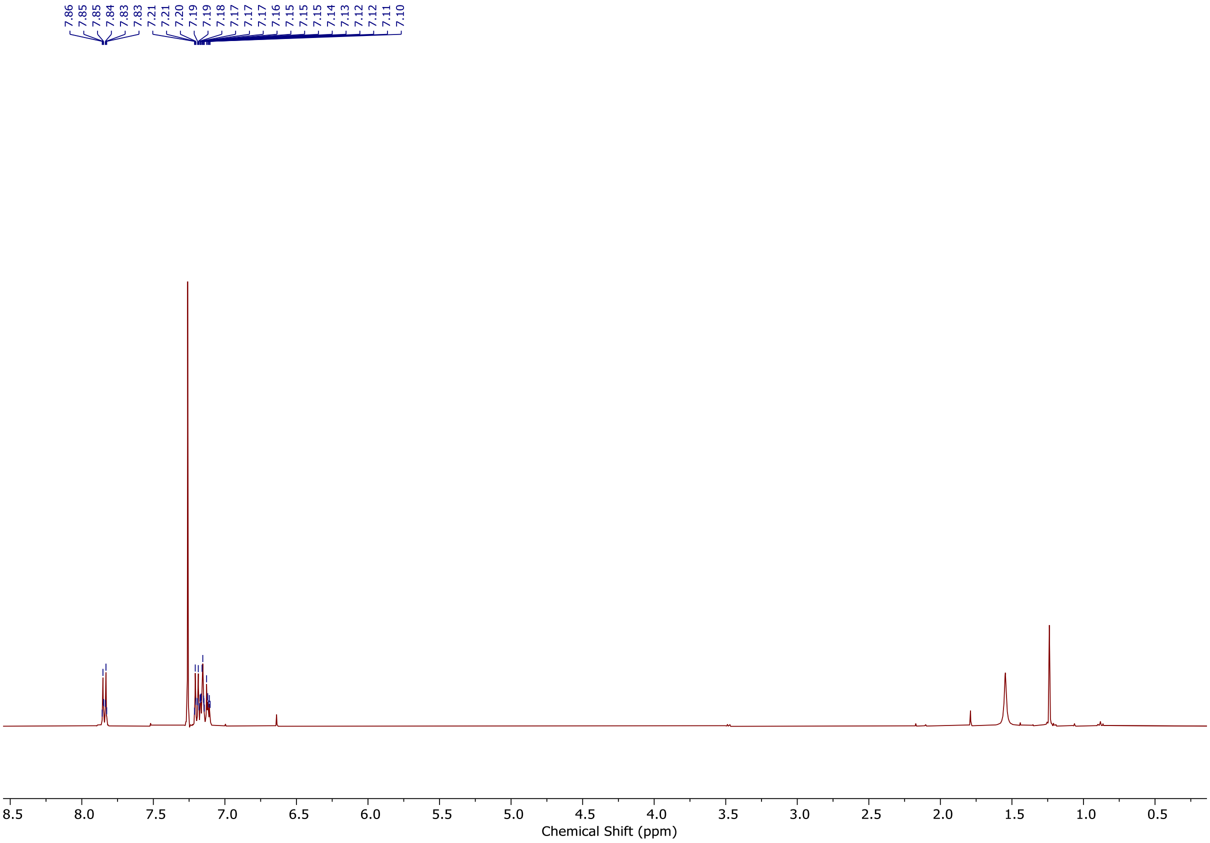

Figure S21. ^{1}H NMR spectrum of **2·XB^{Z}** ($CDCl_3$, 400 MHz, 298K).

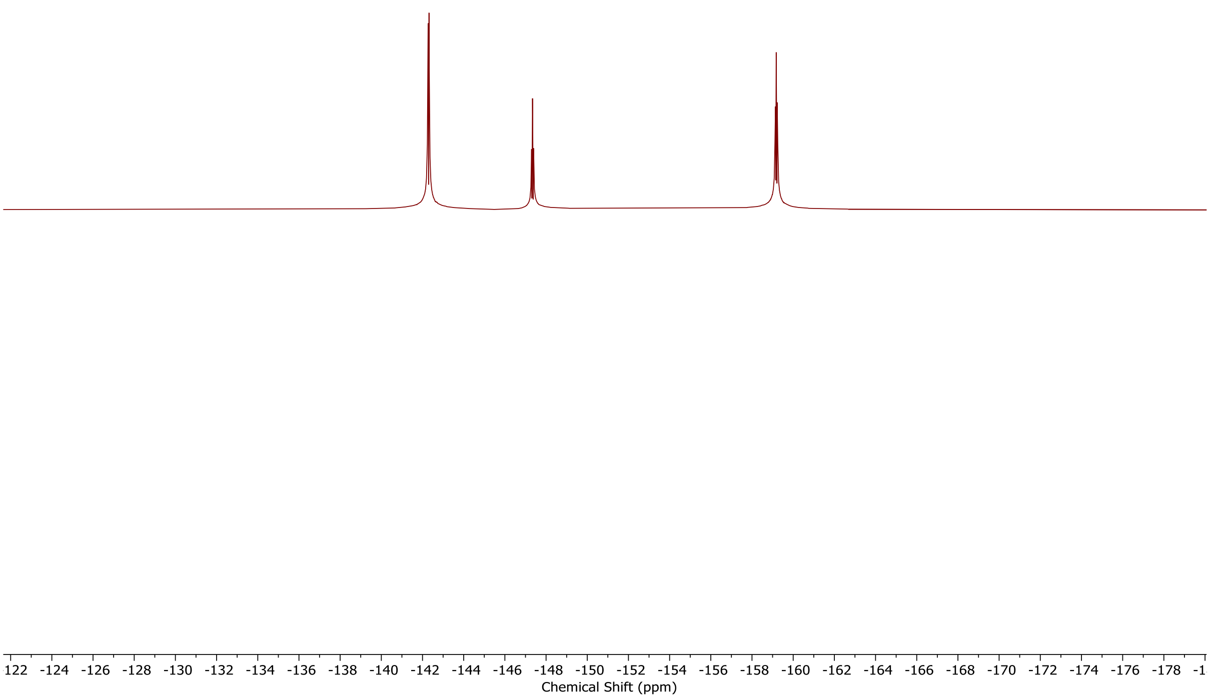

Figure S22. ^{19}F NMR spectrum of **2·XB**^{Z} ($CDCl_3$, 470 MHz, 298K).

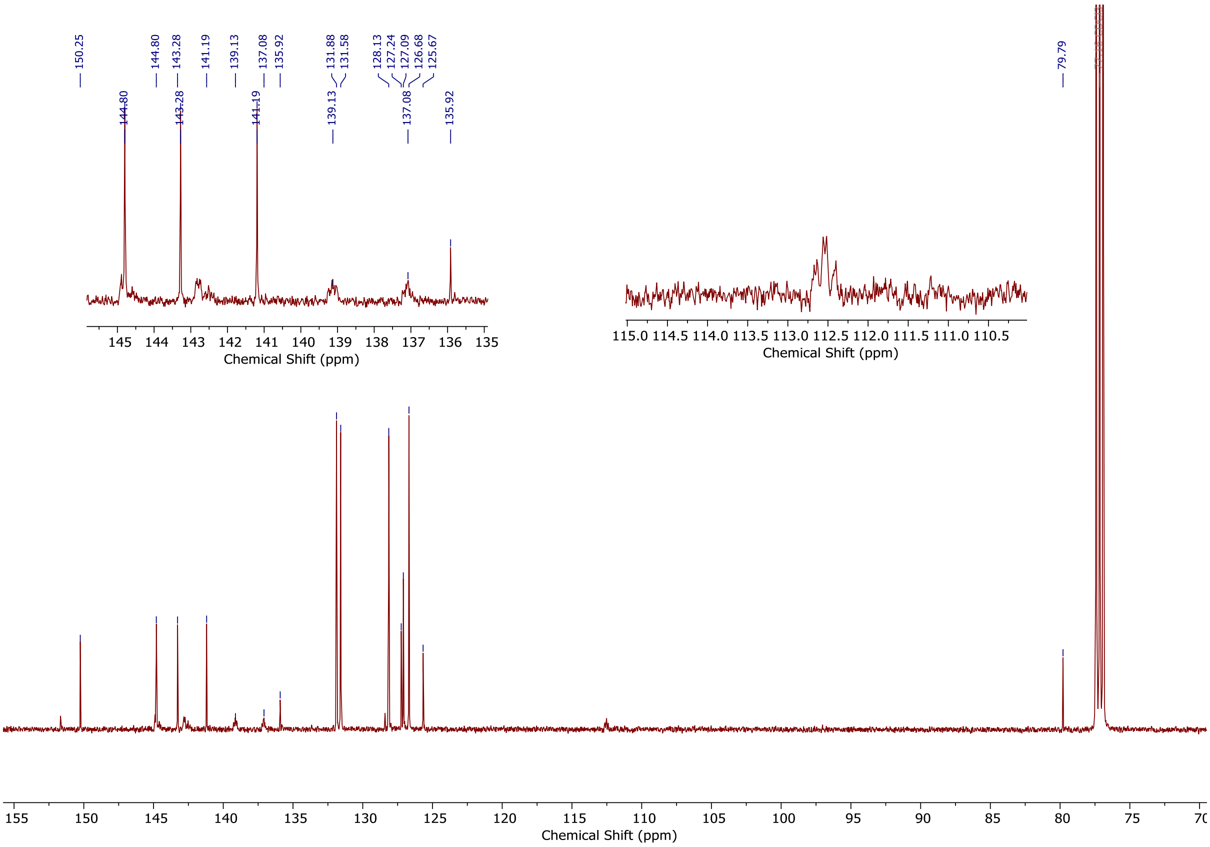

Figure S23. ^{13}C NMR spectrum of **2·XB**^{Z} ($CDCl_3$, 126 MHz, 298K).

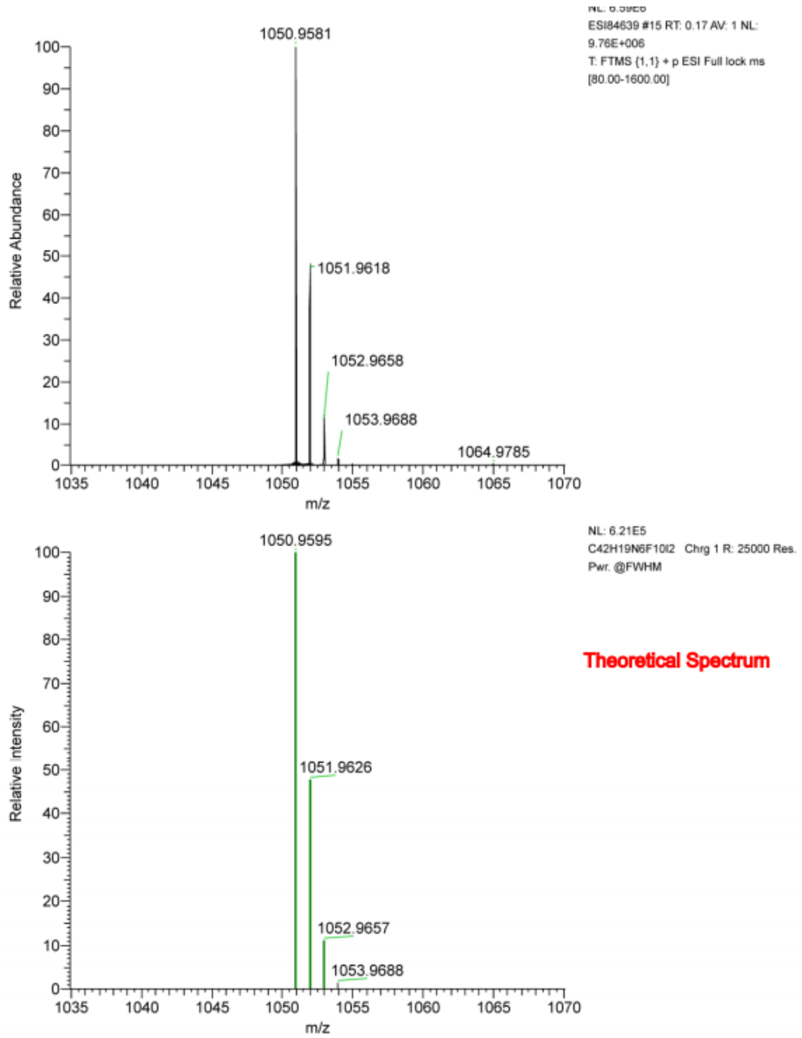

Figure S24. HRESI of **2·XB**^{Z}.

4·XB^{Ph}

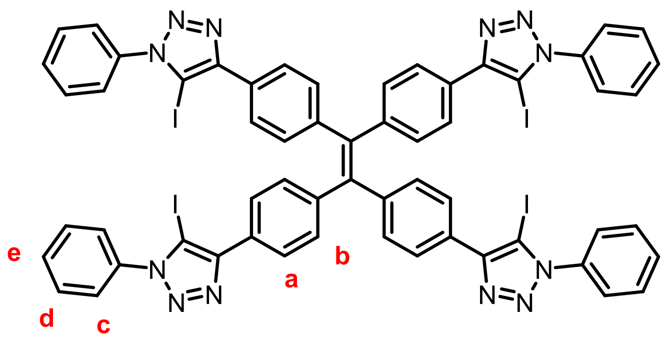

Isolated as yellow solid (56%).

^{1}H NMR (400 MHz, $CDCl_3$) δ 7.89 – 7.80 (m, 8H_a), 7.61 – 7.48 (m, 20$H_{c,d,e}$), 7.32 – 7.27 (m, 4H_b).

^{13}C NMR (126 MHz, $CDCl_3$) δ 150.32, 143.83, 141.43, 137.18, 131.90, 130.33, 129.49, 128.73, 127.44, 126.66, 78.30.

HRMS (ESI+ve) m/z: 1408.9425 ($[M+H]^+$, $C_{58}H_{37}I_4N_{12}$ requires 1408.9437).

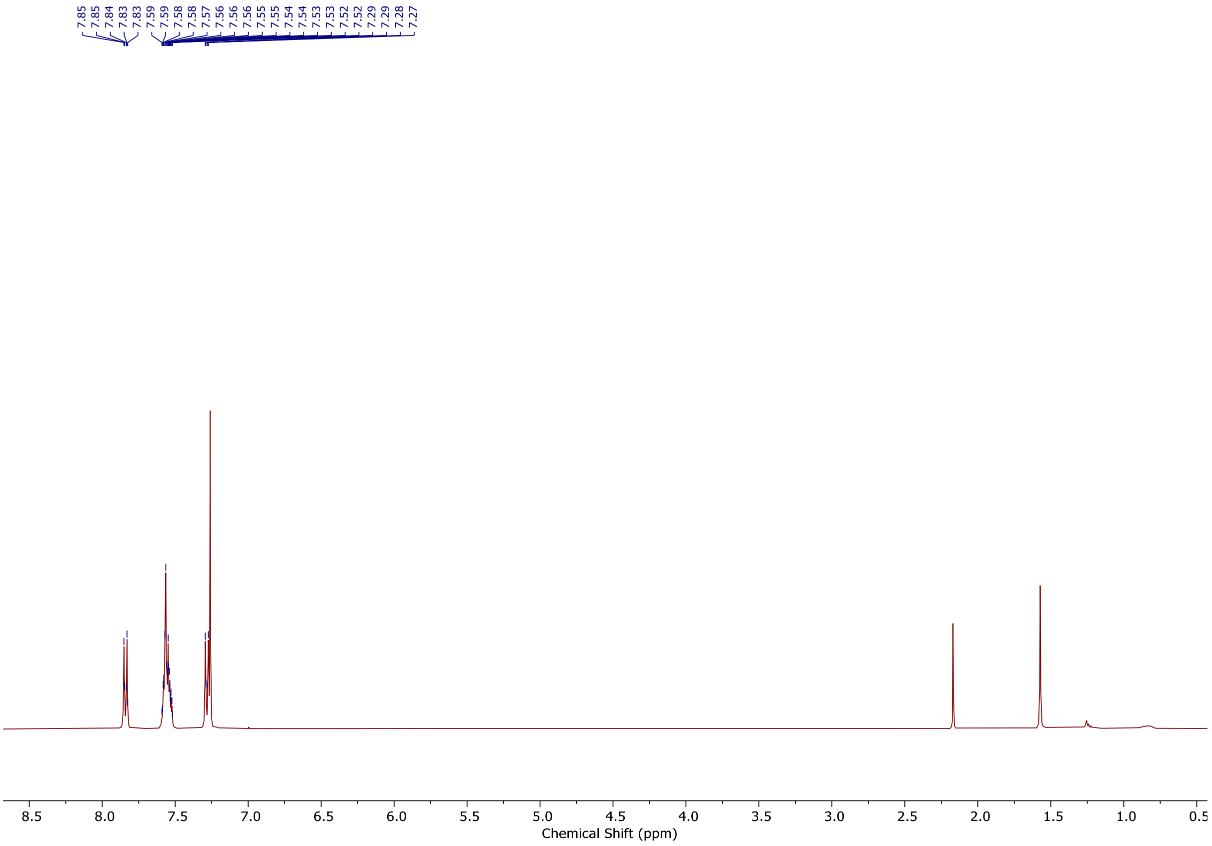

Figure S25. ^{1}H NMR spectrum of **4·XB^{Ph}** ($CDCl_3$, 400 MHz, 298K).

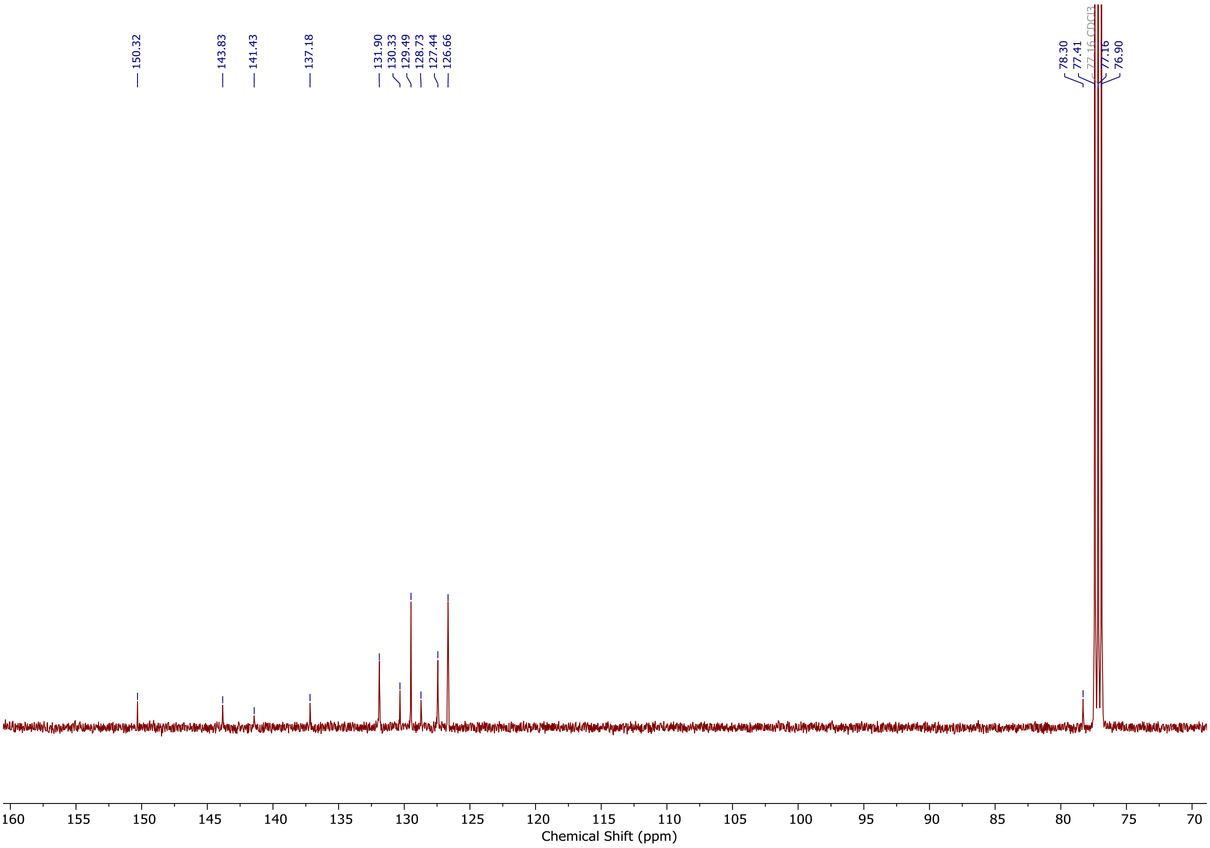

Figure S26. ^{13}C NMR spectrum of **4·XB^{Ph}** ($CDCl_3$, 126 MHz, 298K).

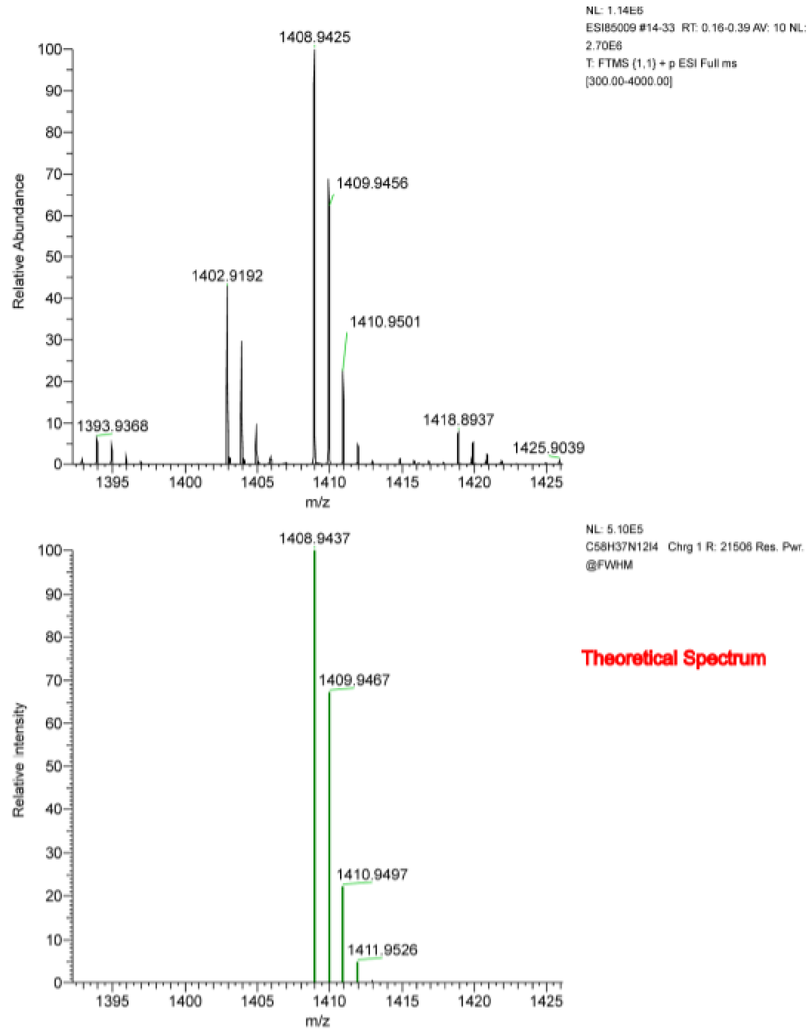

Figure S27. HRESI of **4·XB**$^{\mathbf{Ph}}$.

3. ^{1}H NMR Titration Experiments

Titration protocol: In a typical ^{1}H NMR anion titration experiment, aliquots of anion were added to a THF-d_8 solution of the receptor, conducted such that the receptor solution concentration remained constant. THF solvent was used to facilitate direct comparison with behaviour in fluorescence experiments. Errors in the fitting were estimated to be <10% in each case.

[Host] = 1 mM
[TBACl] or [TBABr] = 25 mM
[TBAI] = 12.5 mM
Spectra were recorded at 0, 0.2, 0.4, 0.6, 0.8, 1.0, 1.2, 1.4, 1.6, 1.8, 2.0, 2.5, 3.0, 4.0, 5.0, 7.0 and 10 equivalents.

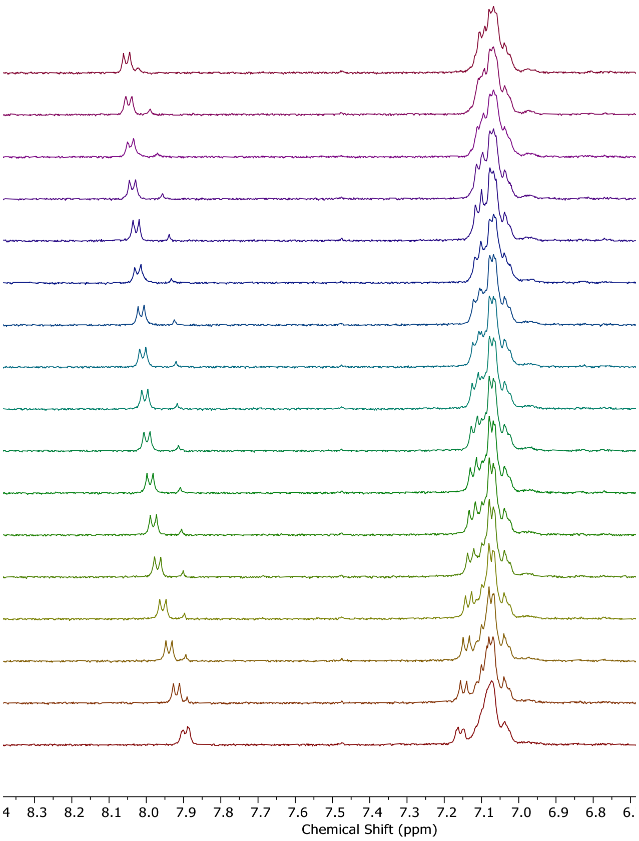

Figure S28. Stacked ^{1}H NMR TBACl titration of **1·XB** (1mM).

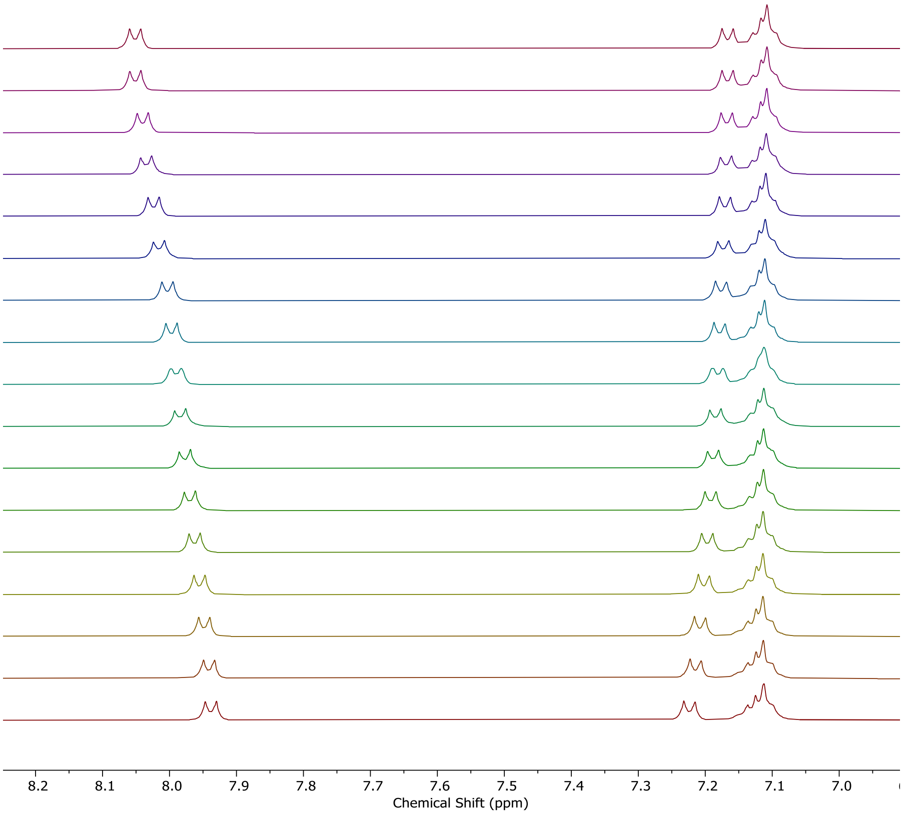

Figure S29. Stacked ^{1}H NMR TBACl titration of **2·XB** (1mM).

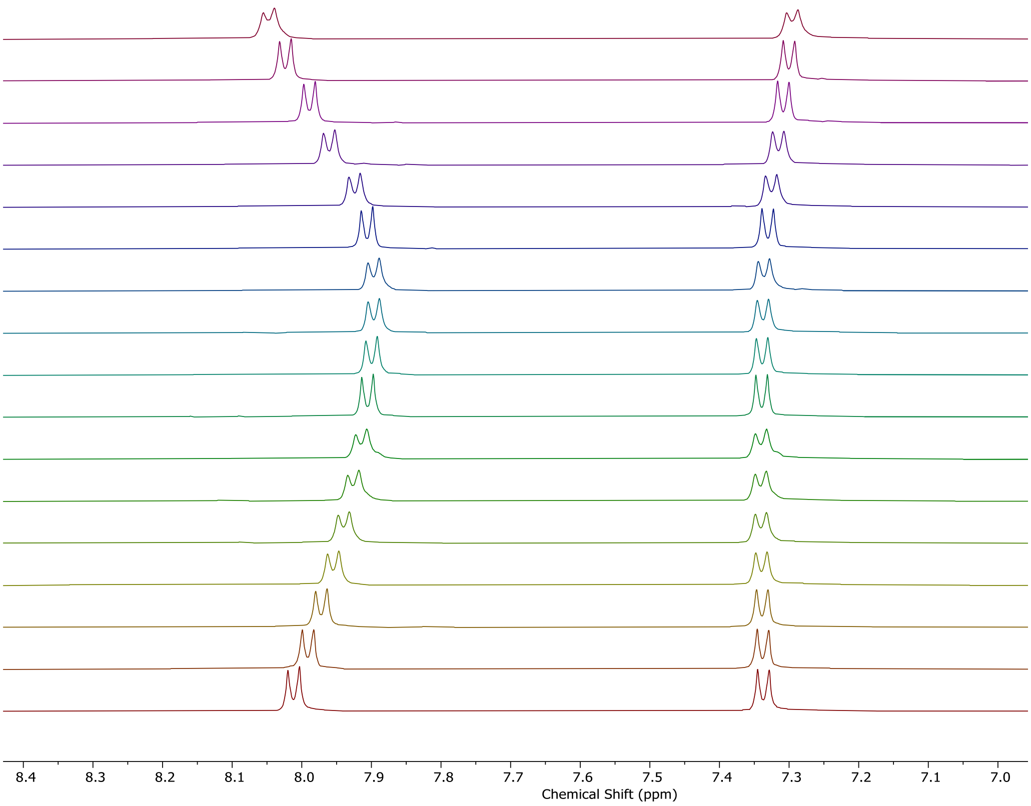

Figure S30. Stacked ^{1}H NMR TBACl titration of **4·XB** (1mM).

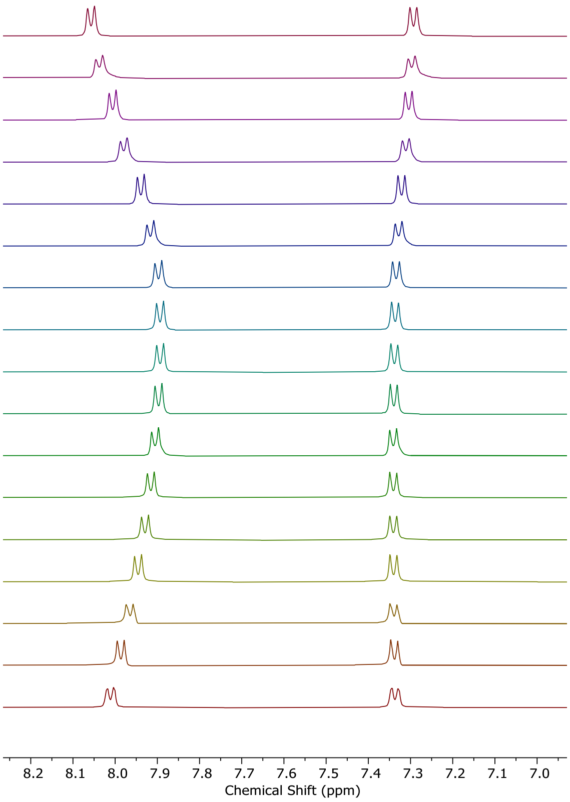

Figure S31. Stacked ^{1}H NMR TBACl titration of **4·XB** (0.75mM).

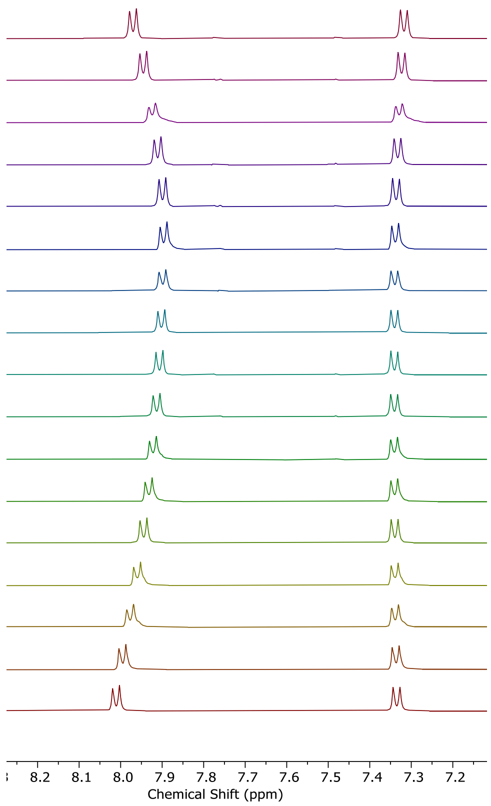

Figure S32. Stacked ^{1}H NMR TBACl titration of **4·XB** (0.50mM).

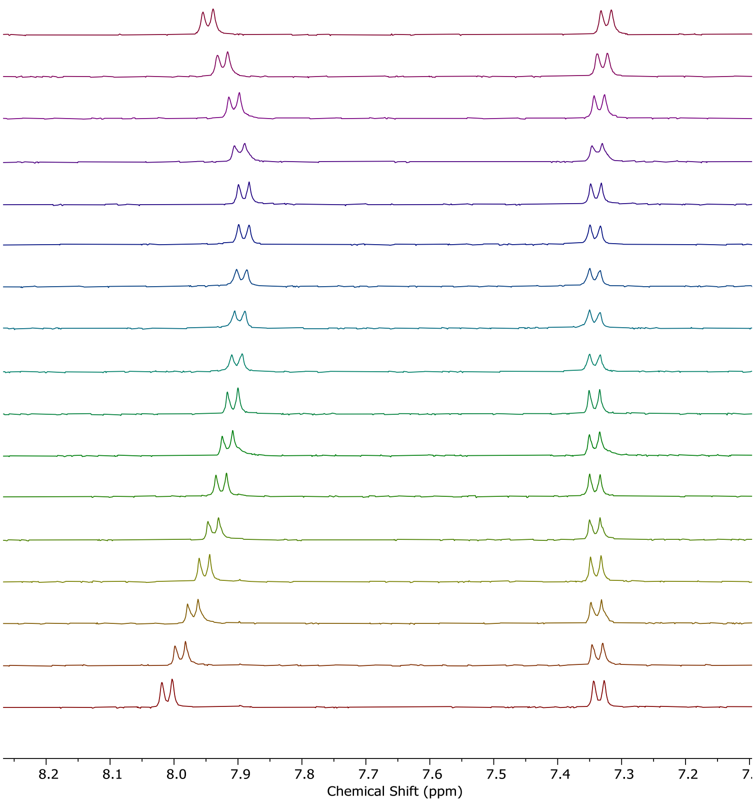

Figure S33. Stacked ^{1}H NMR TBACl titration of **4·XB** (0.25mM).

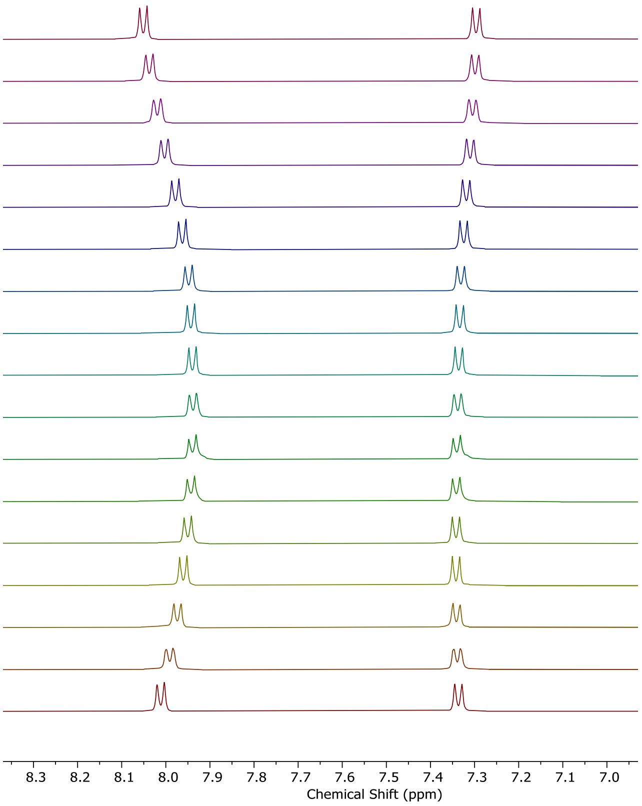

Figure S34. Stacked ^{1}H NMR TBABr titration of **4·XB** (1mM).

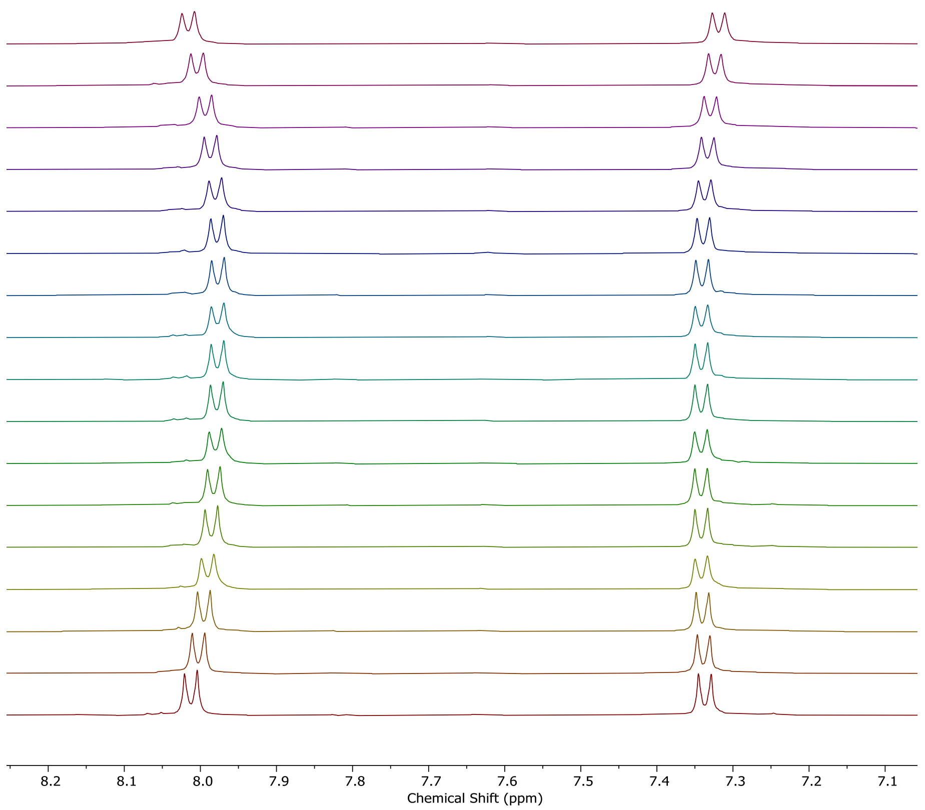

Figure S35. Stacked ^{1}H NMR TBAI titration of **4·XB** (1mM).

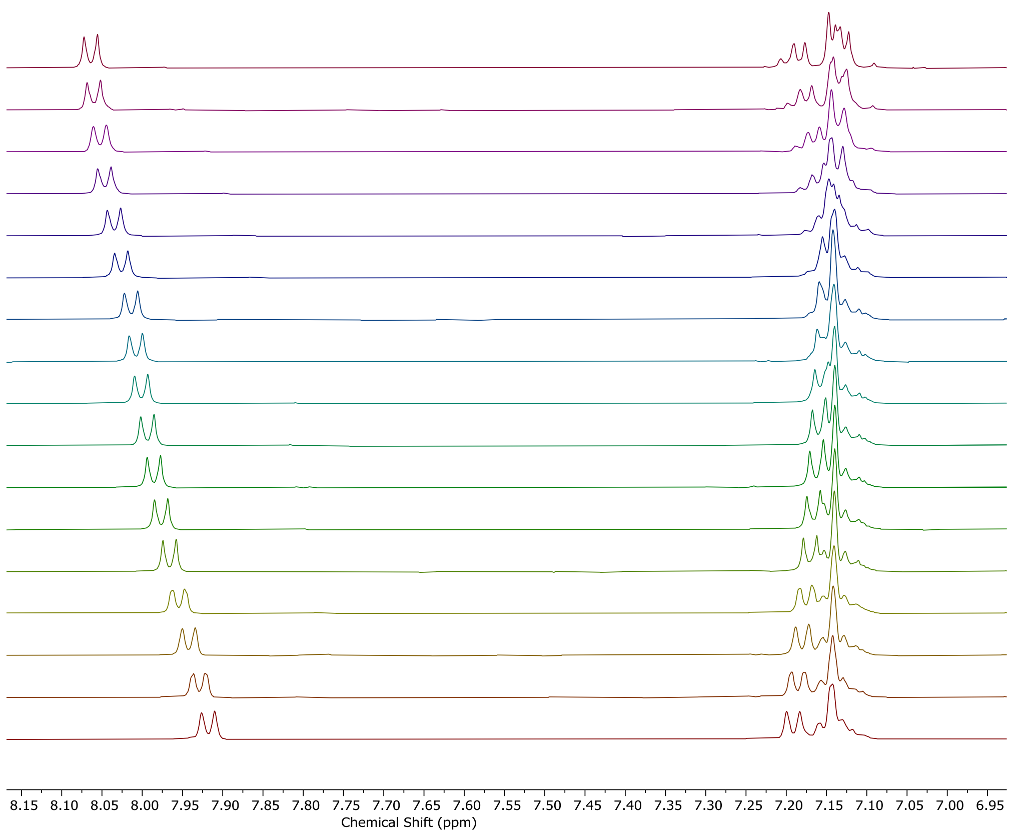

Figure S36. Stacked ^{1}H NMR TBACl titration of **2·XB*^E^*** (1mM).

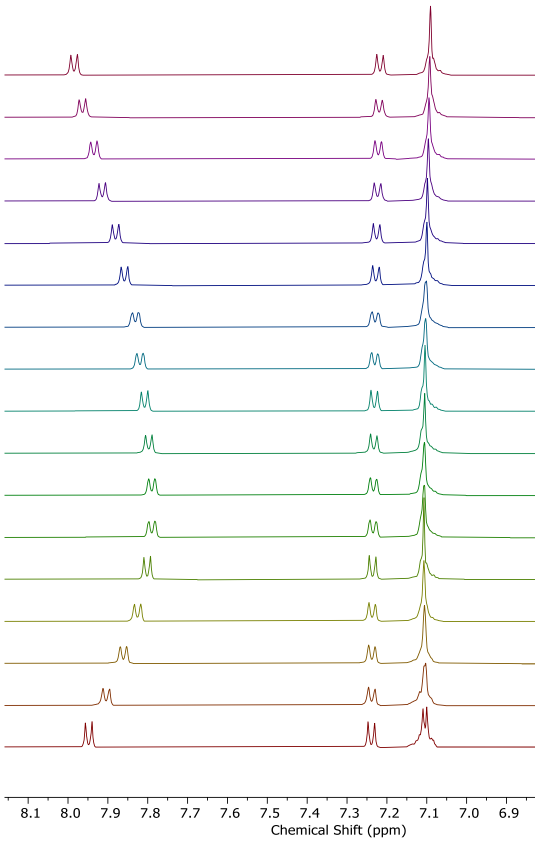

Figure S37. Stacked ^{1}H NMR TBACl titration of **2·XB^Z** (1 mM).

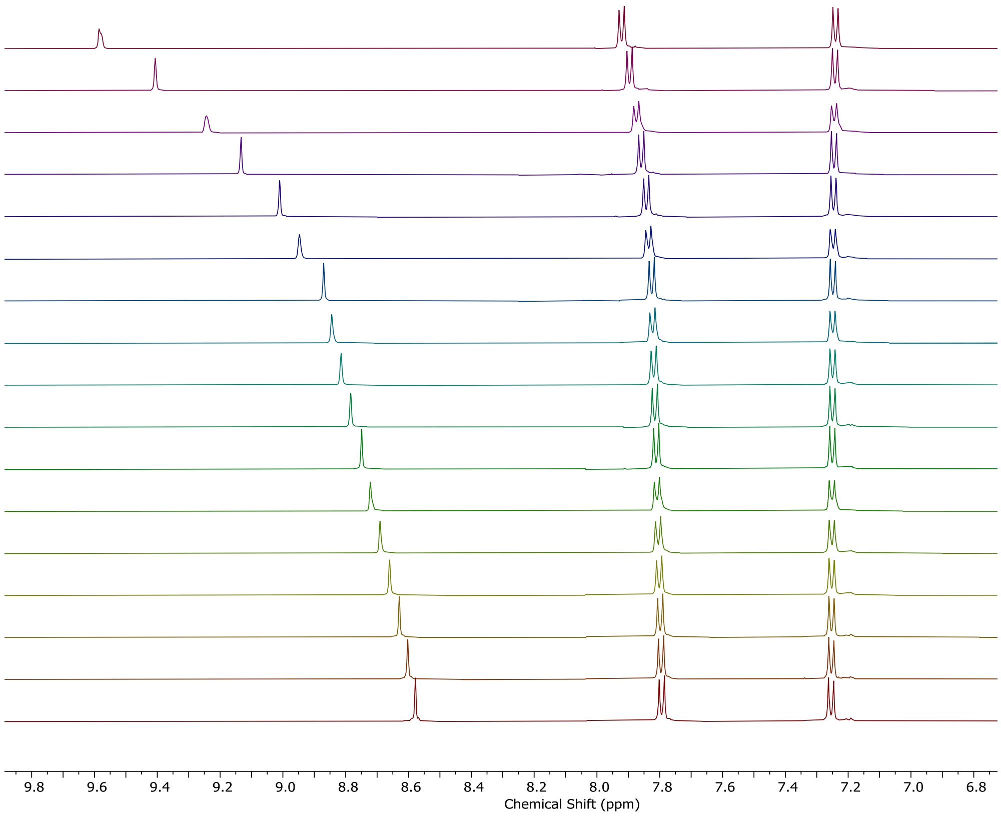

Figure S38. Stacked ^{1}H NMR TBACl titration of **4·HB** (1 mM).

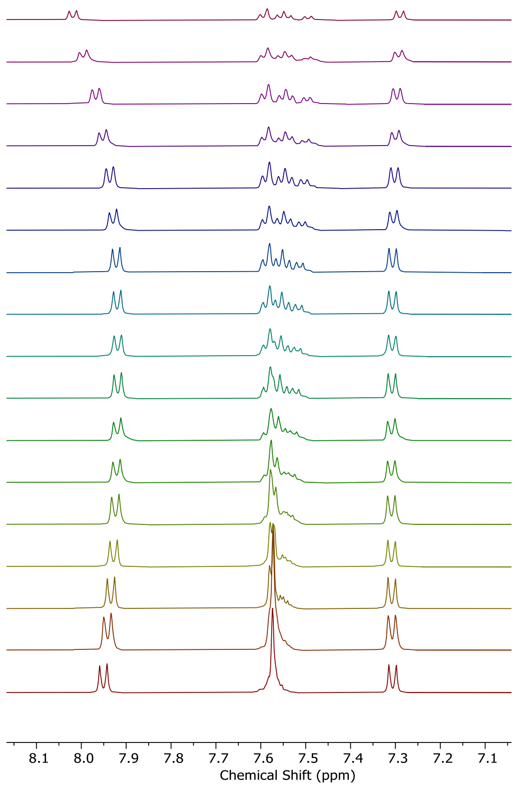

Figure S39. Stacked ^{1}H NMR TBACl titration of **4·XB^{Ph}** (1 mM).

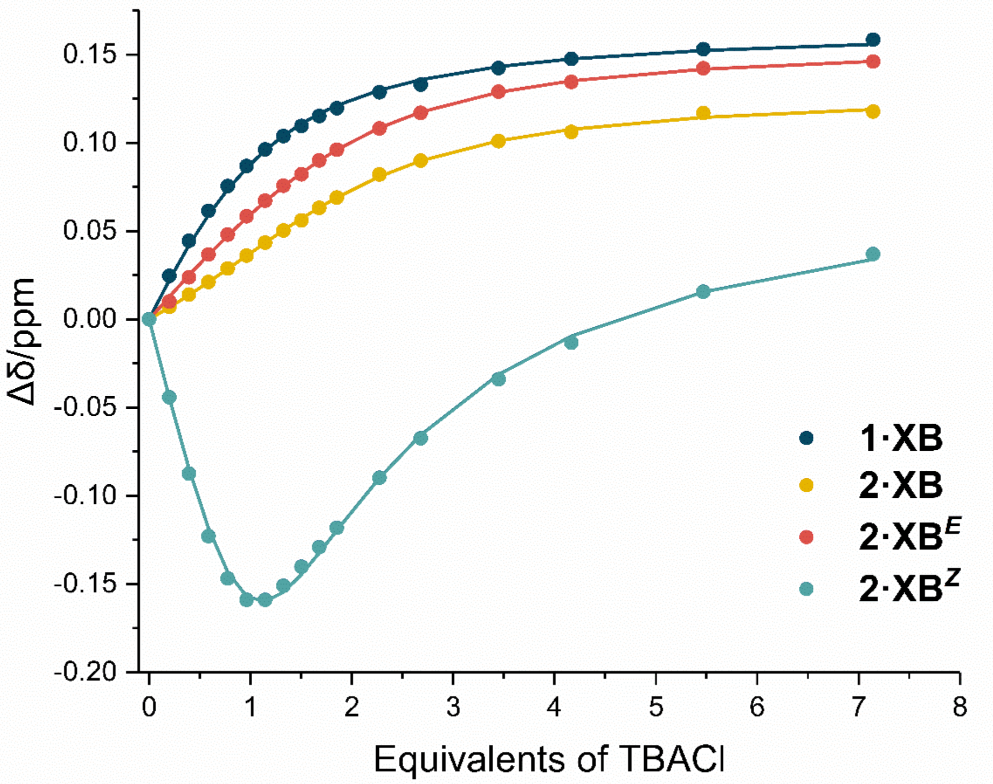

Figure S40. Chloride binding isotherms for **1·XB, 2·XB**, **2·XB**^{E} and **2·XB**^{Z}, where circles represent experimental data and solid lines represent the fitted binding isotherm.

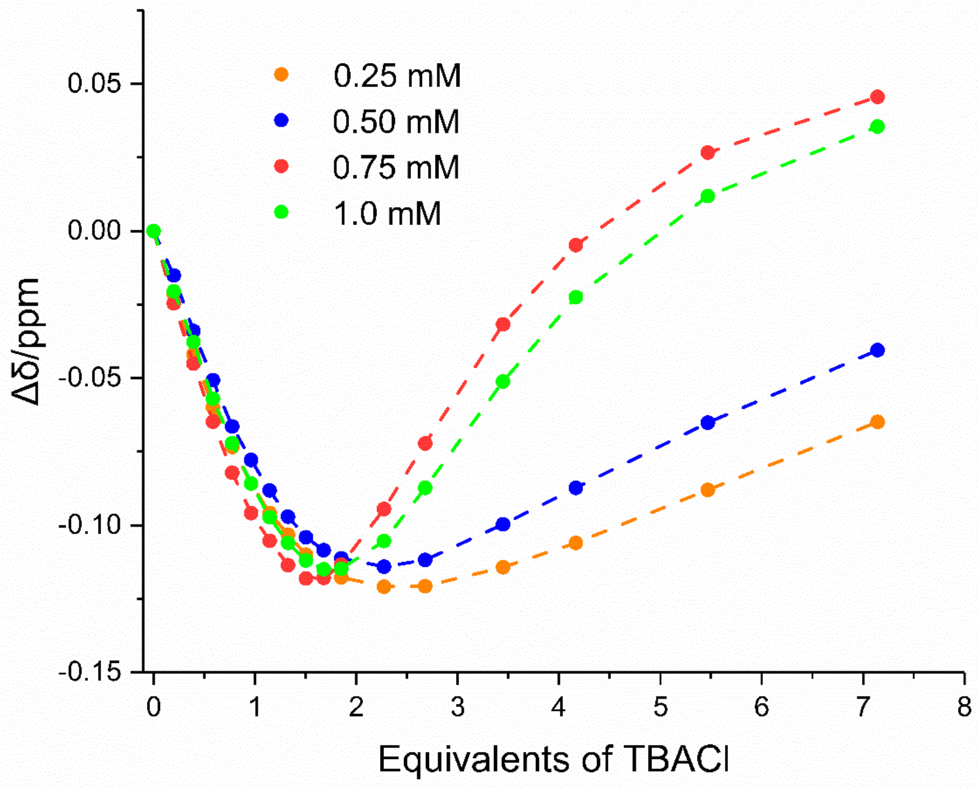

Figure S41. Binding Isotherms derived from ^{1}H NMR TBACl titrations of **4·XB** (THF-d_8) at concentrations ranging from 0.25-1.00 mM, where circles represent experimental data and dashed lines are a visual aid.

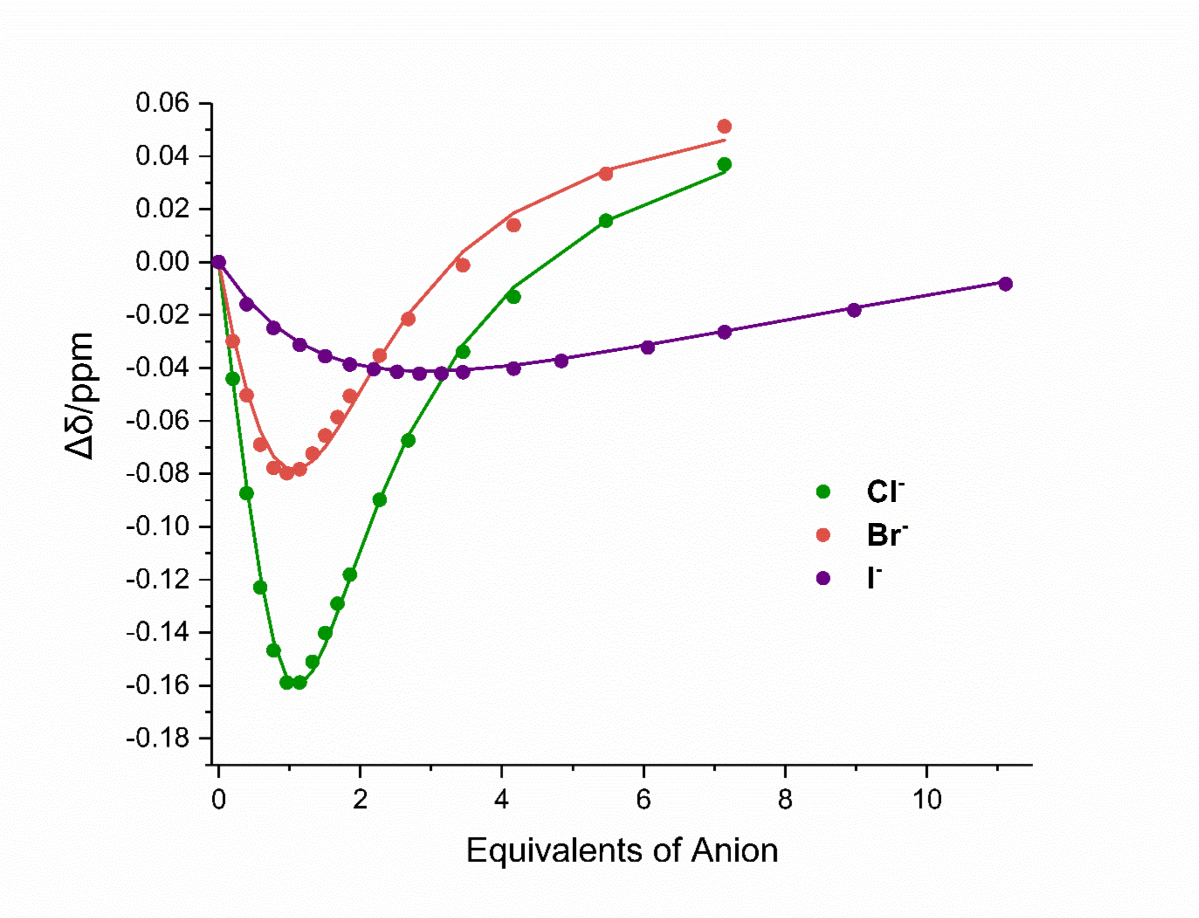

Figure S42. Halide Binding Isotherms derived from ^{1}H NMR TBACl titrations of **2·XB**^{Z} (THF-d_8), where circles represent experimental data and solid lines represent the fitted binding isotherm.

Replacement of the XB donor iodotriazole motifs in **4·XB** with the analogous CH HB donor (compound **4·HB**) led to weak binding (Fig S43), as indicated by the near linear binding isotherm. This is in contrast to **4·XB** in which an inflection point after two equivalents of anion indicates strong (>10^4 M^{-1}) binding of each of the first two equivalents of anion.

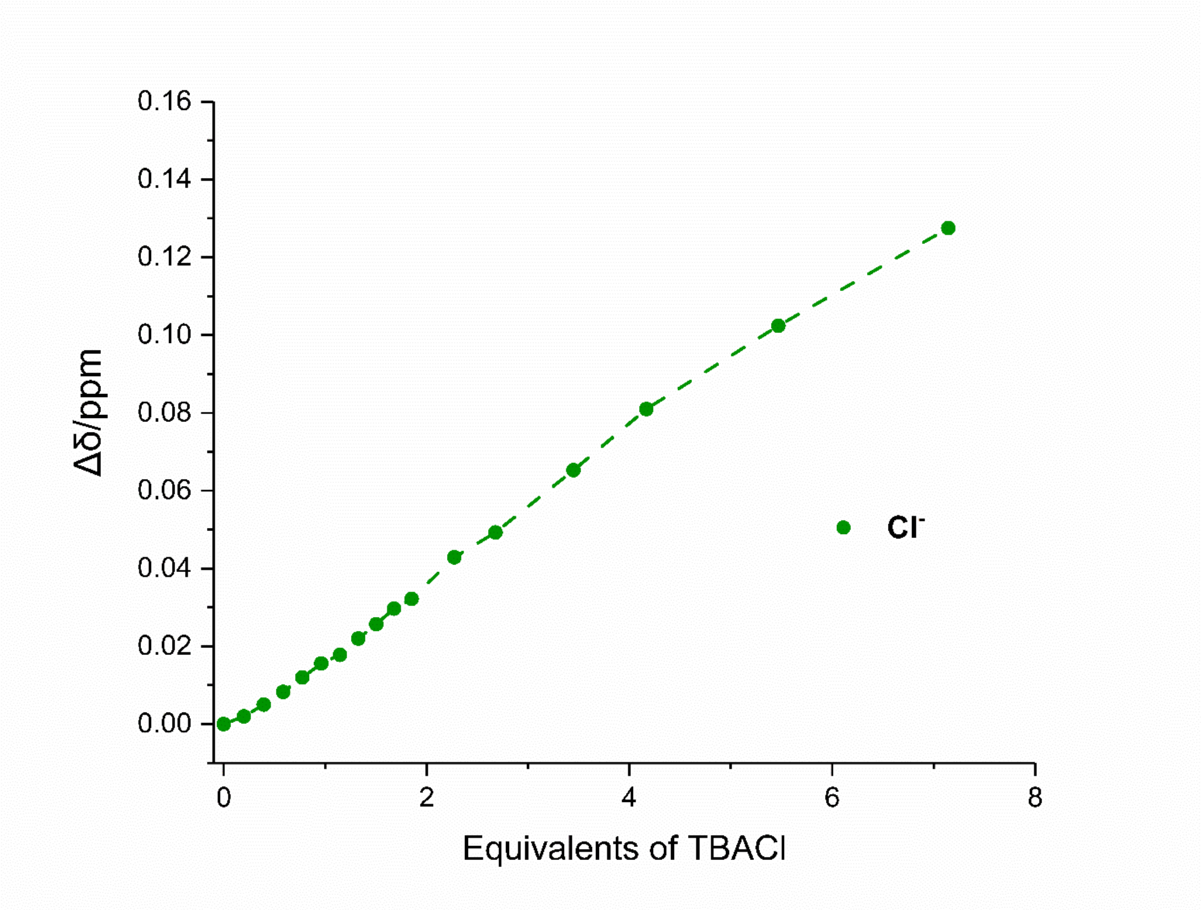

Figure S43. Binding Isotherm derived from ^{1}H NMR TBACl titrations of **4·HB** (THF-d_8) where circles represent experimental data and dashed lines are a visual aid.

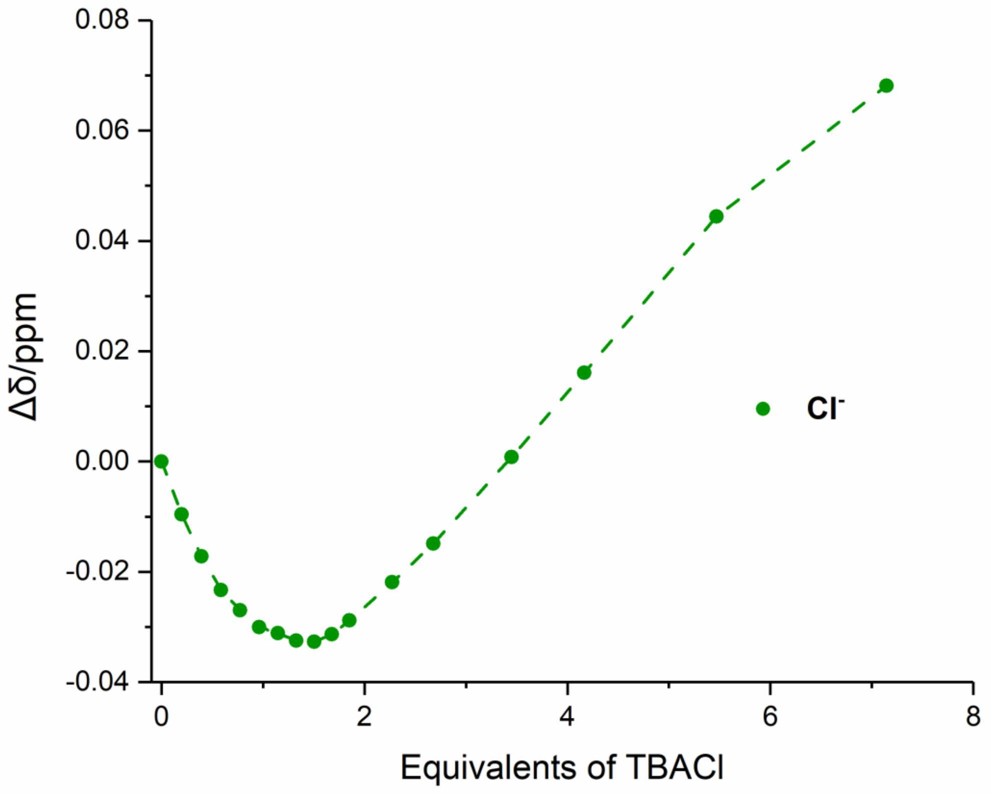

Figure S44. Binding Isotherm derived from ^{1}H NMR TBACl titrations of **4·XB^{Ph}** (THF-d_8) where circles represent experimental data and dashed lines are a visual aid.

As described by Thordarson et al.[9] the suitability of the 1:2 binding model flavour (shown below) chosen for **2·XB**, **2·XB^{E}** and **2·XB^{E}** ^{1}H NMR titration data was assessed through comparison of a quality of fit indicator, variance of fit, or Cov_{fit}. Importantly, in the instance when a more complex binding flavour was selected (i.e. more parameters), the selection is justified by an improved Cov_{fit} ratio (at least three to five times higher) than the less complex model. The tables below summarise the values with their Cov_{fit} ratios relative to the statistical 1:2 binding model, in which the selected flavour is highlighted in green and the physically impossible results from the selected flavours are highlighted in red.

Binding model:

Statistical 1:2 — $K_1 = 4K_2$; $\delta_{\Delta HG} = 2\delta_{\Delta HG}$

Non-cooperative1:2 — $K_1 = 4K_2$; $\delta_{\Delta HG} \neq 2\delta_{\Delta HG}$

Additive 1:2 — $K_1 \neq 4K_2$; $\delta_{\Delta HG} = 2\delta_{\Delta HG}$

Full 1:2 — $K_1 \neq 4K_2$; $\delta_{\Delta HG} \neq 2\delta_{\Delta HG}$

Table S1. Summary of Bindfit output data for **2·XB** with TBACl from ^{1}H NMR titration (THF-d$_8$, 1 mM).

Model	K_{11}	K_{12}	Cov_{fit} ratio
**Full**	0.0520	471098	2.28
**Non-Cooperative**	5647	1412	13.54
**Additive**	895	2580	10.09
**Statistical**	1870	468	1.00

Table S2. Summary of Bindfit output data for **2·XB^{E}** with TBACl from ^{1}H NMR titration (THF-d$_8$, 1 mM).

Model	K_{11}	K_{12}	Cov_{fit} ratio
**Full**	11070	1157	1.10
**Non-Cooperative**	322	80	0.22
**Additive**	371	-28	0.19
**Statistical**	4972	1243	1.00

Table S3. Summary of Bindfit output data for **2·XB^{Z}** with TBACl from ^{1}H NMR titration (THF-d$_8$, 1 mM).

Model	K_{11}	K_{12}	Cov$_{fit}$ ratio
**Full**	23216	963	40.6
**Non-Cooperative**	7085	1771	12.8
**Additive**	2446539	-170	1.0
**Statistical**	2446539	-170	1.0

4. DLS experiments

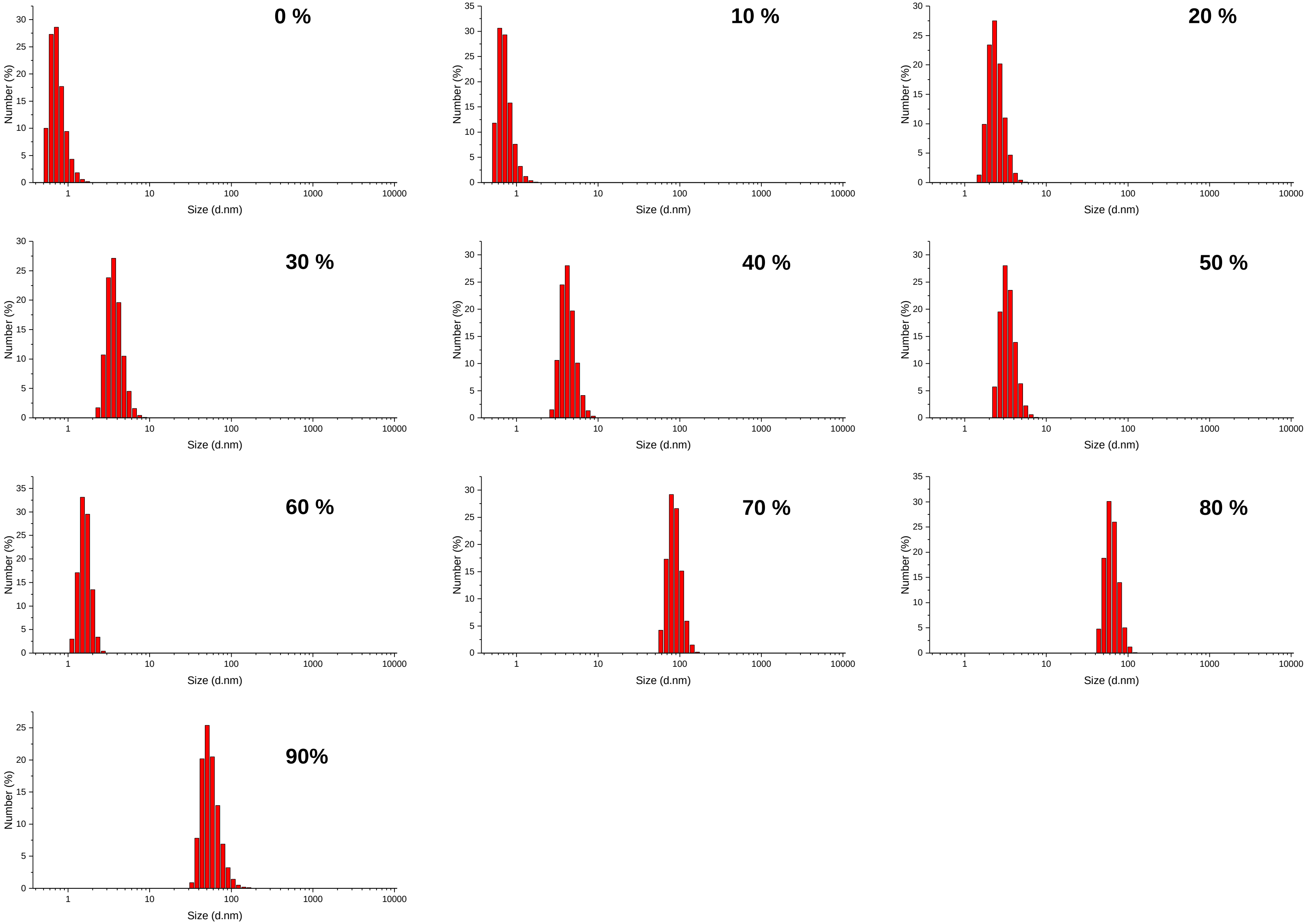

Figure S45. Dynamic Light Scattering profiles for **4·HB** with varying water fractions (f_w) in THF solution (10^{-5} M).

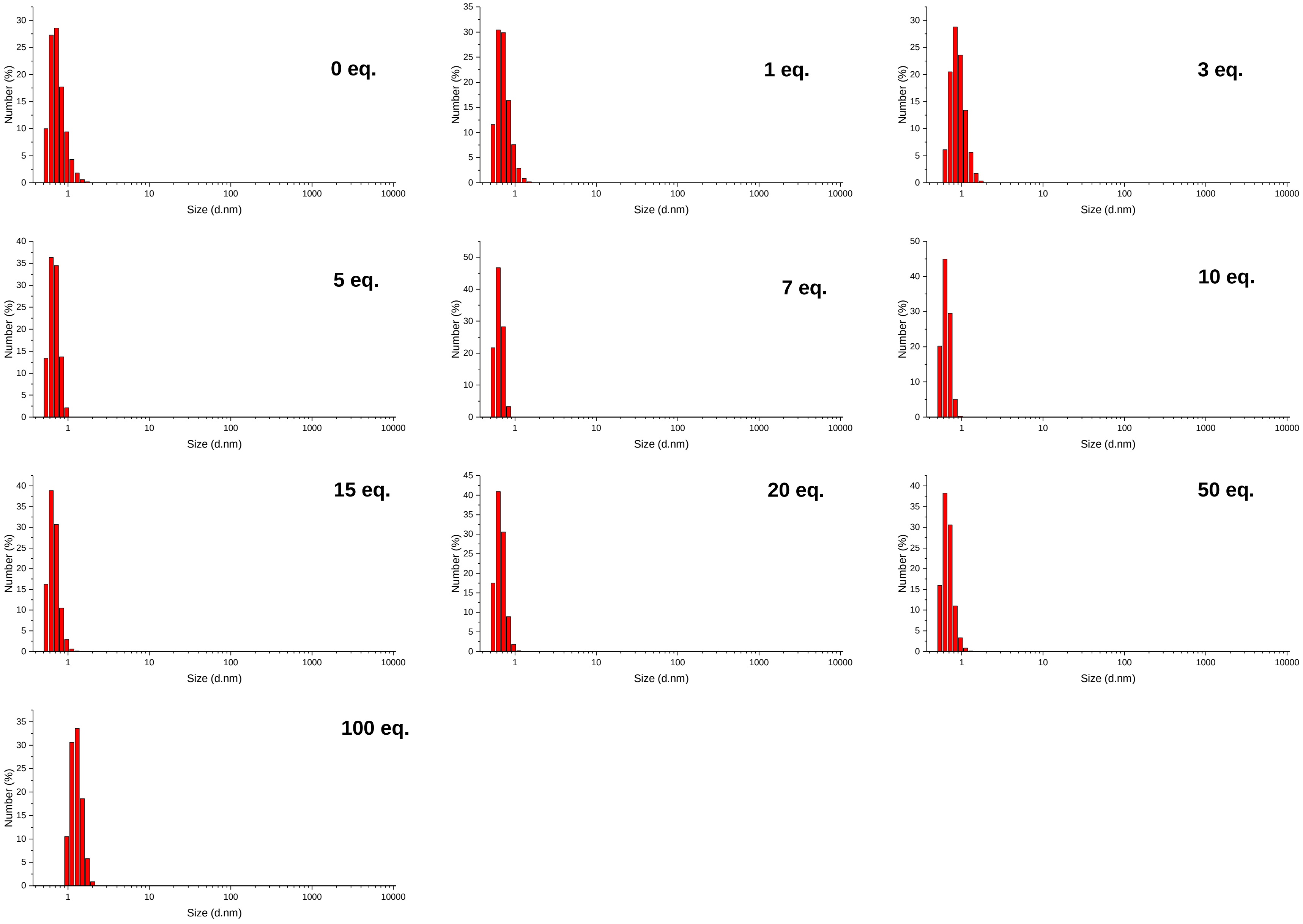

Figure S46. Dynamic Light Scattering profiles for **4·HB** with varying equivalents of TBACl in THF solution (10^{-5} M).

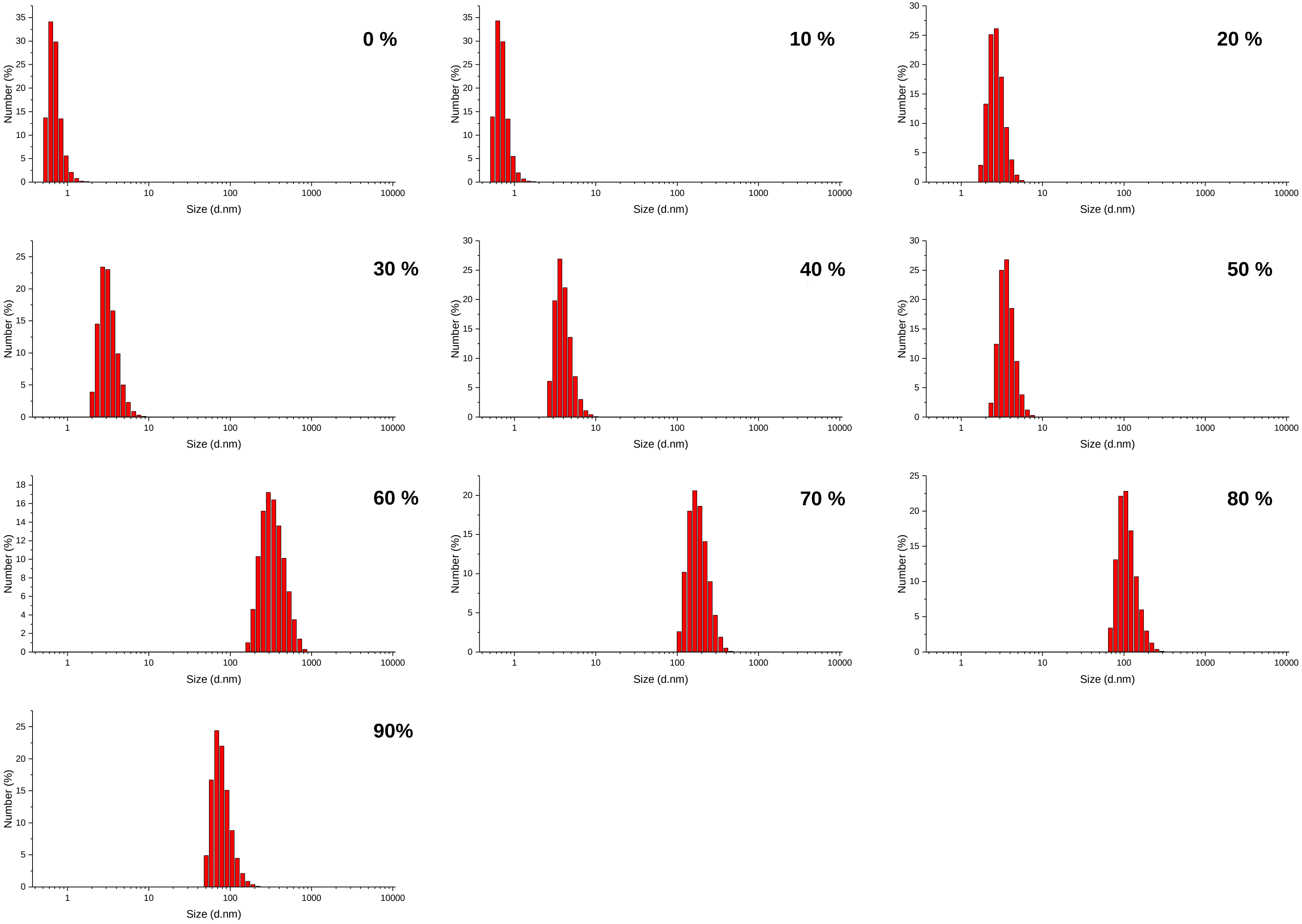

Figure S47. Dynamic Light Scattering profiles for **4·XB** with varying water fractions (f_w) in THF solution (10^{-5} M).

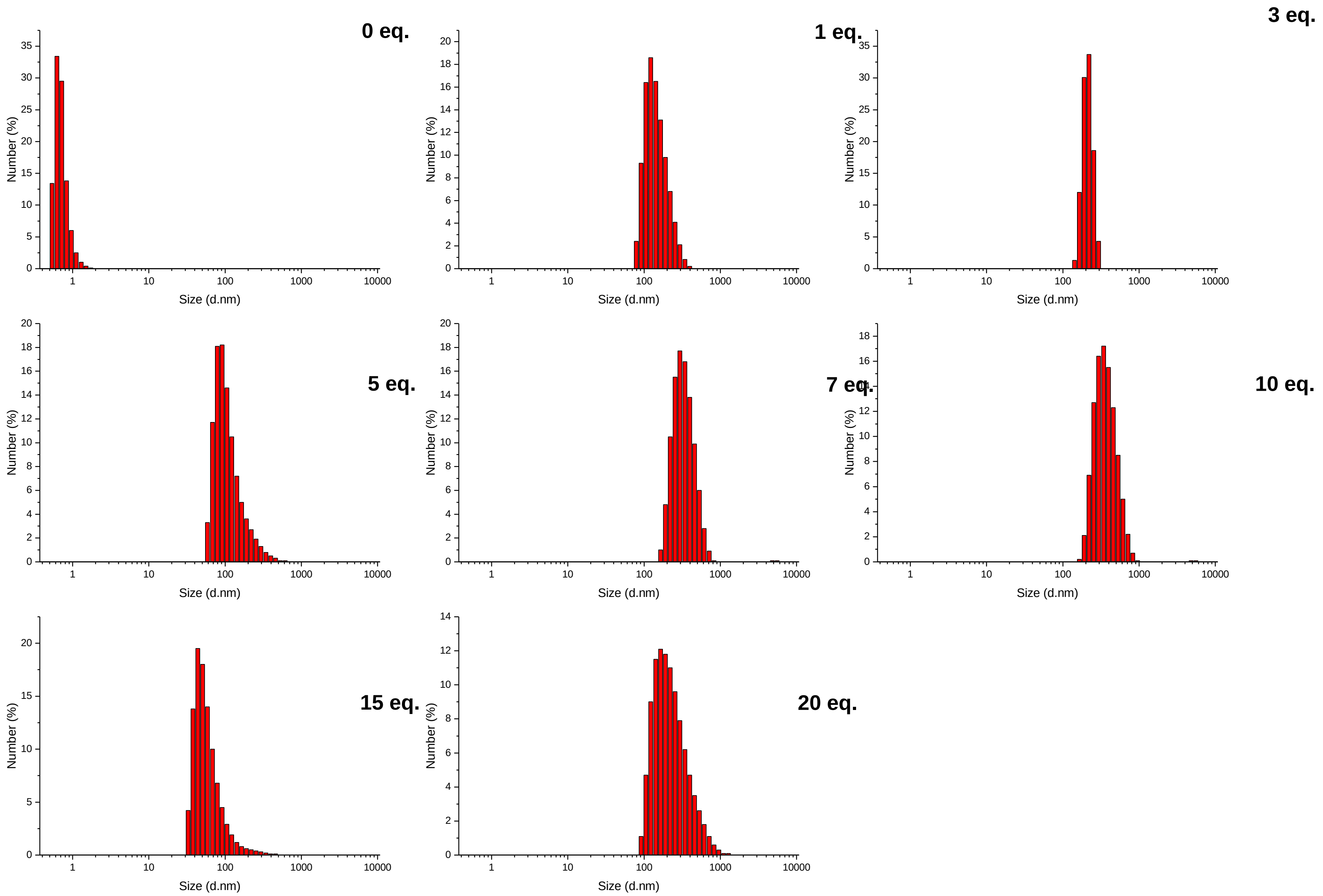

Figure S48. Dynamic Light Scattering profiles for **4·XB** with varying equivalents of TBACl in THF solution (10^{-5} M).

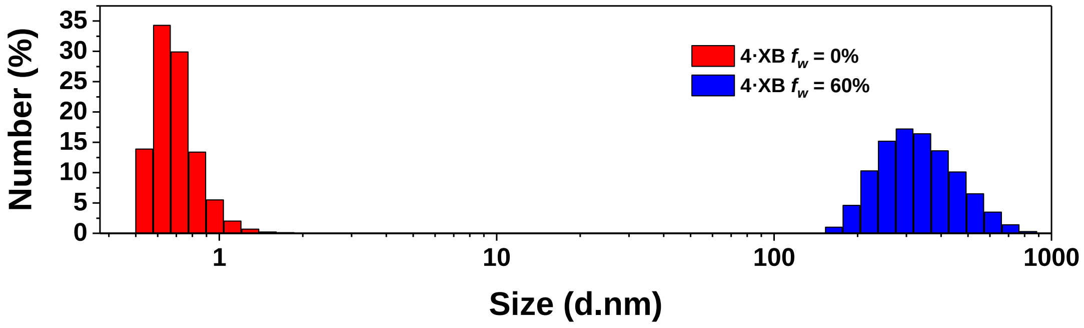

Figure S49. Stacked dynamic light scattering profiles for **4·XB** (10^{-5} M) in THF and THF:H_2O (4:6, v/v).

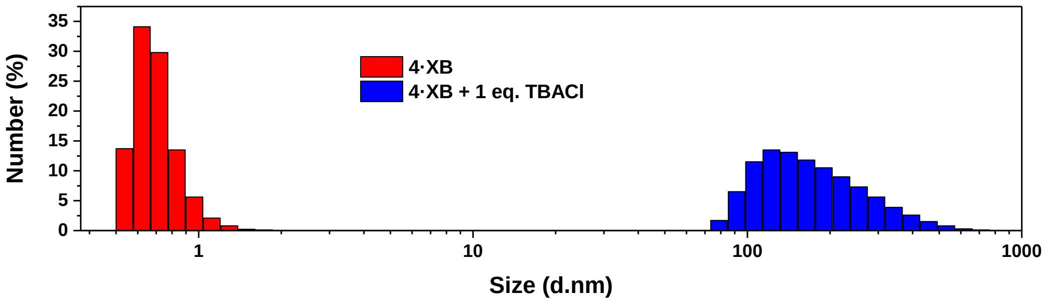

Figure S50. Stacked dynamic light scattering profiles for **4·XB** (10^{-5} M) in THF in the absence and presence of 1 equivalent of TBACl.

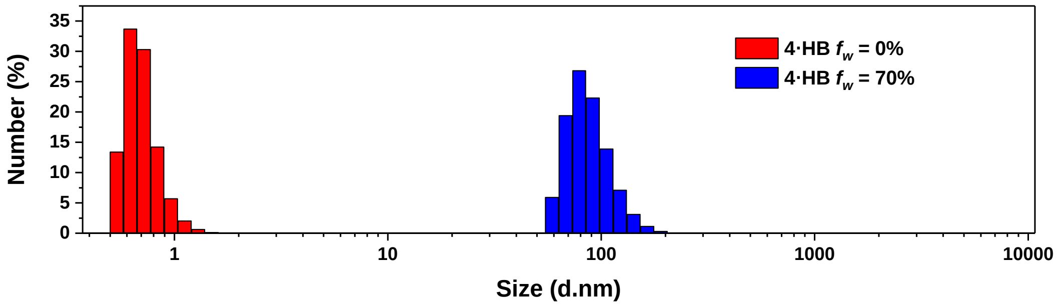

Figure S51. Stacked dynamic light scattering profiles for **4·HB** (10^{-5} M) in THF and THF:H_2O (3:7, v/v).

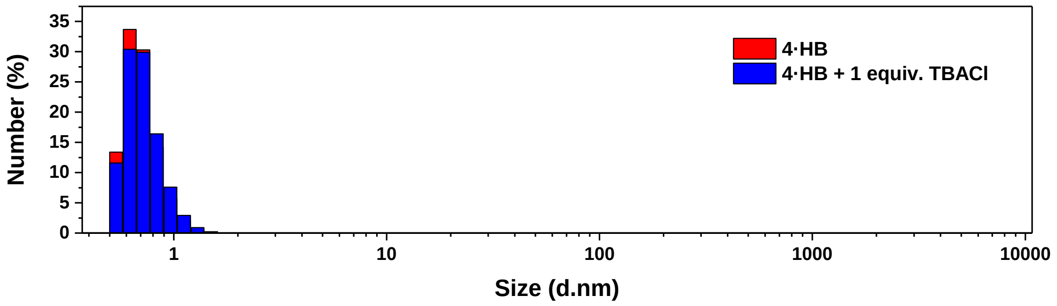

Figure S52. Stacked dynamic light scattering profiles for **4·HB** (10^{-5} M) in THF in the absence and presence of 1 equivalent of TBACl.

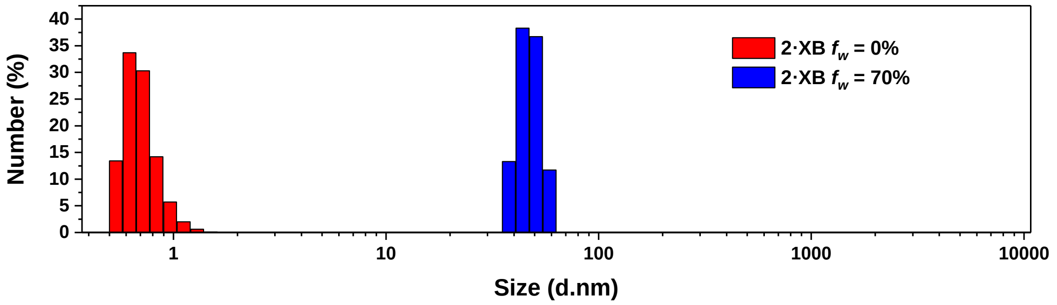

Figure S53. Stacked dynamic light scattering profiles for **2·XB** (10^{-5} M) in THF and THF:H_2O (3:7, v/v).

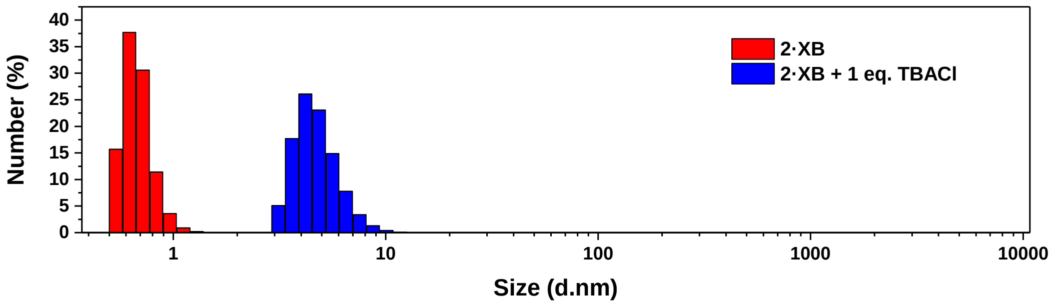

Figure S54. Stacked dynamic light scattering profiles for **2·XB** (10^{-5} M) in THF in the absence and presence of 1 equivalent of TBACl.

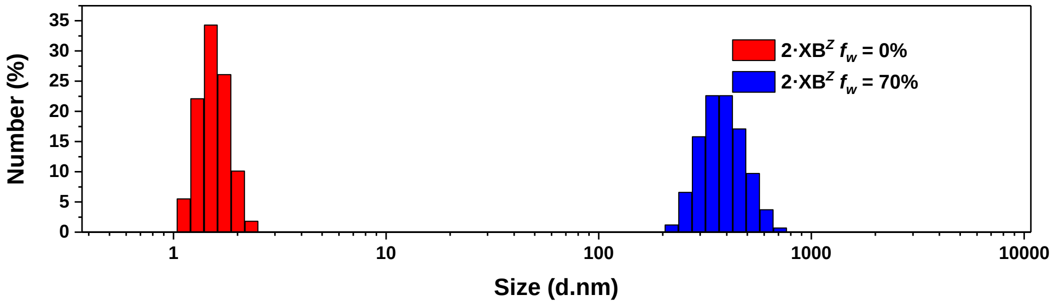

Figure S55. Stacked dynamic light scattering profiles for **2·XB**^{Z} (10^{-5} M) in THF and THF:H_2O (3:7, v/v).

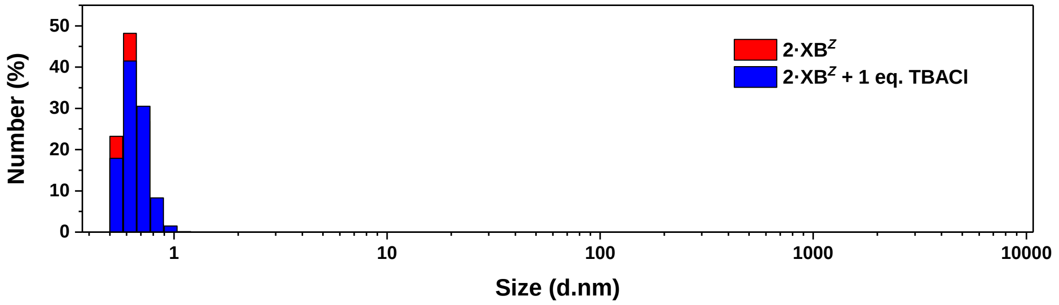

Figure S56. Stacked dynamic light scattering profiles for **2·XB**^{Z} (10^{-5} M) in THF in the absence and presence of 1 equivalent of TBACl.

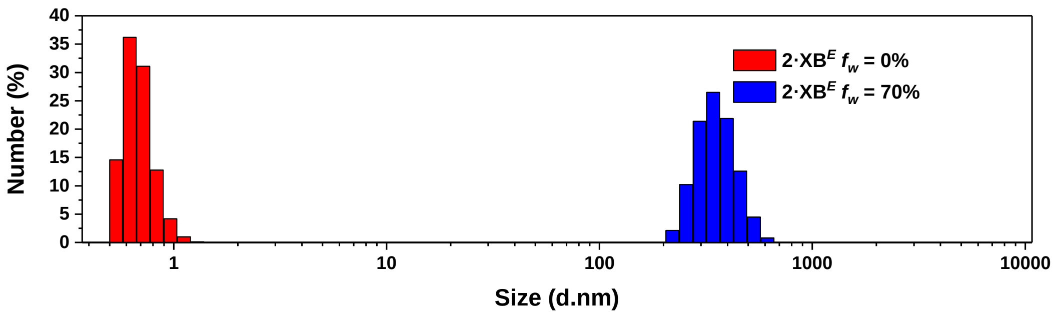

Figure S57. Stacked dynamic light scattering profiles for **2·XB*^{E}*** (10^{-5} M) in THF and THF:H_2O (3:7, v/v).

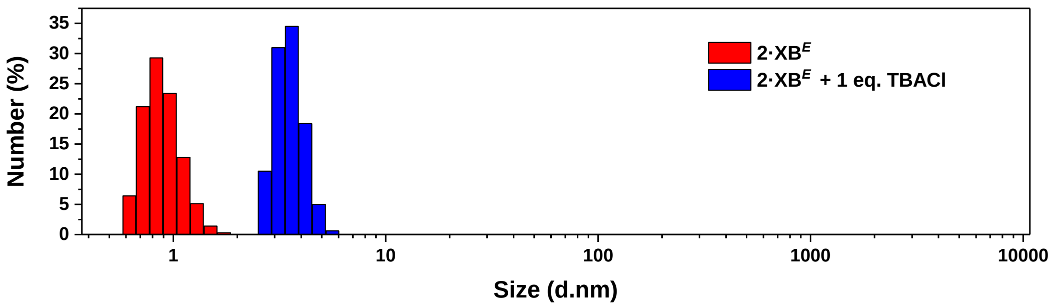

Figure S58. Stacked dynamic light scattering profiles for **2·XB*^{E}*** (10^{-5} M) in THF in the absence and presence of 1 equivalent of TBACl.

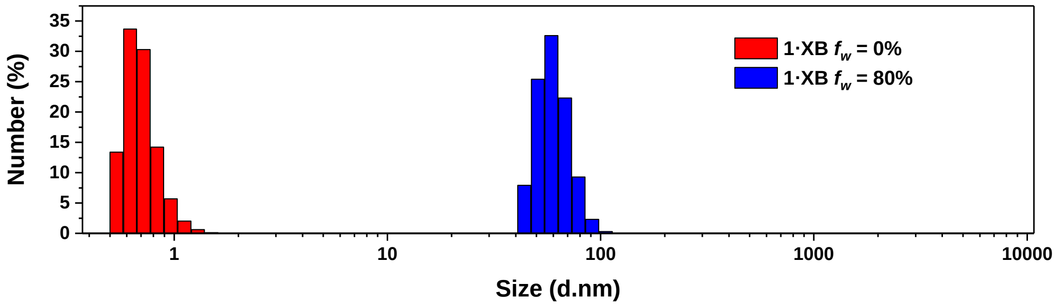

Figure S59. Stacked dynamic light scattering profiles for **1·XB** (10^{-5} M) in THF and THF:H_2O (2:8, v/v).

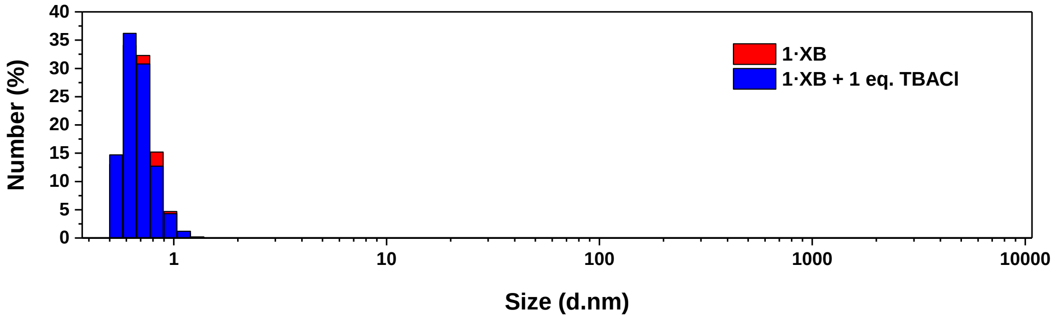

Figure S60. Stacked dynamic light scattering profiles for **1·XB** (10^{-5} M) in THF in the absence and presence of 1 equivalent of TBACl.

5. Absorption and fluorescence experiments

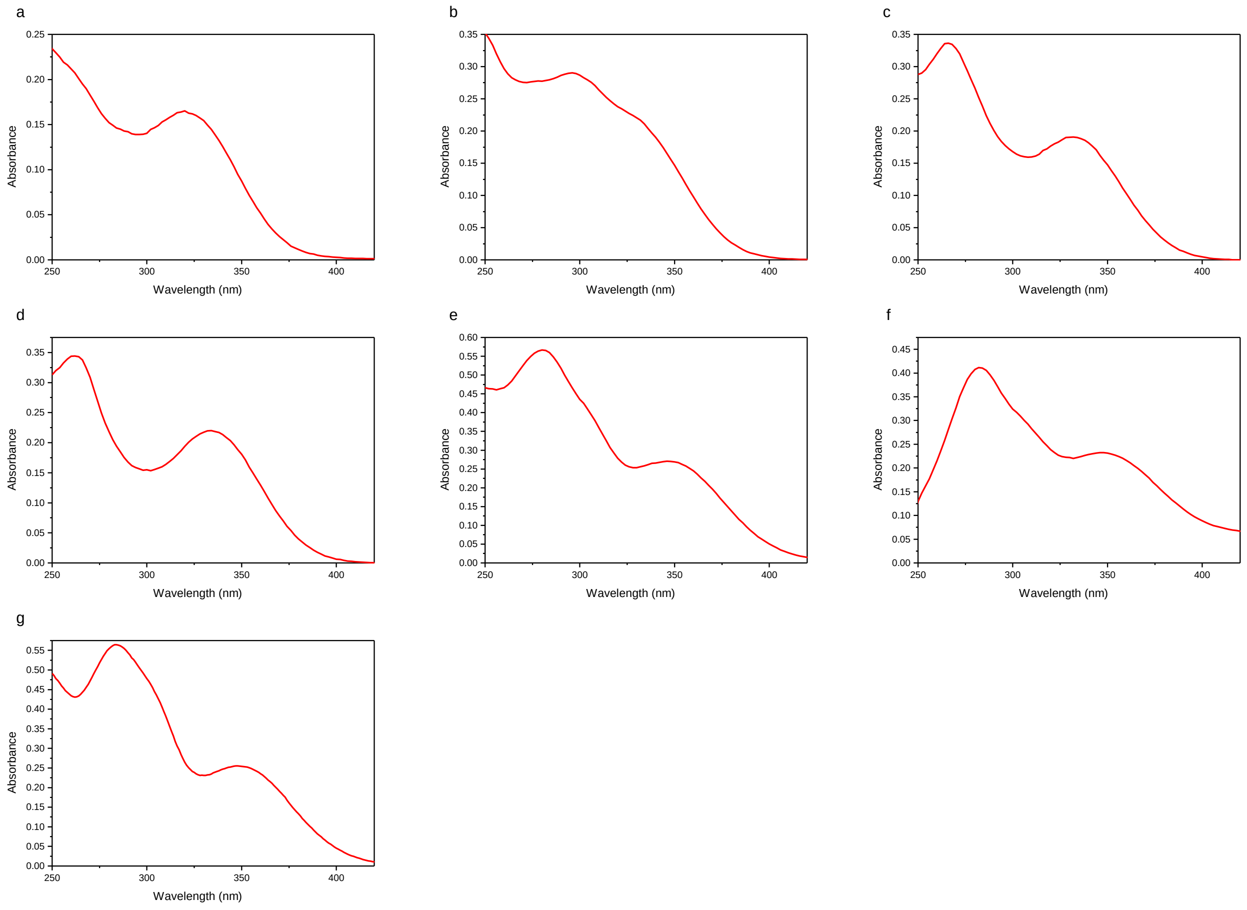

Figure S61. UV-Vis spectra of a) **1·XB** b) **2·XB** c) **2·XB**^{E} d) **2·XB**^{Z} e) **4·XB** f) **4·HB** g) **4·XB**^{Ph} (10^{-5} M) in THF.

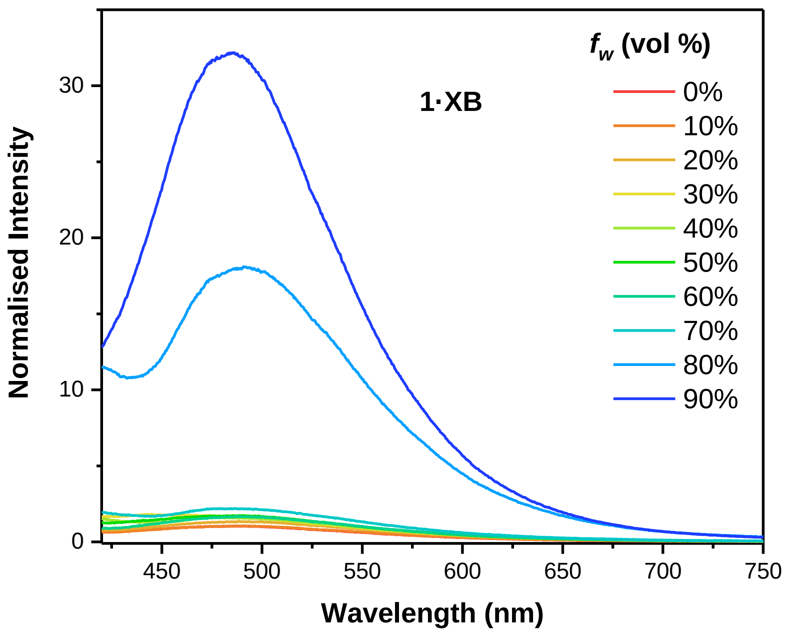

Figure S62. Fluorescence spectrum of **1·XB** (THF, 10^{-5} M, λ_{ex} = 350 nm) with increasing water fraction (f_w).

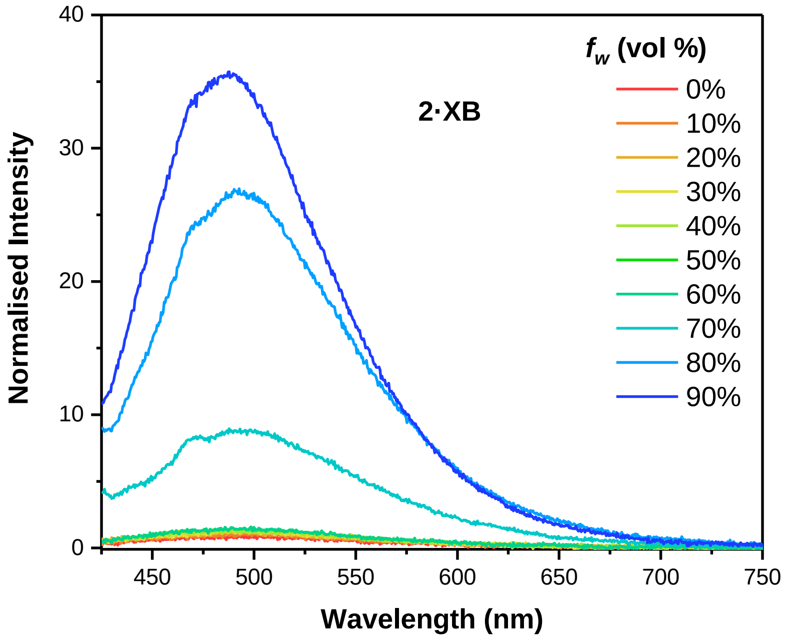

Figure S63. Fluorescence spectrum of **2·XB** (THF, 10^{-5} M, λ_{ex} = 350 nm) with increasing water fraction (f_w).

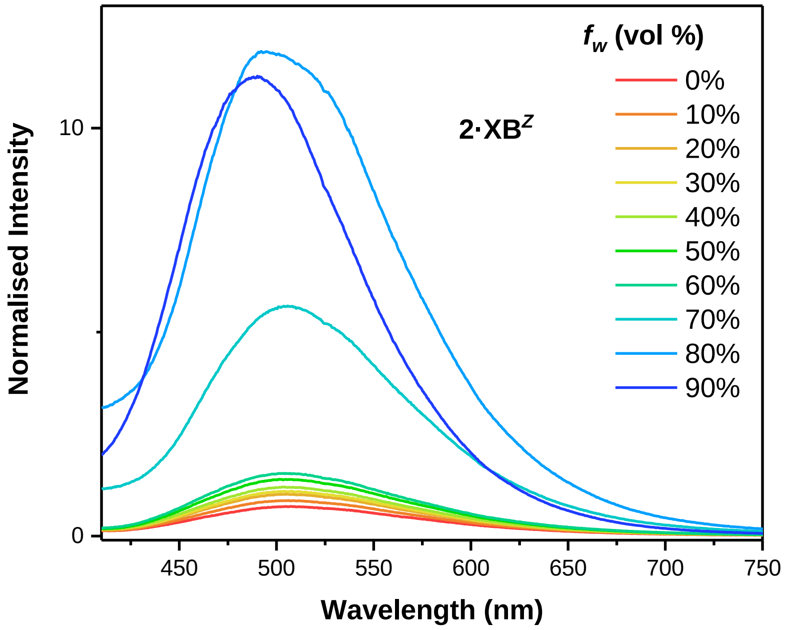

Figure S64. Fluorescence spectrum of **2·XB**^{Z} (THF, 10^{-5} M, λ_{ex} = 350 nm) with increasing water fraction (f_w).

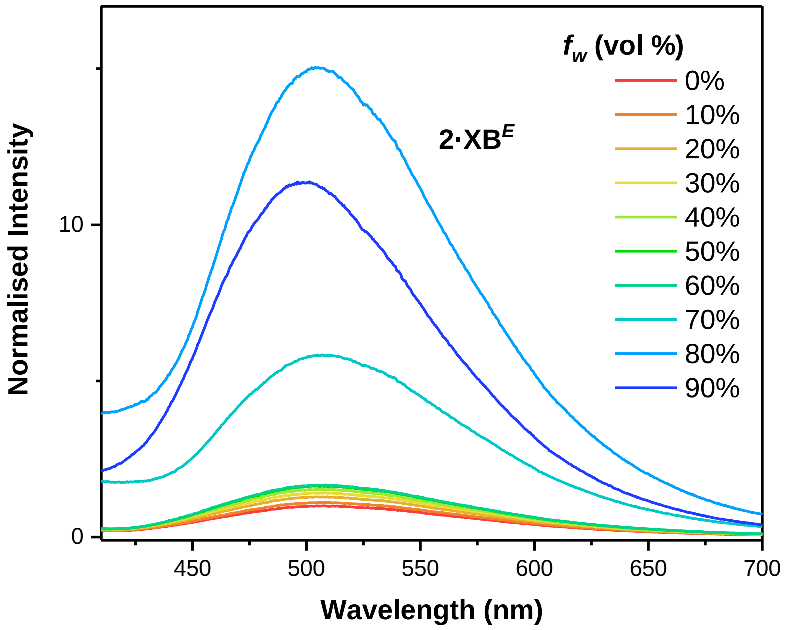

Figure S65. Fluorescence spectrum of **2·XB**^{E} (THF, 10^{-5} M, λ_{ex} = 350 nm) with increasing water fraction (f_w).

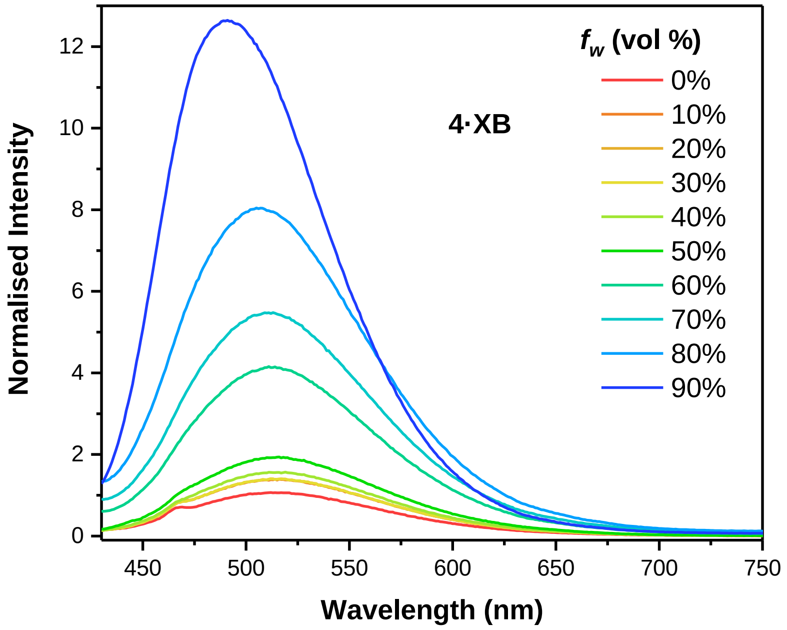

Figure S66. Fluorescence spectrum of **4·XB** (THF, 10^{-5} M, λ_{ex} = 350 nm) with increasing water fraction (f_w).

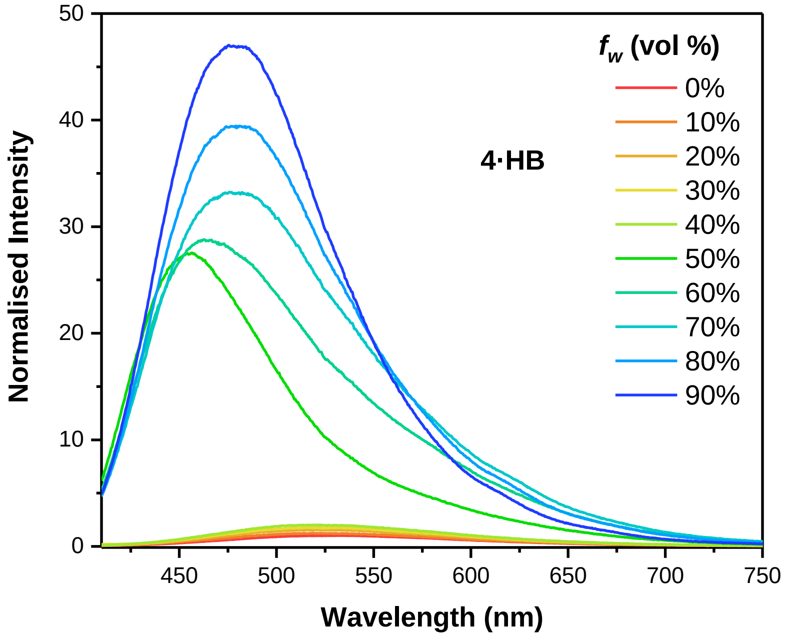

Figure S67. Fluorescence spectrum of **4·HB** (THF, 10^{-5} M, λ_{ex} = 350 nm) with increasing water fraction (f_w).

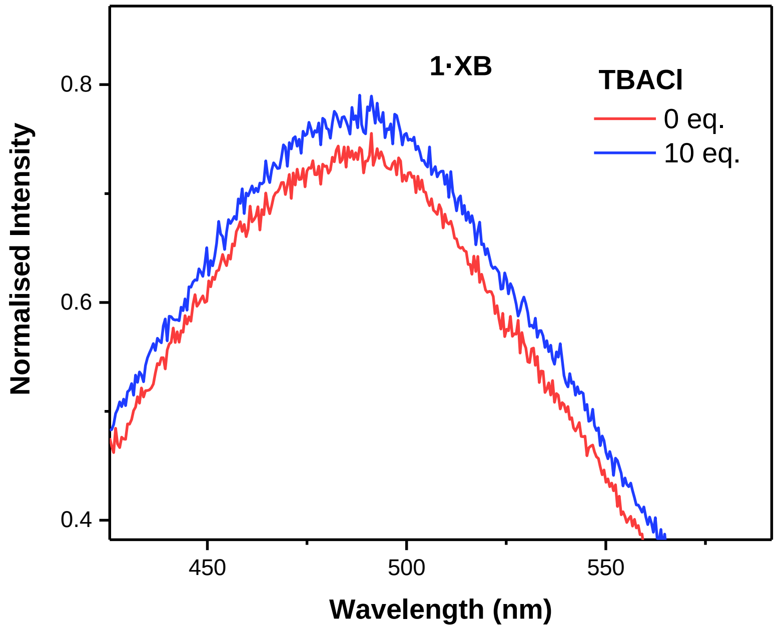

Figure S68. Fluorescence spectrum of **1·XB** (THF, 10^{-5} M, λ_{ex} = 350 nm) in the absence and prescence of 10 equivalents of TBACl.

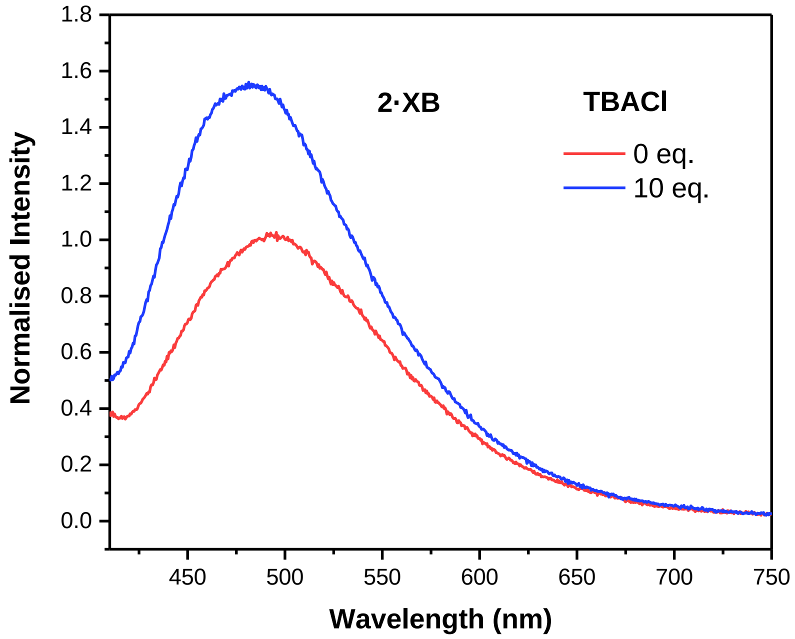

Figure S69. Fluorescence spectrum of **2·XB** (THF, 10^{-5} M, λ_{ex} = 350 nm) in the absence and prescence of 10 equivalents of TBACl.

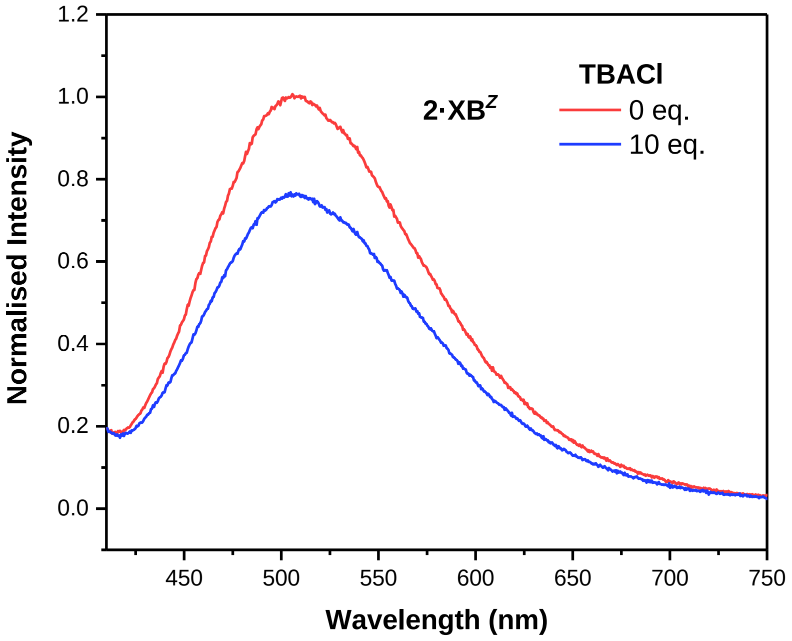

Figure S70. Fluorescence spectrum of **2·XB**^{Z} (THF, 10^{-5} M, λ_{ex} = 350 nm) in the absence and prescence of 10 equivalents of TBACl.

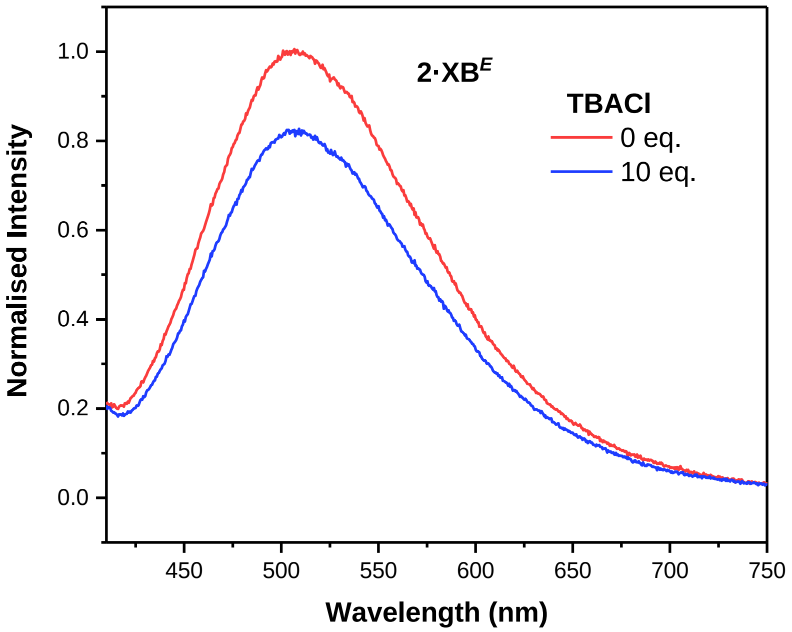

Figure S71. Fluorescence spectrum of **2·XB**^{E} (THF, 10^{-5} M, λ_{ex} = 350 nm) in the absence and prescence of 10 equivalents of TBACl.

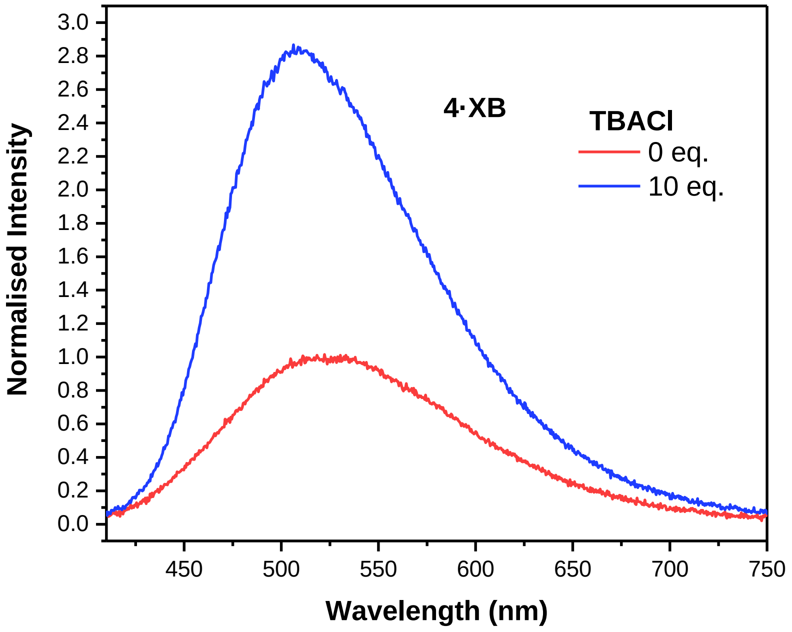

Figure S72. Fluorescence spectrum of **4·XB** (THF, 10^{-5} M, λ_{ex} = 350 nm) in the absence and prescence of 10 equivalents of TBACl.

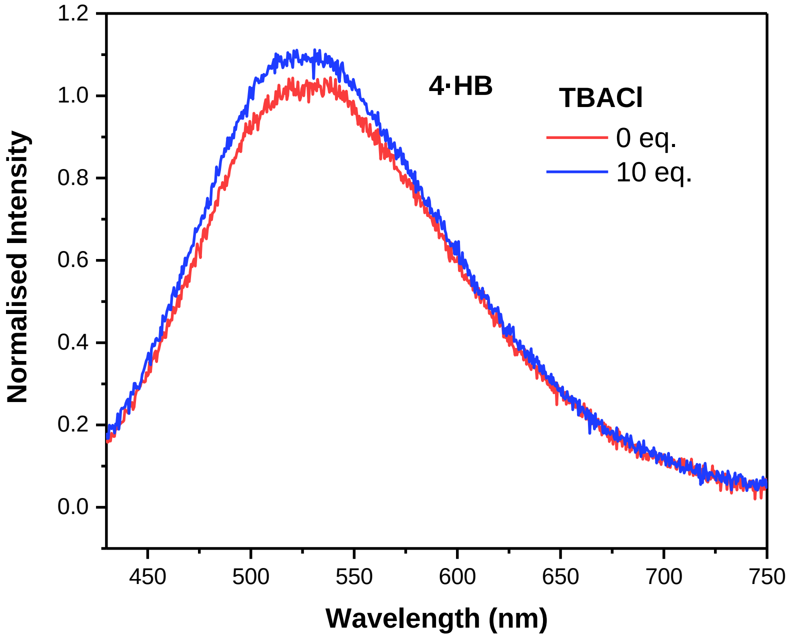

Figure S73. Fluorescence spectrum of **4·HB** (THF, 10^{-5} M, λ_{ex} = 350 nm) in the absence and prescence of 10 equivalents of TBACl.

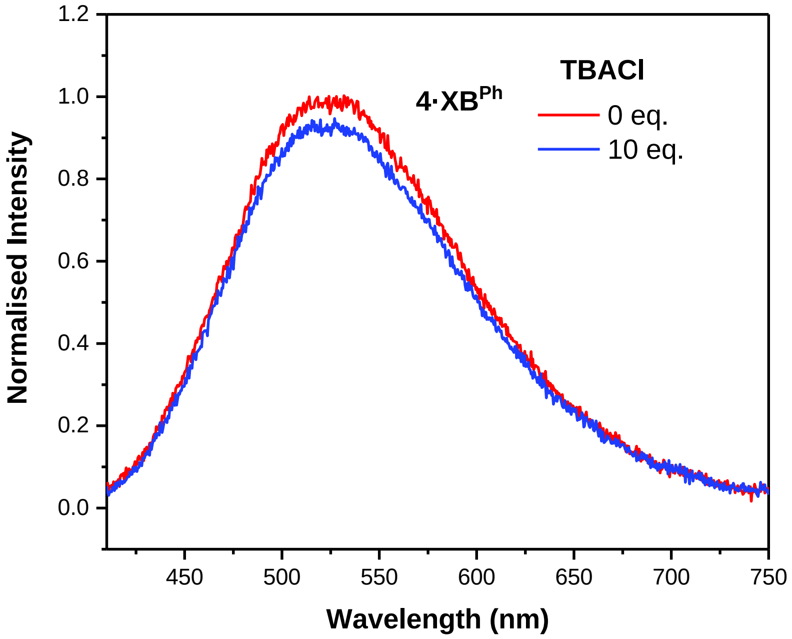

Figure S74. Fluorescence spectrum of **4·XB^{Ph}** (THF, 10^{-5} M, λ_{ex} = 350 nm) in the absence and prescence of 10 equivalents of TBACl.

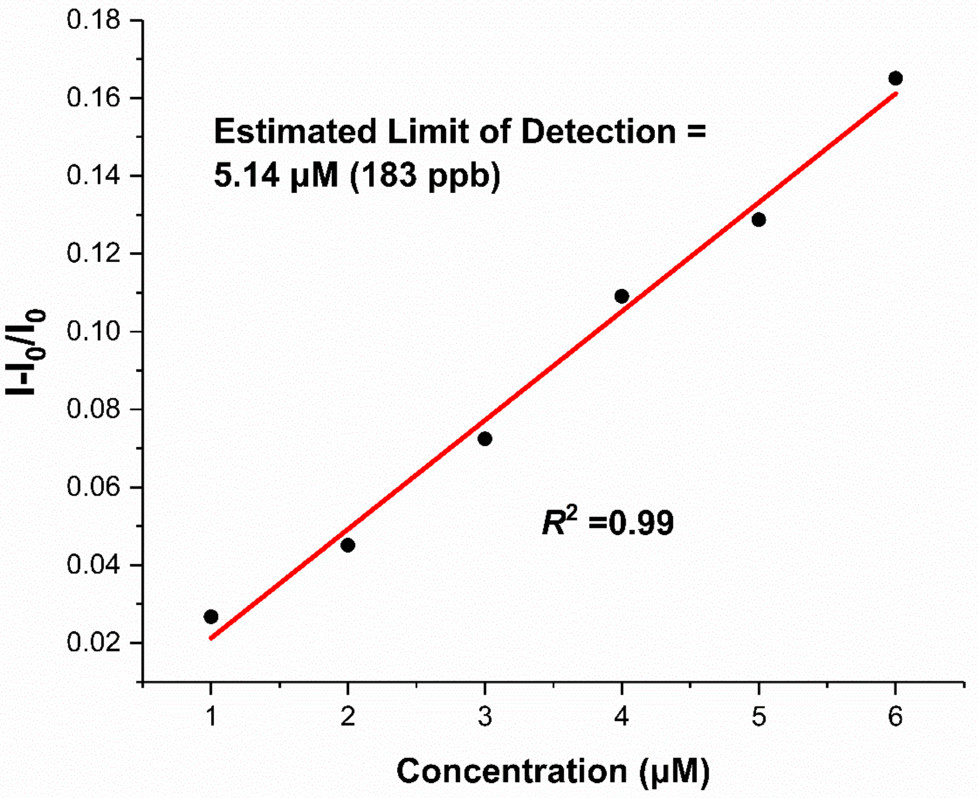

Figure S75. Limit of detection plot for **4·XB** with TBACl.

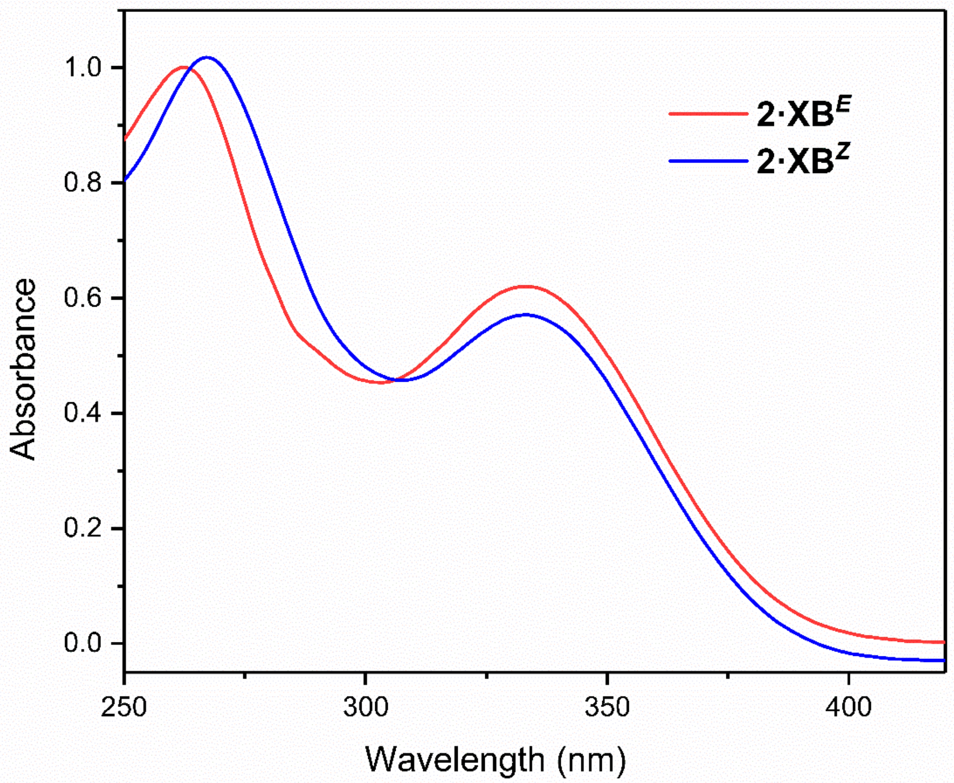

Figure S76. Overlaid UV-Vis spectrum of **2·XB**^{E} and **2·XB**^{Z} (THF, 10^{-5} M).

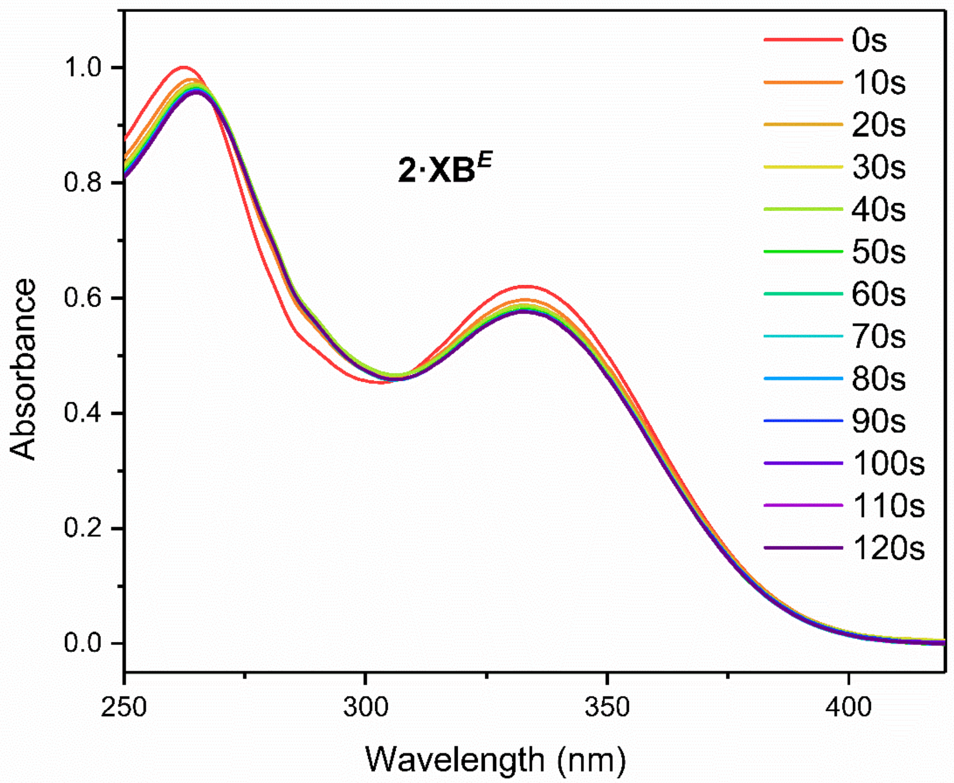

Figure S77. UV-Vis spectrum of **2·XB**^{E} (THF, 10^{-5} M), after irradiation by 405 nm.

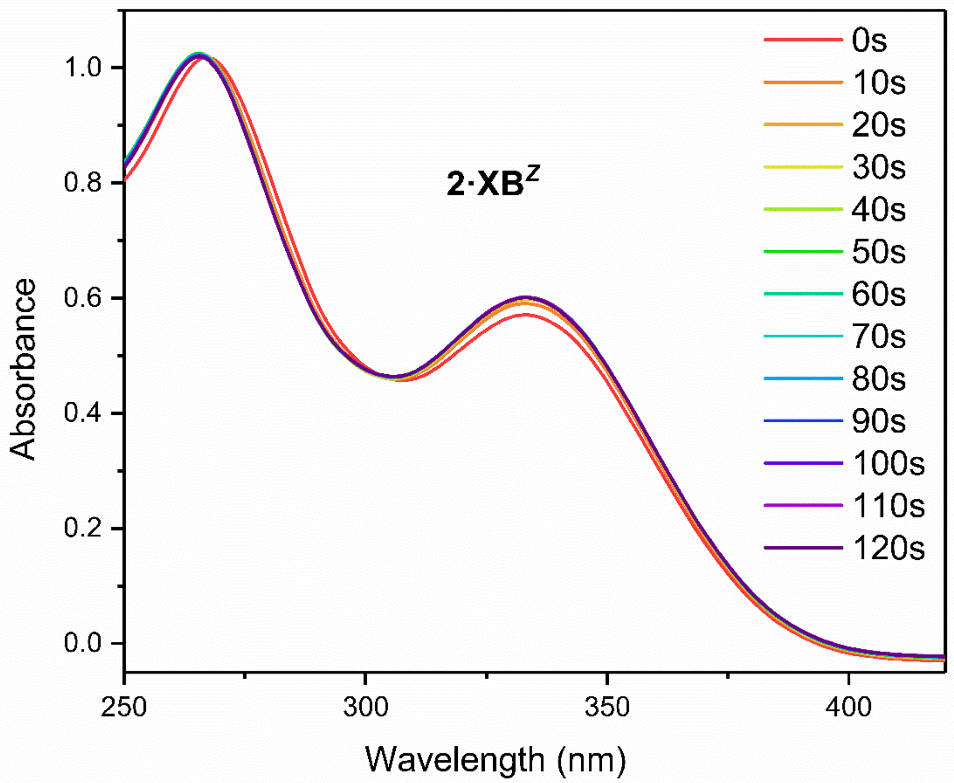

Figure S78. UV-Vis spectrum of **2·XB**^{Z} (THF, 10^{-5} M), after irradiation by 405 nm.

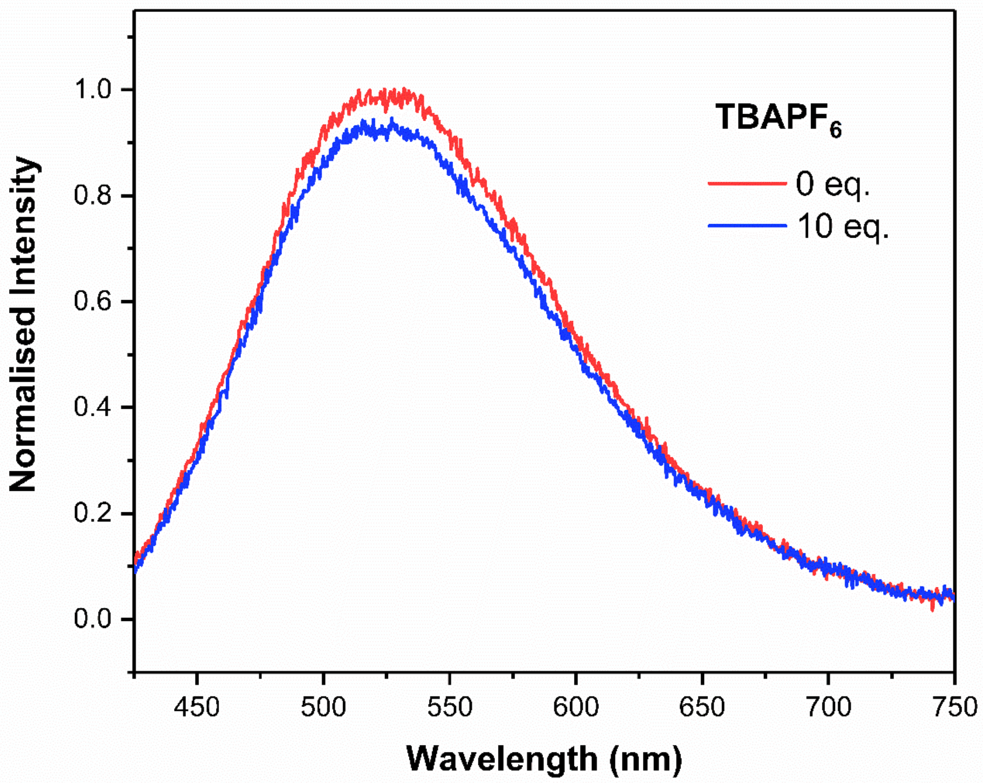

Figure S79. Fluorescence spectrum of **4·XB** (THF, 10^{-5} M, λ_{ex} = 350 nm) in the absence and prescence of 10 equivalents of $TBAPF_6$.

6. Crystal Structure Determination

Single crystals of **4·XB**, **4·XB·**NaCl and **4·XB·**NaI suitable for X-ray analysis were each coated with Paratone-N oil, suspended on a 200 µm MiTeGen loop, and placed in a cold gaseous nitrogen stream on an Oxford Diffraction Supernova X-ray diffractometer performing φ- and ω-scans at 150(2) K. Diffraction intensities were measured using graphite monochromated Cu Kα radiation (1.54184 Å).[10] Data collection, indexing, initial cell refinements, frame integration, final cell refinements and absorption corrections were accomplished using the program CrysAlisPro.[11] Scattering factors and anomalous dispersion corrections were taken from the International Tables for X-ray Crystallography. All structures were solved by direct methods and refined against F2 on all data by full-matrix least squares following established refinement strategies. **4·XB** was solved using the CRYSTALS suite[12,13] and **4·XB·**NaCl and **4·XB**·NaI were solved using SHELXS and SHELXL.[14] All non-hydrogen atoms were refined anisotropically. All hydrogen atoms were included into the model at geometrically calculated positions and refined using a riding model. Details of the data quality and a summary of the residual values for the refinements are listed in Table S4. Figures of the crystal structures have been created using the open-source PyMOL Molecular Graphics System (version 2.4.0a0, Schrödinger LLC).
.

Deposition Number(s) 2083103 (for **4·XB**) and 2082363 (for **4·XB·**NaCl) and 2082364 (for **4·XB·**NaI) contains the supplementary crystallographic data for this paper. These data are provided free of charge by the joint Cambridge Crystallographic Data Centre and Fachinformationszentrum Karlsruhe Access Structures service www.ccdc.cam.ac.uk/structures

**Compound**	**4·XB**	**4·XB·**NaCl	**4·XB·**NaI
**Formula**	C58 H16 F20 I4 N12	C58 H16 Cl F20 I4 N12 Na	C58 H16 F20 I5 N12 Na
**Formula Weight**	1768.42	1826.87	2150.69
**a (Å)**	23.0792(13)	18.0087(2)	17.8966(4)
**b (Å)**	7.1834(3)	18.0087(2)	17.8966(4)
**c (Å)**	37.023(2)	25.4560(5)	26.4769(18)
**α (º)**	90	90	90
**β (º)**	99.267(5)	90	90
**γ (º)**	90	90	90
**Unit cell volume ($Å^3$)**	6057.8(5)	8255.7(2)	8480.2(7)
**Crystal system**	Monoclinic	Tetragonal	Tetragonal
**Space group**	I 2/a	P4/ncc	P4/ncc
**Z**	4	4	4
**Temperature (K)**	150 K	150 K	150 K
**Radiation Type**	Copper	Copper	Copper
**λ (Å)**	1.54184	1.54184	1.54184
**Reflections (all)**	14804	66094	23507
**Reflections (unique)**	6245	4325	4388
**R_{int}**	0.056	0.0686	0.0709
**$R[I > 2\sigma(I)]$**	0.1196	0.1119	0.1146
**$wR(F^2)$ (all data)**	0.3346	0.2794	0.3909
**S**	1.245	1.207	1.234

Table S4. Selected Crystallographic and Refinement Data for X-ray Crystal Structures of **4·XB**, **4·XB·**NaCl and **4·XB·**NaI.

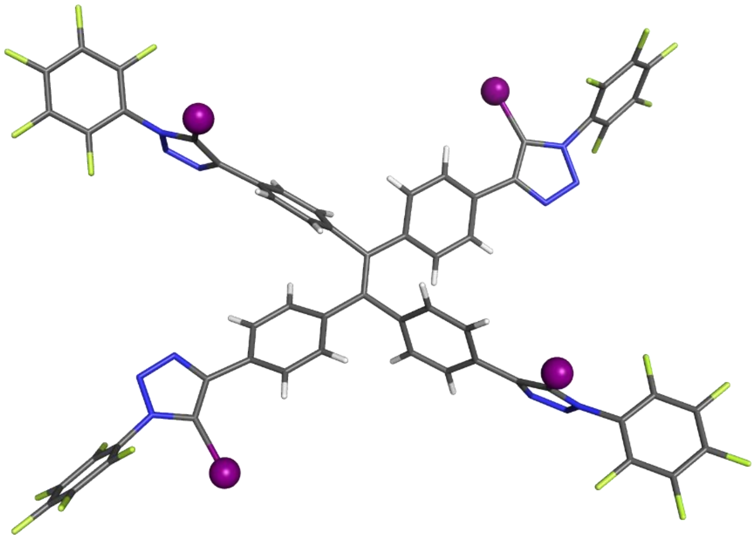

Figure S80. Solid state structure of the **4·XB**. Grey = carbon, blue = nitrogen, white = hydrogen, light green = fluorine, iodine = purple.

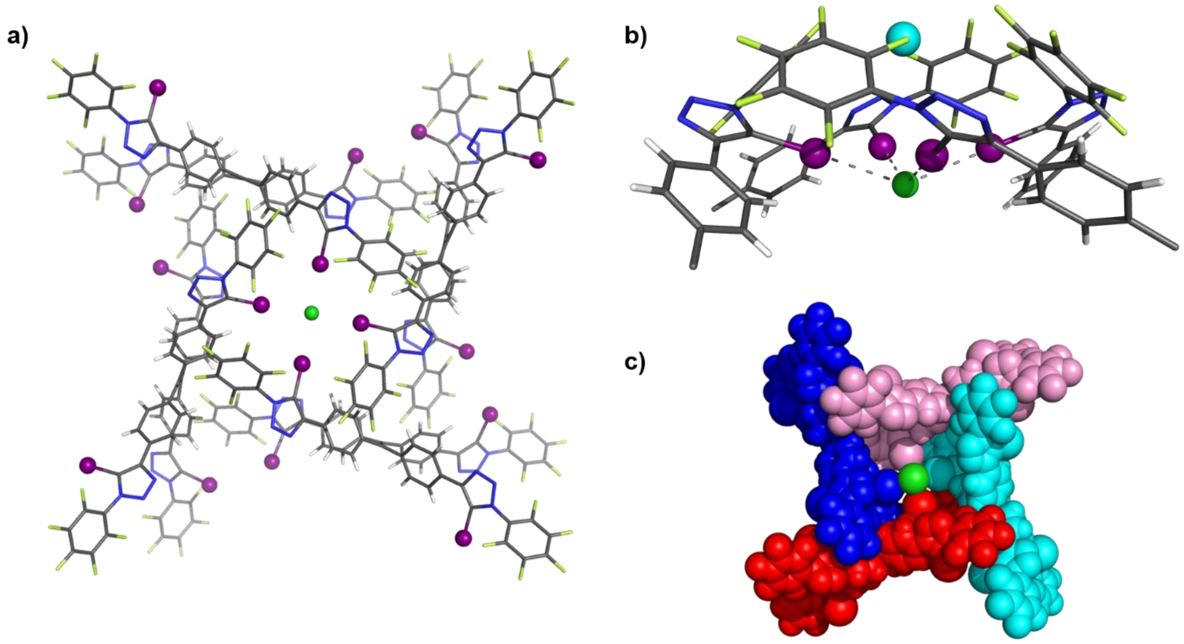

Figure S81. a) Solid state structure of the **4·XB**·NaCl, presented as a wire frame structure. b) Truncated view of chloride coordination in **4·XB**·NaCl. c) Space filling representation of **4·XB**·NaCl. Grey = carbon, blue = nitrogen, white = hydrogen, light green = fluorine, iodine = purple, green = chloride, cyan = sodium.

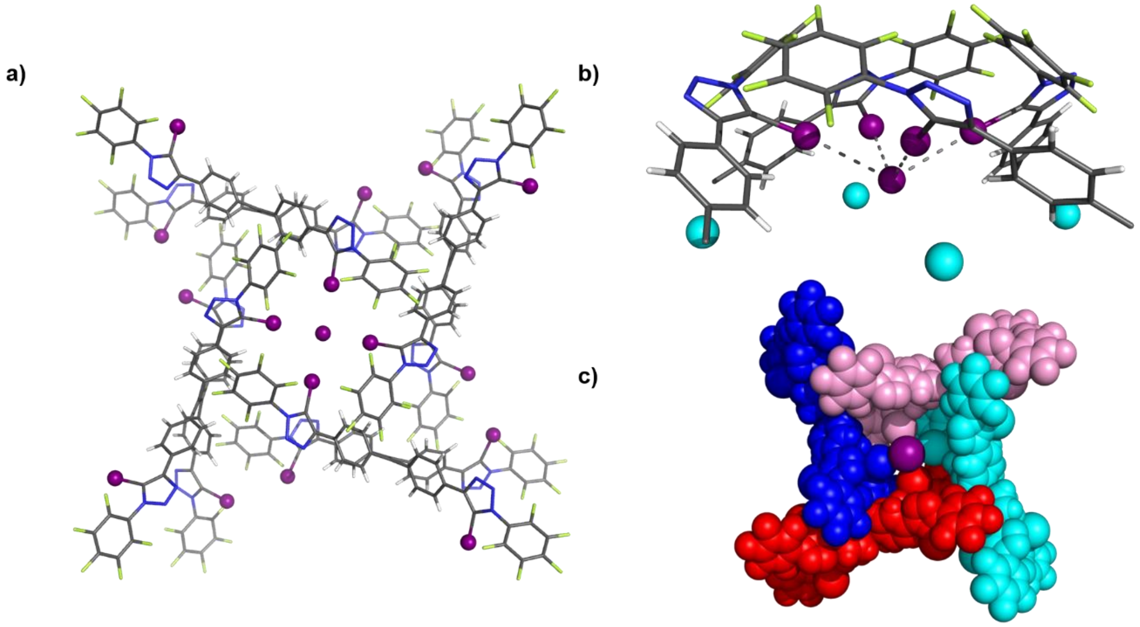

Figure S82. a) Solid state structure of the **4·XB**·NaI, presented as a wire frame structure. b) Truncated view of iodide coordination in **4·XB**·NaI. c) Space filling representation of **4·XB**·NaCl. Grey = carbon, blue = nitrogen, white = hydrogen, green = fluorine, iodine = purple, cyan = sodium.

Selected unwarp images generated using the CrysAlisPro suite have been included for the **4·XB** dataset. (Figure S82) These images show some diffuse scattering and a drop off in the intensity for the higher angle data, which both contribute to the slightly lower quality of data. Figure S83, which was generated using CCDC Mercury, shows the terminal perfluorobenzene units with the largest displacement parameters packing together, which could be a possible source of the diffuse scattering.[15] However, all atom position could be clearly located and all atom types have been assigned with high confidence. The data therefore allows for a clear confirmation of the connectivity of the structure.

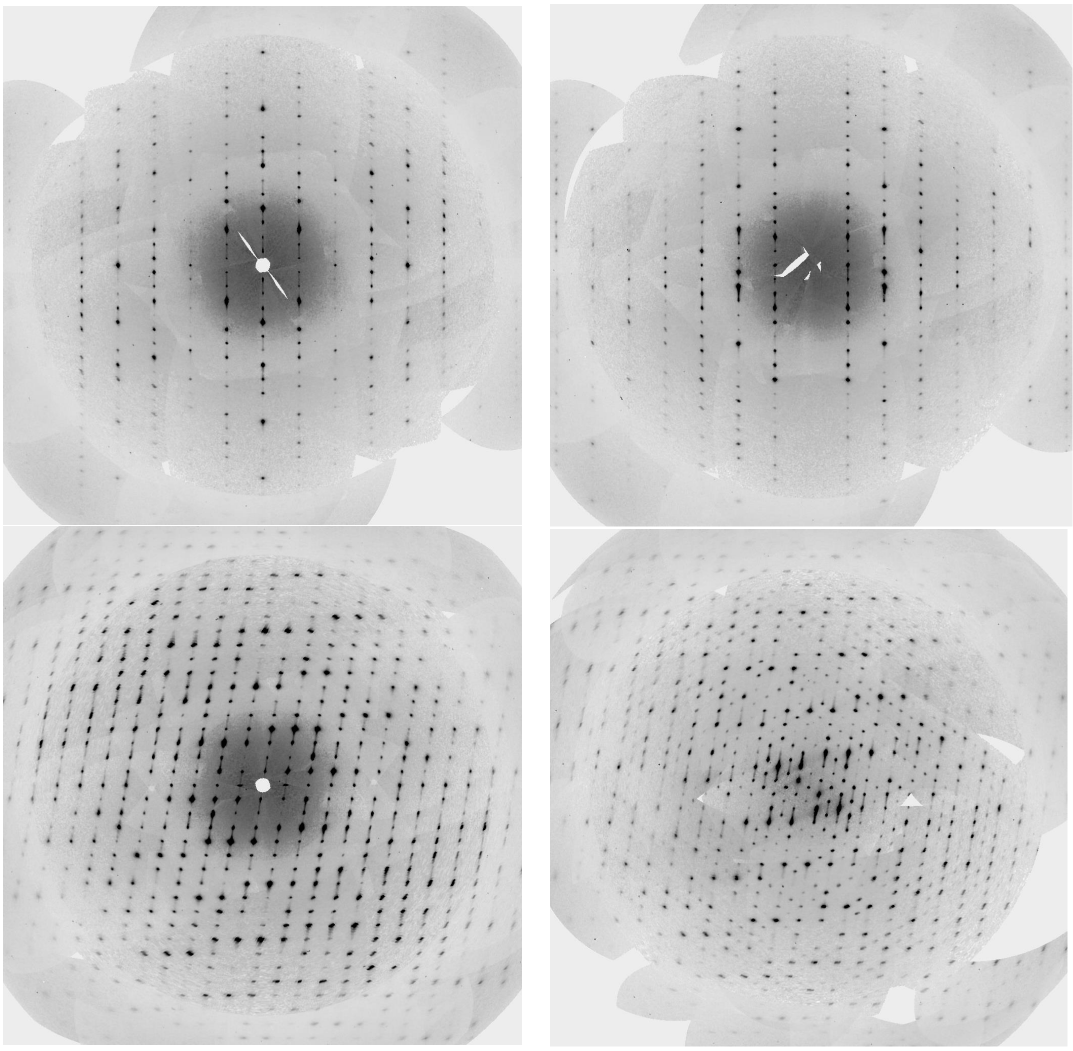

Figure S83. Unwarp images for the (left to right) 0kl, 1kl, h0l and h2l layers for the **4·XB** dataset

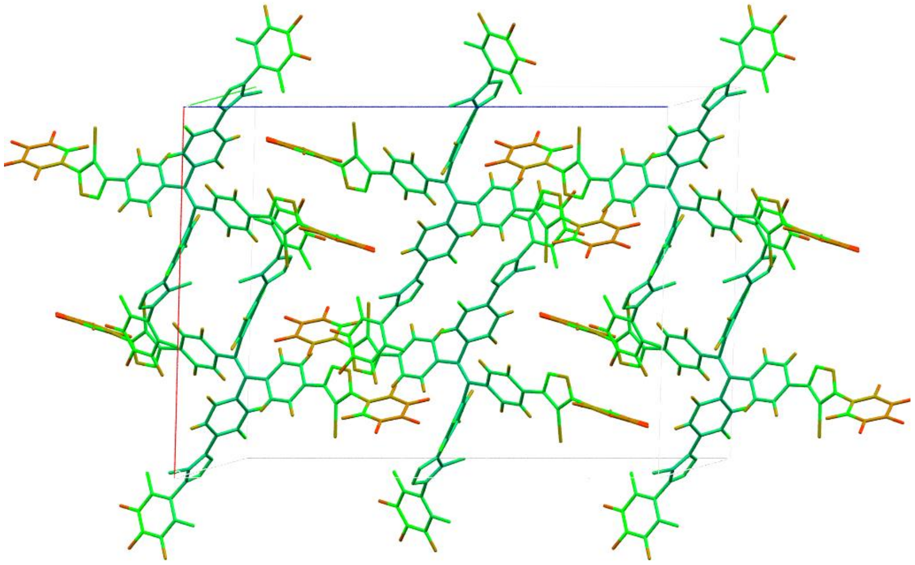

Figure S84. Packing within the **4·XB** solid state structure with atoms coloured by atomic displacement parameters. Gradient, smallest displacement in dark green to largest displacement in red.

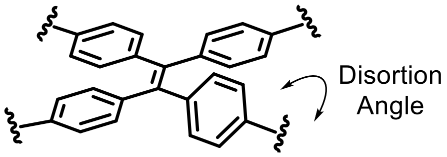

Figure S85. Truncated diagram of the TPE core of **4·XB** and indicated angle of the distortion.

Table S5. Summary of angles of distortion for **4·XB** and **4·XB**·NaCl, uncertainties indicated in parentheses.

	Distortion angle(s)/ °
**4·XB**	62.0 (0.2)
	27.1 (1.3)
**4·XB**·NaCl	56.2 (0.4)

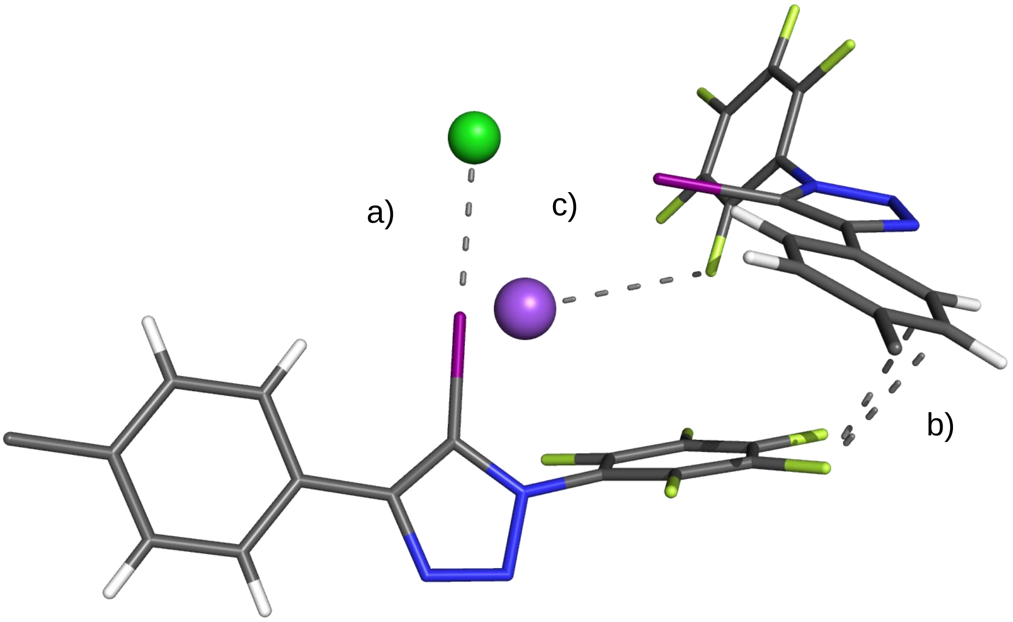

Figure S86. Truncated view of solid state structure of **4·XB**·NaCl, presented as a wire frame structure. Grey = carbon, blue = nitrogen, white = hydrogen, green = fluorine, iodine = purple, cyan = sodium. Demonstrating the formation of a) C-I···Cl^- XB interactions b) C-F···π interactions and c) C-F···Na^+ interactions all of which could potentially contribute to the stability of the aggregate structure in the solid state.

7. Transmission Electron Microscopy (TEM) Imaging

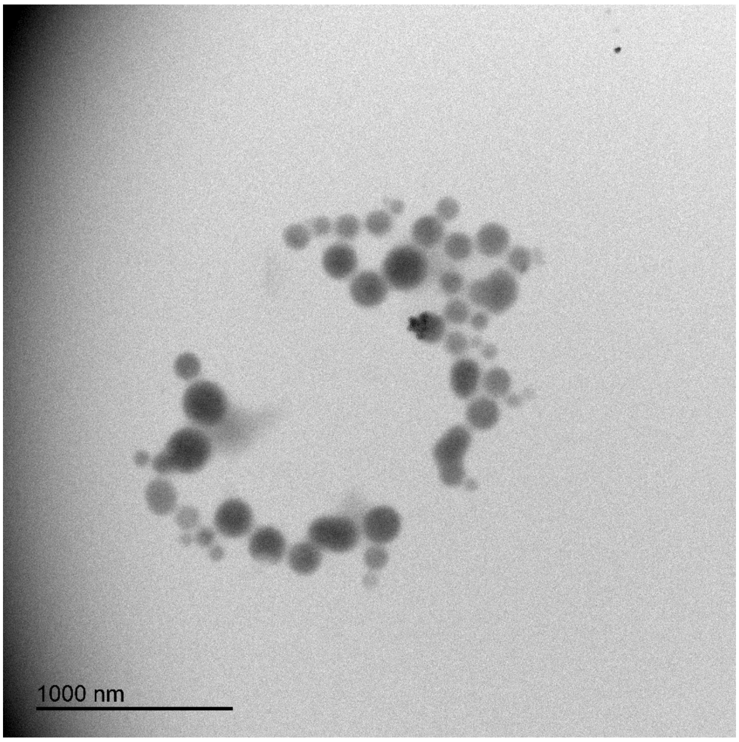

Figure S87. TEM image of **4·XB** in the presence of 1 eq. of TBACl added as a THF solution (10^{-3} M).

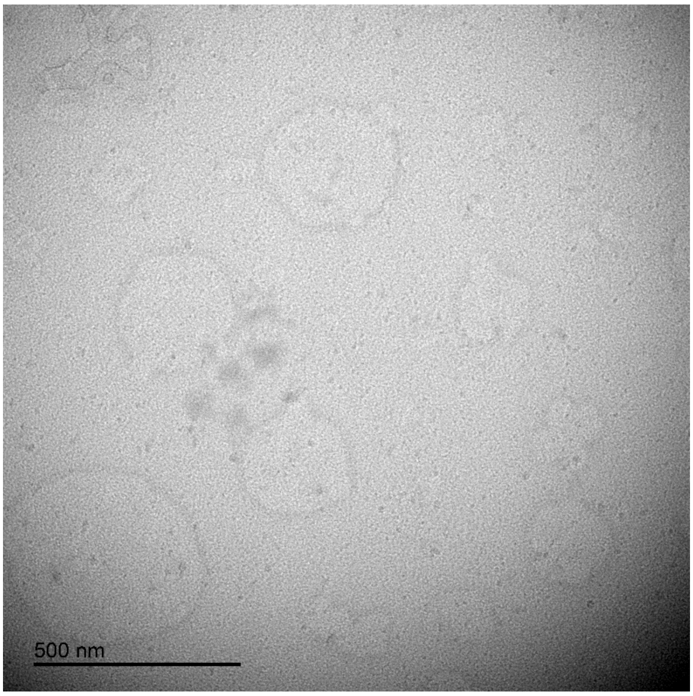

Figure S88. TEM image of **4·XB** in the absence of TBACl.

8. References

[1] M. I. Mangione, R. A. Spanevello, M. B. Anzardi, *RSC Adv.* **2017**, *7*, 47681–47688.

[2] L.-M. Jin, X. Xu, H. Lu, X. Cui, L. Wojtas, X. P. Zhang, *Angewandte Chemie International Edition* **2013**, *52*, 5309–5313.

[3] A. Kerckhoffs, M. J. Langton, *Chem. Sci.* **2020**, *11*, 6325–6331.

[4] F. G. Erko, L. Cseh, J. Berthet, G. H. Mehl, S. Delbaere, *Dyes and Pigments* **2015**, *115*, 102–109.

[5] J. Huang, Y. Jiang, J. Yang, R. Tang, N. Xie, Q. Li, H. S. Kwok, B. Z. Tang, Z. Li, *J. Mater. Chem. C* **2014**, *2*, 2028–2036.

[6] J. Du, S. Yu, Z. Huang, L. Chen, Y. Xu, G. Zhang, Q. Chen, X. Yu, L. Pu, *RSC Adv.* **2016**, *6*, 25319–25329.

[7] Y. Matt, I. Wessely, L. Gramespacher, M. Tsotsalas, S. Bräse, *European Journal of Organic Chemistry* **2021**, *2021*, 239–245.

[8] Z. Wang, C.-Y. Zhu, J.-T. Mo, P.-Y. Fu, Y.-W. Zhao, S.-Y. Yin, J.-J. Jiang, M. Pan, C.-Y. Su, *Angewandte Chemie International Edition* **2019**, *58*, 9752–9757.

[9] D. B. Hibbert, P. Thordarson, *Chem. Commun.* **2016**, *52*, 12792–12805.

[10] J. Cosier, A. M. Glazer, *J Appl Crystallogr* **1986**, *19*, 105–107.

[11] *CrysAlisPRO*, Oxford Diffraction /Agilent Technologies UK Ltd, Yarnton, England., **n.d.**

[12] P. W. Betteridge, J. R. Carruthers, R. I. Cooper, K. Prout, D. J. Watkin, *J Appl Cryst* **2003**, *36*, 1487–1487.

[13] P. Parois, R. I. Cooper, A. L. Thompson, *Chemistry Central Journal* **2015**, *9*, 30.

[14] G. M. Sheldrick, *Acta Cryst A* **2008**, *64*, 112–122.

[15] C. F. Macrae, I. Sovago, S. J. Cottrell, P. T. A. Galek, P. McCabe, E. Pidcock, M. Platings, G. P. Shields, J. S. Stevens, M. Towler, P. A. Wood, *J Appl Cryst* **2020**, *53*, 226–235.
